# Causality between allergic diseases and kidney diseases: a two-sample Mendelian randomization study

**DOI:** 10.3389/fmed.2024.1347152

**Published:** 2024-03-12

**Authors:** Zhe Peng, Xinyu Dong, Yingxin Long, Zunjiang Li, Yueyao Wang, Wei Zhu, Banghan Ding

**Affiliations:** ^1^The Second Clinical College of Guangzhou University of Chinese Medicine, Guangzhou, Guangdong, China; ^2^Guangdong Provincial Hospital of Chinese Medicine, Guangzhou, Guangdong, China; ^3^Science and Technology Innovation Center, Guangzhou University of Chinese Medicine, Guangzhou, Guangdong, China; ^4^College of Pharmacy, Jinan University, Guangzhou, Guangdong, China; ^5^Guangdong Provincial Key Laboratory of Research on Emergency in Traditional Chinese Medicine, Guangzhou, Guangdong, China

**Keywords:** allergic diseases, kidney diseases, Mendelian randomization, causality, reverse-direction, GWAS

## Abstract

**Background:**

Evidence from observational studies and clinical trials suggests that the allergic diseases (ADs) are associated with kidney diseases (KDs). However, the causal association between them remains to be determined. We used bidirectional two-sample Mendelian randomization (MR) analysis to evaluate the potential causality between them.

**Methods:**

Mendelian randomization (MR) was performed using publicly available genome-wide association study (GWAS) summary datasets. Inverse variance weighted (IVW), weighted median, MR-Egger regression, simple mode, and weighted mode methods are used to evaluate the causality between ADs and KDs. Sensitivity and heterogeneity analyses were used to ensure the stability of the results.

**Results:**

The MR results indicated that genetic susceptibility to ADs was associated with a higher risk of CKD [odds ratio (OR) = 1.124, 95% CI = 1.020–1.239, *p* = 0.019] and unspecified kidney failure (OR = 1.170, 95% CI = 1.004–1.363, *p* = 0.045) but not with kidney stone, ureter stone or bladder stone (OR = 1.001, 95% CI = 1.000–1.002, *p* = 0.216), other renal or kidney problem (OR = 1.000, 95% CI = 1.000–1.001, *p* = 0.339), urinary tract or kidney infection (OR = 1.000, 95% CI = 0.999–1.001, *p* = 0.604), kidney volume (OR = 0.996, 95% CI = 0.960–1.033, *p* = 0.812) and cyst of kidney (OR = 0.914, 95% CI = 0.756–1.105, *p* = 0.354). No causal evidence of KDs on ADs was found in present study.

**Conclusion:**

Results from MR analysis indicate a causal association between ADs and CKD and unspecified kidney failure. These findings partly suggest that early monitoring of CKD risk in patients with ADs is intentional.

## Introduction

Allergic diseases (AD) are a category of diseases caused by abnormal immune responses to external environmental stimuli (1) by the body. In recent years, there has been a consistent upward trend (2, 3) in the incidence of these diseases. Common clinical manifestations include allergic rhinitis (4), asthma (5), eczema (6), and atopic dermatitis. The pathogenesis involves genetic factors (7, 8) and immune system abnormalities.

Kidney diseases (KD) constitute a collective term encompassing various types such as chronic kidney disease (CKD), diabetic nephropathy and more (9–14). CKD is a condition characterized by gradual kidney damage (15), often accompanied by a progressive loss of kidney function (16). Epidemiological data indicates that CKD causes 5–10 million deaths annually (17) and the prevalence of kidney stone is as high as 11% in the United States, 9% in Europe (18). CKD is divided into five stages, ranging from mild to end-stage renal disease (ESRD) (19). ESRD presents more severe symptoms (20) and typically necessitates dialysis or kidney transplantation.

Some studies have found a correlation between ADs and KDs. For instance, systemic lupus erythematosus (SLE), as an autoimmune disease, can lead to renal impairment, which is defined as “SLE nephritis (21).” But whether there is a causal relationship between ADs and KDs is still unknown. It is thus important to explore the causal relationship between them.

With the rapid advancement of various omics, including genomics and high-throughput sequencing technologies, biomedical data has shown exponential growth. This has led to the establishment of vast, high-quality databases, ushering in a new era where data drives medical progress. The fusion of big data and artificial intelligence algorithms provides a novel perspective on understanding the causative relationships between diseases and various risk factors. It offers new methods for delving into disease mechanisms. MR analysis is a method based on whole-genome sequencing to assess causality between exposure factors and outcomes (22). It can achieve the same level of evidence as randomized controlled trials (RCT), providing an approach that circumvents potential confounding factors or reverse causation bias (23). It also overcomes the time-consuming, resource-intensive, and costly nature of RCT (24), making it one of the most promising methods for evaluating causality (25).

This study collected publicly available published data and utilized bidirectional two-sample MR analysis to investigate the causality between ADs and KDs.

## Materials and methods

### Study design

**FIGURE 1:**
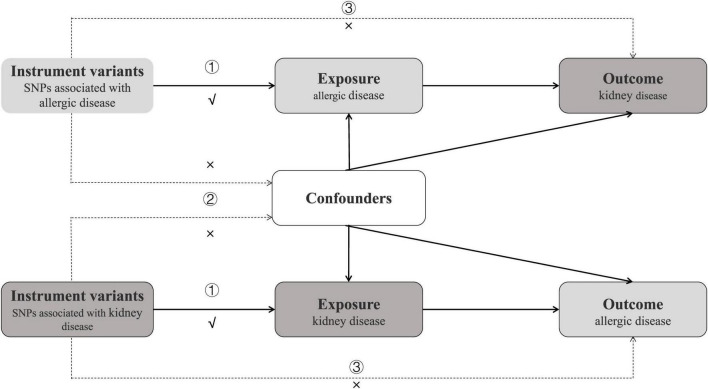
Study design of bidirectional Mendelian randomization study between ADs and 7 kinds of KDs. The instrumental variables meet the three conditions: ① the variables should be highly associated with exposure; ① the variables should be independent of confounding factors of the exposure-outcome association; ② the variables affect the outcome only via the exposure pathway and not through other biological pathways.

We performed a total of 21 MR analyses to investigate the bidirectional association between ADs and KDs. The analysis primarily satisfies the following three major assumptions based on the characteristics of MR: (1) strong correlation between instrumental variables (IVs) and exposure factors; (2) independence of IVs from confounding factors related to the outcome; (3) IVs are associated with the outcome only through exposure factors (Figure 1). Our analyses are restricted to most of the participants of European descent to minimize racial mismatches.

### Data sources

Summary statistics data for ADs were derived from UK Biobank,^1^ including 180129 cases and 180709 controls (26). GWAS data for kidney stone, ureter stone or bladder stone (27) were derived from UK Biobank (ebi-a-GCST90038631), including 3,725 cases of European origin and 480,873 controls. Single-nucleotide polymorphisms (SNPs) for other renal or kidney problem (27) were derived from UK Biobank (ebi-a-GCST90038666), including 2,609 cases and 481,989 controls. Summary-level data for urinary tract or kidney infection (27) were derived from UK Biobank that included 2,691 cases and 481,907 controls. Summary statistics for kidney volume (28) were derived from UK Biobank, including 32,860 samples. The summary dataset for cyst of kidney was from FinnGen,^2^ including 739 cases and 217,185 controls. SNPs for CKD were derived from FinnGen (finn-b-N14_CHRONKIDNEYDIS), including 3,902 cases and 212,841 controls. GWAS data for unspecified kidney failure were derived from FinnGen (finn-b-N14_RENFAILNAS), including 963 cases and 212,841 controls. An overview of the demographics involved in this study is shown in Table 1. The reverse MR analyses used the summary statistics data with ADs as outcome, and KDs as exposure.

**TABLE 1:** Data sources.

	Traits	GWAS ID	Year	Case/control	nSNP
1	Allergic disease (asthma, hay fever or eczema)	ebi-a-GCST005038	2017	180,129/180,709	8,133,670
2	Kidney stone, ureter stone or bladder stone	ebi-a-GCST90038631	2021	3,725/480,873	9,587,836
3	Other renal or kidney problem	ebi-a-GCST90038666	2021	2,609/481,989	9,587,836
4	Urinary tract or kidney infection	ebi-a-GCST90038630	2021	2,691/481,907	9,587,836
5	Kidney volume	ebi-a-GCST90016670	2021	–	9,275,407
6	Cyst of kidney	finn-b-N14_CYSTKID	2021	739/217,185	16,380,463
7	Chronic kidney disease	finn-b-N14_CHRONKIDNEYDIS	2021	3,902/212,841	16,380,459
8	Unspecified kidney failure	finn-b-N14_RENFAILNAS	2021	963/212,841	16,380,451

### Ethics approval

Our analysis used publicly available GWAS summary statistics. No new data were collected, and no new ethical approval was required.

### Selection of IVs for MR analyses

**FIGURE 2:**
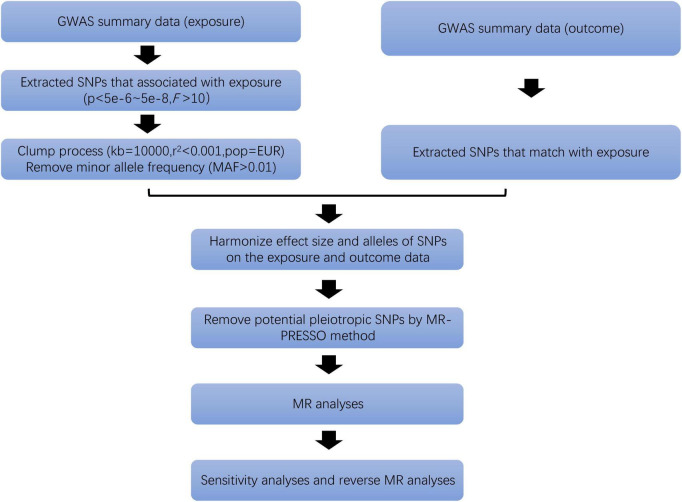
Flow chart of SNPs selection.

Only SNPs associated at genome-wide significance *P*-value (*P* < 5 × 10^–8^) with a minor allele frequency (MAF) greater than 0.01 were considered as potential IVs (29). Independent SNPs were selected according to linkage disequilibrium (LD) (*r*^2^ < 0.001, kb = 10,000). At the same time, the strength of IVs is tested by F statistics (*F* > 10) to ensure that the obtained IVs are strongly related to exposure factors (30). Then, we deleted palindromic SNPs and harmonized exposure-outcome datasets to avoid distortion of strand orientation or allele coding (Figure 2). Finally, we recorded the information of the selected IVs, such as effect allele (EA), beta, standard error (SE), effect allele frequency (eaf) and *p*-value (Supplementary Table 1).

### Two-sample MR analysis

For binary exposures, the casual estimate was presented as an odds ratio (OR) and 95% confidence interval (CI) per log-odds increment in genetically determined risk of the exposures. MR analyses utilized the random-effects inverse-variance weighted (IVW) method as the primary method to estimate the potential bidirectional causal associations between ADs and KDs as it provides a robust causal estimate in the absence of directional pleiotropy (31). Furthermore, we used weighted median, simple mode, weight mode, and MR-Egger (32) methods for alternative analyses. We used MR Egger intercept, MR-PRESSO global test, and Cochran Q statistic to test the presence of heterogeneity or directional pleiotropy. Moreover, visual inspection of the forest plot, funnel plot, and scatter plot were also used to assess the MR “no horizontal pleiotropy” assumption. In addition, sensitivity analysis by applying leave-one-out analysis was conducted. All statistical analyses were performed using the two-sample MR packages in R (version 4.3.1).^3^ We eliminated the influence of confounding factors by using the Phenoscanner database.^4^

### Reverse MR analyses

To explore whether KDs have any causal impact on ADs, we also performed a reverse MR analysis (i.e., KDs as the exposure and ADs as the outcome) using SNPs that are associated with KDs as IVs.

The threshold selected SNPs less than the genome-wide statistical significance threshold (5 × 10^–8^) to serve as IVs. Unfortunately, only a few SNPs were selected when we set this condition. To explore more relations between KDs and ADs, we used the second threshold that identified SNPs that were smaller than the genome-wide significance threshold (5 × 10^–6^) and selected them as the second IVs set to find more potential causality.

### GO and KEGG pathway analyses

To collect the potential targets of ADs and KDs, we searched the results from the GeneCards database^5^ using the keywords “allergic disease,” “kidney disease,” and applied “relevance score > 10” as a screening criterion. The intersection of their targets was obtained using Venny2.1.0.^6^ To further analyze the potential targets, we imported them into the DAVID database^7^ for Gene Ontology (GO) and Kyoto Encyclopedia of Genes and Genomes (KEGG) pathway enrichment. The results were visualized by pictures.

## Results

### SNPs selection

For the first four kinds of KDs (ebi-a-GCST90038631, ebi-a-GCST90038666, ebi-a-GCST90038630, ebi-a-GCST90016670), we found that 1 SNP of the 74 AD-related SNPs was not available in ebi-a-GCST90038631, 2 SNPs were not available in ebi-a-GCST90038630 and 5 SNPs were not available in ebi-a-GCST90016670. For the latter three (finn-b-N14_CYSTKID, finn-b-N14_CHRONKIDNEYDIS, finn-b-N14_RENFAILNAS), we found that 4 SNPs of 72 AD-related SNPs were not available in finn-b-N14_CYSTKID. At the same time, we excluded 6 improper SNPs in finn-b-N14_CHRONKIDNEYDIS and finn-b-N14_RENFAILNAS. In addition, we all excluded 3 palindrome sequences (rs11658582, rs301802, rs760805). Therefore, we finally included 70, 71, 69, 66, 65, 63, and 63 SNPs as IVs to analyze. The *F* statistics of the IVs were all largely greater than 10 (Supplementary Table 2), indicating no evidence of weak instrument bias was detected. Eventually, after removing pleiotropic SNPs identified by the MR-PRESSO test and the MR-Egger regression, there was no evidence of horizontal pleiotropy of the IVs.

### Causality of ADs and 7 KDs

(1) Kidney stone, ureter stone or bladder stone

**FIGURE 3:**
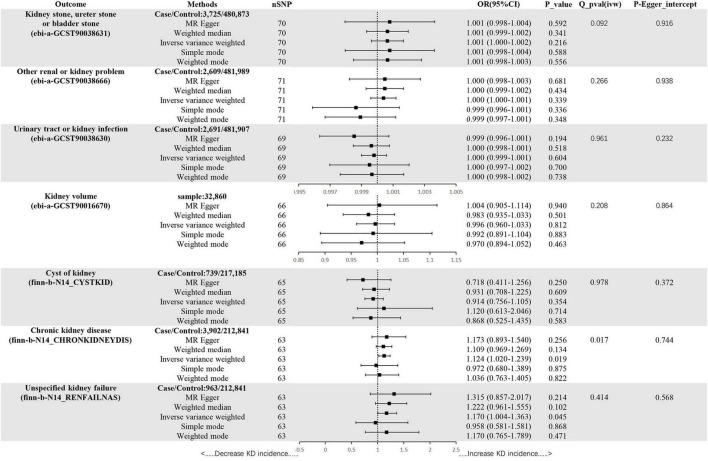
Mendelian randomization estimates of ADs on KDs. SNPs, single-nucleotide polymorphisms; OR, odds ratio; CI, confidence interval; Q, heterogeneity statistic Q.

In the set of IVs (*p* < 5 × 10^–8^), we found no genetic liability to ADs that was causally associated with kidney stone, ureter stone or bladder stone (OR = 1.001, 95% CI = 1.000–1.002, *p* = 0.216, IVW) (Figure 3).

(2) Other renal or kidney problem

In the set of IVs (*p* < 5 × 10^–8^), we found no genetic liability to ADs that was causally associated with other renal or kidney problem (OR = 1.000, 95% CI = 1.000–1.001, *p* = 0.339, IVW) (Figure 3).

(3) Urinary tract or kidney infection

In the set of IVs (*p* < 5 × 10^–8^), we found no genetic liability to ADs that was causally associated with urinary tract or kidney infection (OR = 1.000, 95% CI = 0.999–1.001, *p* = 0.604, IVW) (Figure 3).

(4) Kidney volume

In the set of IVs (*p* < 5 × 10^–8^), we found no genetic liability to ADs that was causally associated with kidney volume (OR = 0.996, 95% CI = 0.960–1.033, *p* = 0.812, IVW) (Figure 3).

(5) Cyst of kidney

In the set of IVs (*p* < 5 × 10^–8^), we found no genetic liability to ADs that was causally associated with cyst of kidney (OR = 0.914, 95% CI = 0.756–1.105, *p* = 0.354, IVW) (Figure 3).

(6) Chronic kidney disease

In the set of IVs (*p* < 5 × 10^–8^), we found ADs were causally associated with CKD (OR = 1.124, 95% CI = 1.020–1.239, *p* = 0.019, IVW) (Figure 3).

(7) Unspecified kidney failure

In the set of IVs (*p* < 5 × 10^–8^), we found ADs were causally associated with unspecified kidney failure (OR = 1.170, 95% CI = 1.004–1.363, *p* = 0.045, IVW) (Figure 3).

### Sensitivity analyses

The MR-Egger, weighted mode, simple mode and weighted median methods yielded similar causal estimates for magnitude and direction (Figure 3). We found no evidence of horizontal pleiotropy for ADs in KDs when using the MR-Egger regression intercept approach. Although a Cochrane Q result (ADs-CKD) showed heterogeneity (*p* < 0.05), the result of MR-PRESSO revealed no outliers, so we chose IVW as the main method. Other results showed no heterogeneity. It can be seen from the leave-one–out figure that when any SNP is excluded for analysis, the results are stable (Supplementary Figures 1–4).

### Reverse-direction MR analyses

To evaluate reverse causality, we used KDs as exposure and ADs as outcome. SNPs associated with KDs extracted from previous GWAS were used as IVs (Supplementary Table 3). We found that 7 KDs were not causally associated with ADs. The relevant analysis results are shown in the Supplementary Table 4.

### GO and KEGG pathway analyses

**FIGURE 4:**
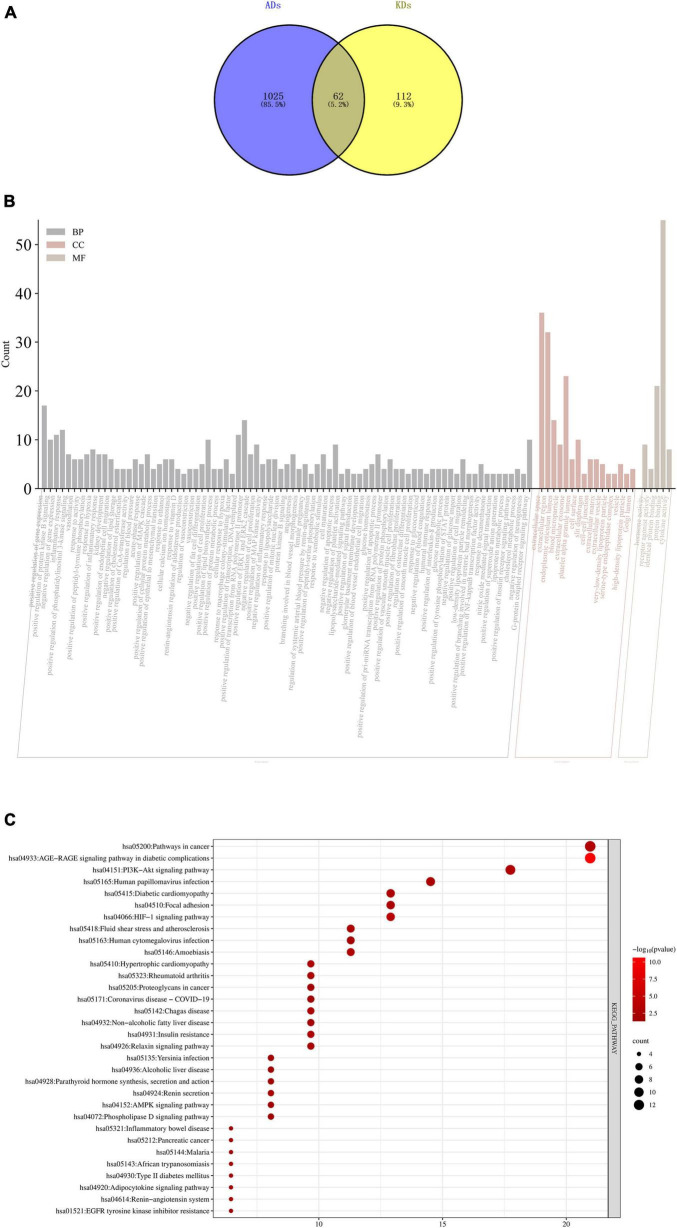
**(A)** Intersection targets of ADs and KDs; **(B)** GO enrichment analyses of intersection targets of ADs and KDs. The order from left to right is BP, CC and MF, the *X*-axis represents the function of GO, and the *Y*-axis represents the number of genes enriched by GO; **(C)** KEGG enrichment analyses of intersection targets of ADs and KDs. The *X*-axis represents the number of genes enriched by KEGG, and the *Y*-axis represents KEGG signaling pathway. The circle area indicates the number of targets, and the red color from deep to shallow indicates the enrichment degree from low to high. BP, biological process; CC, cell component; MF, molecular function.

Allergic diseases (ADs) and KDs are strongly influenced by genetic factors, it is suggested that the two diseases may have the same genetic mechanism and biological process. The results of GO and KEGG enrichment analyses indicated that they occurred and developed mainly through targets such as IL-6, ACTN4, TNF and pathways such as PI3k-Akt, AGE-RAGE signaling pathways (Figure 4).

## Discussion

Allergic diseases (ADs) and KDs are both significant public health problems that have aroused widespread concern in academic community (33, 34). However, the evidence regarding their association remains incomplete. We found that genetically predicted ADs were significantly positively associated with risk of CKD and non-specific renal failure in this study. This relationship demonstrates a unidirectional characteristic, meaning that when KDs are considered as the exposure factor, no association with ADs is found.

The connection between ADs and KDs may manifest in several aspects:

Immune system: Both diseases often trigger the production of autoantibodies within the body, which may target self-tissues or organs (35). For example, the production of anti-nuclear antibodies (ANA) and double-stranded DNA antibodies in SLE is related to disease activity and kidney involvement.

Inflammation: Inflammatory markers in the bloodstream, such as c-reactive protein (CRP) (36) and white blood cell count (37), are common immunological indicators. These markers typically rise during disease activity or exacerbation (38, 39).

Immune cells: Various types of immune cells, including T cells, B cells, monocytes and natural killer cells, play vital roles in both diseases (40, 41).

Immune complexes: Research has shown that the deposition of immune complexes can trigger inflammation and damage to glomeruli, leading to glomerulonephritis.

Cytokines: Cytokines serve as signaling molecules that facilitate interactions between cells and play critical roles in immune responses. Studies have found that tumor necrosis factor (TNF) and interleukin-6 (IL-6) are over-activated in autoimmune diseases and CKD (42).

There is no causality between ADs and other KDs, such as kidney infection and kidney stone. The reasons may be: (1) differences in participants’ age and sample size; (2) weak instrumental variables, pleiotropy, heterogeneity, linkage disequilibrium, population stratification and other factors will affect the accuracy of MR analysis. Therefore, the relationship between these diseases remains to be explored.

Traditional correlation studies, including correlation and regression analyses, are based on established parameter models for statistical analysis. These methods can only infer trends of co-variation between variables and cannot elucidate causative associations. While randomized controlled trials (RCT) are considered the gold standard for causation inference, they are often limited by ethical considerations, feasibility issues related to follow-up, and are difficult to conduct on a large scale. MR analysis effectively facilitates the translation of big data into clinical applications and offers a new perspective for gaining a deeper understanding of diseases and their prevention and treatment (25). It holds significant potential in the field of medical research.

To the best of our knowledge, this is the most comprehensive MR analysis conducted to examine the causality between ADs and KDs. The results of this study are less susceptible to confounding and reverse causation biases, providing a robust remedy for the limitations of traditional observational studies. However, there are fewer SNPs available in reverse MR analyses, the results are less reliable, and a larger sample size data set is needed for verification.

By means of GO and KEGG enrichment analyses, this study preliminarily explored that their similar mechanisms may be related to IL-6, ACTN4, TNF and PI3k-Akt, AGE-RAGE signaling pathways, which pointed out the direction for future basic research on their mechanisms.

Interleukin-6 (IL-6) is involved in regulating inflammatory reaction (43), immune reaction and other biological processes. Previous studies have found that the increase of IL-6 level is related to the severity of asthma (44), allergic rhinitis and other diseases. Therefore, IL-6 and its signaling pathway have become potential targets for the treatment of ADs (45).

Interleukin-6 (IL-6) not only participates in the initiation of inflammatory reaction, but also regulates immune response, including affecting the activation of B cells (antibody-producing cells) and antibody production. In some cases, IL-6 can help regulate the balance of immune system and reduce inflammation.

The role of IL-6 in KDs is equally complex and important, and it participates in the development of various renal pathological states (46), including nephritis, renal fibrosis (47), and CKD. IL-6 promotes the attraction and activation of inflammatory cells, the expression of fibrosis-related factors, which aggravates the structural changes and functional loss of kidney.

ACTN4 (α-actin-4) is a kind of protein found in human body, belonging to α -actin protein family, which mainly plays a role in cytoskeleton composition and intracellular signal transmission. It participates in many intracellular processes, including cell migration, cell division and signal transmission, by cross-linking actin filaments (48).

Although ACTN4 may indirectly affect the processes related to immune response, up to now, the research directly related to ACTN4 and the mechanism of ADs is not sufficient. Future research may reveal more information, especially in the cross field of cell biology and immunology.

In the kidney, ACTN4 is mainly expressed in glomerular podocytes (49). Podocyte is a key component of glomerulus, which helps to maintain the selectivity of filtration barrier and prevent macromolecular substances such as protein from leaking from blood to urine. ACTN4 helps podocytes maintain their morphology and structure, thus maintaining the filtration function of glomerulus.

Tumor necrosis factor (TNF) is a cytokine that plays an important role in human immune system, and it is very important for regulating biological processes such as inflammatory reaction (50), cell proliferation, differentiation and death. ADs are usually caused by excessive immune response in the body. This reaction leads to a large number of IgE antibodies, which in turn activates mast cells and eosinophils and releases inflammatory mediators including histamine, thus causing allergic symptoms. TNF can promote the migration of inflammatory cells, such as eosinophils and neutrophils, to the inflammatory site, aggravate allergic reactions (51), promote the production of cytokines, enhance the activity of immune cells, and lead to a stronger immune response (52).

Under the background of KDs, TNF can be over-expressed as a part of inflammation and immune response, activating inflammatory cells in the kidney, such as macrophages and T cells, leading to inflammatory reaction, aggravating the progress of the disease and affecting renal function (53).

PI3k-Akt signaling pathway is an important signal transduction pathway in cells, which is involved in regulating cell growth, division, survival, metabolism and other biological functions (54). PI3k-Akt pathway plays a key role in regulating the activation and survival of immune cells, especially T cells and B cells. During allergic reaction, the activation of these immune cells is the basis of producing specific IgE antibodies and releasing inflammatory mediators, and the activation of PI3k-Akt pathway is an important regulatory factor in this process (55). PI3k-Akt signaling pathway is involved in regulating the survival, proliferation and release of inflammatory mediators of inflammatory cells, affecting the chemotaxis and migration of immune cells to inflammatory sites, thus aggravating allergic reactions.

PI3k-Akt signaling pathway also plays an important role in KDs, involving the growth, metabolism, repair and protection mechanism of kidney (56). It may not only be a protective mechanism during injury, but also promote the pathological process when the disease progresses. Therefore, the study of this pathway is not only helpful to deeply understand the molecular mechanism of KDs, but also provides a possible target for developing new therapeutic strategies.

AGE-RAGE interaction can cause inflammation and oxidative stress, thus playing a role in the development of KDs, ADs and inflammatory diseases. Some studies show that the activation of AGE-RAGE system is related to the occurrence and development of ADs, such as asthma, allergic rhinitis and eczema. This may be because the activation of AGE-RAGE system can lead to the release of inflammatory mediators, affect the activity of immune cells and promote the occurrence of allergic inflammation. Activation of AGE-RAGE signaling pathway can also lead to the increase of intracellular oxidative stress, thus affecting the physiological function and immune regulation of cells, or promoting the destruction of mucosal barrier, thus aggravating the degree of allergic inflammation.

AGE-RAGE signaling pathway accelerates the progress of KDs (57) by regulating inflammatory reaction, fibrosis process and oxidative stress. Activation of AGE-RAGE signaling pathway can induce inflammatory reactions in renal tubules, interstitium and glomerulus (58), and increase the release of inflammatory factors, such as TNF-α and IL-6. The release of these inflammatory factors will lead to inflammatory cell infiltration and pathological changes in renal tissue. The activation of AGE-RAGE signaling pathway can also promote the fibrosis process of kidney tissue, which leads to the increase of collagen deposition and the proliferation of extracellular matrix, which leads to glomerular sclerosis, interstitial fibrosis and tubulointerstitial scar formation, thus affecting the normal structure and function of kidney.

### Limitations of the study

(1) This study primarily involves populations of European descent, and there may be racial differences limiting its generalizability.
(2) There may be variations in age and gender distribution between the populations in the two samples used in this study, which could potentially introduce bias (59).
(3) This study only covers a subset (asthma, hay fever, eczema) of allergic reactions, and future research should include large-scale and abundant data to obtain more comprehensive results.

## Conclusion

In summary, we found that genetically predicted ADs was significantly positively associated with risk of CKD and non-specific renal failure. Further exploration of this association can provide insights into the potential biological roles of immune factors in the pathogenesis of KDs. This result guides us to strengthen interdisciplinary cooperation and the monitoring of immune markers in patients with CKD in clinical practice, so as to improve the therapeutic effect and provide strong support for the long-term management and prevention of patients with CKD.

## Data availability statement

The original contributions presented in this study are included in this article/Supplementary material, further inquiries can be directed to the corresponding authors.

## Ethics statement

Ethical approval was not required for this study involving humans in accordance with the local legislation and institutional requirements. Written informed consent to participate in this study was not required from the participants or the participants’ legal guardians/next of kin in accordance with the national legislation and the institutional requirements.

## Authors contributions

ZP: Conceptualization, Data curation, Methodology, Writing – original draft. XD: Investigation, Methodology, Software, Writing – original draft. YL: Formal Analysis, Methodology, Writing – original draft. ZL: Writing – original draft. YW: Writing – original draft. WZ: Supervision, Writing – review & editing. BD: Supervision, Writing – review & editing.

## Footnotes

1
http://www.ukbiobank.ac.uk/

2
https://r9.finngen.fi/

3
www.r-project.org/

4
http://www.phenoscanner.medschl.cam.ac.uk/

5
http://www.genecards.org

6
https://bioinfogp.cnb.csic.es/tools/venny

7
david.ncifcrf.gov
