# Supplementary material for: Causality between allergic diseases and kidney diseases: a two-sample Mendelian randomization study
Source: Front Med (Lausanne). 2024 Mar 12;11:1347152. doi: 10.3389/fmed.2024.1347152 (PMC10963543; doi:10.3389/fmed.2024.1347152)
Supplement: Supplementary file 1 [file Data_Sheet_1.docx]

Supplementary Material

## Supplementary Table 1

**1.1 Mendelian randomization analysis of allergic diseases and kidney stone, ureter stone or bladder stone.**

| **Outcome:ebi-a-GCST90038631** | | | exposure | | | |  | outcome | | | |
| --- | --- | --- | --- | --- | --- | --- | --- | --- | --- | --- | --- |
| SNP | effect_allele | other_allele | beta | eaf | se | pval |  | beta | eaf | se | pval |
| rs10033073 | A | G | -4.37E-02 | 3.50E-01 | 7.20E-03 | 1.36E-09 |  | -1.40E-05 | 6.35E-01 | 1.86E-04 | 9.30E-01 |
| rs10174949 | A | G | -6.56E-02 | 3.16E-01 | 6.30E-03 | 9.92E-26 |  | -7.95E-05 | 2.87E-01 | 1.97E-04 | 6.90E-01 |
| rs10414065 | T | C | -9.17E-02 | 7.82E-02 | 1.20E-02 | 2.29E-14 |  | -7.88E-05 | 6.67E-02 | 3.65E-04 | 8.30E-01 |
| rs10519067 | A | G | -5.18E-02 | 1.40E-01 | 8.50E-03 | 1.10E-09 |  | 1.24E-04 | 1.36E-01 | 2.61E-04 | 6.40E-01 |
| rs1059513 | T | C | 8.28E-02 | 1.11E-01 | 9.40E-03 | 1.15E-18 |  | 3.04E-04 | 8.95E-01 | 2.91E-04 | 2.90E-01 |
| rs10789841 | T | C | 4.56E-02 | 3.10E-01 | 6.40E-03 | 1.48E-12 |  | 4.77E-04 | 7.36E-01 | 2.02E-04 | 1.80E-02 |
| rs10865050 | A | G | -1.25E-01 | 1.53E-01 | 8.40E-03 | 6.37E-50 |  | 1.10E-04 | 1.40E-01 | 2.57E-04 | 6.60E-01 |
| rs11033545 | T | G | -3.34E-02 | 4.42E-01 | 6.00E-03 | 2.41E-08 |  | 1.60E-04 | 5.91E-01 | 1.84E-04 | 3.90E-01 |
| rs11168245 | C | G | 4.72E-02 | 1.99E-01 | 7.00E-03 | 1.41E-11 |  | -3.33E-05 | 2.31E-01 | 2.12E-04 | 8.70E-01 |
| rs11236814 | A | T | 6.53E-02 | 9.18E-02 | 9.80E-03 | 3.25E-11 |  | -2.09E-05 | 1.04E-01 | 2.94E-04 | 9.40E-01 |
| rs12123821 | T | C | 1.16E-01 | 4.76E-02 | 1.40E-02 | 1.04E-16 |  | 7.02E-04 | 4.54E-02 | 4.28E-04 | 1.00E-01 |
| rs1214598 | A | G | -4.01E-02 | 3.32E-01 | 6.00E-03 | 2.14E-11 |  | -9.71E-05 | 3.74E-01 | 1.86E-04 | 6.00E-01 |
| rs12365699 | A | G | -5.92E-02 | 1.53E-01 | 7.80E-03 | 3.54E-14 |  | -4.54E-04 | 1.63E-01 | 2.43E-04 | 6.00E-02 |
| rs12413578 | T | C | -9.34E-02 | 9.86E-02 | 9.40E-03 | 3.31E-23 |  | 1.81E-04 | 1.03E-01 | 2.93E-04 | 5.40E-01 |
| rs12440045 | A | C | -4.03E-02 | 4.52E-01 | 5.90E-03 | 7.68E-12 |  | 4.24E-04 | 4.58E-01 | 1.82E-04 | 2.00E-02 |
| rs12551834 | A | G | -6.15E-02 | 7.99E-02 | 1.03E-02 | 2.72E-09 |  | -7.75E-04 | 9.93E-02 | 2.99E-04 | 9.50E-03 |
| rs12625547 | T | G | 4.32E-02 | 2.14E-01 | 7.70E-03 | 1.98E-08 |  | 4.34E-05 | 8.23E-01 | 2.35E-04 | 8.60E-01 |
| rs1289273 | A | G | -3.50E-02 | 4.27E-01 | 5.70E-03 | 1.09E-09 |  | 1.58E-04 | 4.83E-01 | 1.79E-04 | 3.80E-01 |
| rs12941864 | T | C | -3.35E-02 | 4.66E-01 | 6.10E-03 | 4.98E-08 |  | 2.12E-04 | 5.22E-01 | 1.80E-04 | 2.40E-01 |
| rs1419675 | T | G | -3.85E-02 | 2.42E-01 | 7.00E-03 | 4.72E-08 |  | -2.82E-04 | 7.41E-01 | 2.03E-04 | 1.70E-01 |
| rs144829310 | T | G | 8.28E-02 | 1.57E-01 | 7.80E-03 | 2.60E-26 |  | 1.72E-04 | 1.60E-01 | 2.44E-04 | 4.80E-01 |
| rs1689510 | C | G | 5.10E-02 | 3.16E-01 | 6.10E-03 | 3.39E-17 |  | -1.03E-04 | 3.31E-01 | 1.90E-04 | 5.70E-01 |
| rs16903574 | C | G | -7.00E-02 | 8.16E-02 | 1.10E-02 | 1.68E-10 |  | 8.16E-05 | 7.32E-02 | 3.50E-04 | 8.10E-01 |
| rs17664743 | A | G | 4.10E-02 | 2.04E-01 | 7.10E-03 | 8.27E-09 |  | 4.27E-05 | 2.11E-01 | 2.20E-04 | 8.50E-01 |
| rs1885013 | A | G | -3.82E-02 | 3.59E-01 | 6.30E-03 | 1.33E-09 |  | 1.47E-04 | 7.08E-01 | 1.99E-04 | 4.50E-01 |
| rs2134814 | C | G | 4.27E-02 | 3.54E-01 | 6.00E-03 | 1.03E-12 |  | 6.08E-05 | 3.45E-01 | 1.88E-04 | 7.40E-01 |
| rs2212434 | T | C | 8.69E-02 | 4.29E-01 | 5.70E-03 | 8.93E-52 |  | 2.96E-05 | 4.45E-01 | 1.80E-04 | 8.70E-01 |
| rs2221641 | T | C | -4.19E-02 | 3.88E-01 | 5.90E-03 | 1.03E-12 |  | -6.11E-05 | 6.08E-01 | 1.83E-04 | 7.40E-01 |
| rs2241099 | C | G | 6.97E-02 | 2.62E-01 | 6.60E-03 | 4.62E-26 |  | 3.84E-04 | 2.47E-01 | 2.07E-04 | 6.40E-02 |
| rs228619 | A | G | -3.62E-02 | 4.69E-01 | 5.70E-03 | 2.41E-10 |  | -1.70E-04 | 5.00E-01 | 1.78E-04 | 3.40E-01 |
| rs2477923 | T | C | 3.32E-02 | 4.98E-01 | 5.70E-03 | 7.23E-09 |  | -1.90E-04 | 5.30E-01 | 1.79E-04 | 2.90E-01 |
| rs249677 | A | C | 3.66E-02 | 3.61E-01 | 5.90E-03 | 7.54E-10 |  | 1.49E-04 | 6.35E-01 | 1.86E-04 | 4.20E-01 |
| rs2766678 | A | G | -5.59E-02 | 2.13E-01 | 7.40E-03 | 2.95E-14 |  | -1.80E-04 | 7.94E-01 | 2.27E-04 | 4.20E-01 |
| rs2854001 | A | G | 5.37E-02 | 1.84E-01 | 7.10E-03 | 3.31E-14 |  | 6.63E-05 | 2.24E-01 | 2.14E-04 | 7.50E-01 |
| rs2910162 | A | G | -3.44E-02 | 3.16E-01 | 6.10E-03 | 1.54E-08 |  | -5.12E-04 | 3.40E-01 | 1.91E-04 | 7.50E-03 |
| rs3024665 | T | C | 6.65E-02 | 7.65E-02 | 1.19E-02 | 2.18E-08 |  | 1.25E-05 | 9.29E-01 | 3.64E-04 | 9.80E-01 |
| rs3128959 | A | G | -6.01E-02 | 1.24E-01 | 9.50E-03 | 2.25E-10 |  | -8.18E-05 | 1.19E-01 | 2.79E-04 | 7.70E-01 |
| rs34290285 | A | G | -7.66E-02 | 2.65E-01 | 7.50E-03 | 1.16E-24 |  | -2.28E-04 | 2.57E-01 | 2.04E-04 | 2.60E-01 |
| rs3540 | A | G | -3.46E-02 | 3.45E-01 | 6.10E-03 | 1.28E-08 |  | 1.50E-04 | 3.31E-01 | 1.91E-04 | 4.30E-01 |
| rs4296977 | T | C | -5.34E-02 | 1.58E-01 | 8.20E-03 | 6.92E-11 |  | 3.98E-04 | 8.62E-01 | 2.60E-04 | 1.20E-01 |
| rs479844 | A | G | -4.12E-02 | 4.29E-01 | 5.80E-03 | 1.15E-12 |  | -8.41E-05 | 4.53E-01 | 1.80E-04 | 6.40E-01 |
| rs4943794 | C | G | 3.96E-02 | 2.26E-01 | 7.00E-03 | 1.41E-08 |  | -7.06E-05 | 2.09E-01 | 2.19E-04 | 7.40E-01 |
| rs4973380 | A | T | 3.97E-02 | 2.43E-01 | 6.70E-03 | 2.59E-09 |  | -2.53E-05 | 2.46E-01 | 2.07E-04 | 9.00E-01 |
| rs519973 | A | G | 3.58E-02 | 3.54E-01 | 6.00E-03 | 3.23E-09 |  | 4.11E-04 | 3.39E-01 | 1.88E-04 | 2.90E-02 |
| rs56375023 | A | G | 7.31E-02 | 2.13E-01 | 6.80E-03 | 3.11E-27 |  | 7.46E-05 | 2.30E-01 | 2.12E-04 | 7.20E-01 |
| rs5743618 | A | C | -9.15E-02 | 2.99E-01 | 6.70E-03 | 2.73E-42 |  | -5.74E-05 | 2.63E-01 | 2.17E-04 | 8.00E-01 |
| rs5758343 | A | T | 4.74E-02 | 2.18E-01 | 7.10E-03 | 2.17E-11 |  | 6.44E-04 | 2.02E-01 | 2.23E-04 | 3.80E-03 |
| rs58939053 | T | C | 5.44E-02 | 3.44E-01 | 6.10E-03 | 3.85E-19 |  | 7.30E-05 | 3.24E-01 | 1.91E-04 | 7.00E-01 |
| rs6011033 | A | G | -4.37E-02 | 2.25E-01 | 6.90E-03 | 2.66E-10 |  | 3.04E-04 | 2.34E-01 | 2.13E-04 | 1.50E-01 |
| rs61192126 | T | C | 3.84E-02 | 3.10E-01 | 6.40E-03 | 1.56E-09 |  | -7.74E-05 | 7.06E-01 | 1.97E-04 | 6.90E-01 |
| rs61816766 | T | C | -1.42E-01 | 2.04E-02 | 1.76E-02 | 6.76E-16 |  | -7.39E-04 | 9.69E-01 | 5.28E-04 | 1.60E-01 |
| rs62626322 | T | G | -6.74E-02 | 9.69E-02 | 9.10E-03 | 1.35E-13 |  | 1.45E-04 | 8.87E-01 | 2.82E-04 | 6.10E-01 |
| rs6461503 | T | C | 4.10E-02 | 4.73E-01 | 5.70E-03 | 8.81E-13 |  | -1.65E-04 | 4.83E-01 | 1.79E-04 | 3.50E-01 |
| rs6489785 | T | C | 4.28E-02 | 3.69E-01 | 5.90E-03 | 3.57E-13 |  | -2.11E-04 | 3.80E-01 | 1.84E-04 | 2.50E-01 |
| rs6594499 | A | C | -7.23E-02 | 4.81E-01 | 5.70E-03 | 1.10E-36 |  | 1.97E-04 | 4.95E-01 | 1.80E-04 | 2.70E-01 |
| rs6800001 | A | G | 3.30E-02 | 3.42E-01 | 5.90E-03 | 2.28E-08 |  | -1.37E-04 | 3.71E-01 | 1.86E-04 | 4.50E-01 |
| rs6881706 | T | G | -7.07E-02 | 2.93E-01 | 6.40E-03 | 1.73E-28 |  | 4.44E-05 | 2.71E-01 | 2.00E-04 | 8.30E-01 |
| rs6990534 | A | G | 4.00E-02 | 3.57E-01 | 6.30E-03 | 1.74E-10 |  | 6.52E-05 | 2.95E-01 | 1.95E-04 | 7.40E-01 |
| rs7224129 | A | G | 5.35E-02 | 4.73E-01 | 5.70E-03 | 8.11E-21 |  | 4.70E-05 | 4.86E-01 | 1.78E-04 | 8.00E-01 |
| rs72774901 | A | T | 1.12E-01 | 8.16E-02 | 1.08E-02 | 3.92E-25 |  | 1.45E-04 | 7.23E-02 | 3.46E-04 | 6.80E-01 |
| rs7406234 | T | C | 3.37E-02 | 4.35E-01 | 5.70E-03 | 4.30E-09 |  | -1.38E-05 | 4.43E-01 | 1.81E-04 | 9.40E-01 |
| rs74847330 | A | G | 4.97E-02 | 1.22E-01 | 8.80E-03 | 1.67E-08 |  | -3.66E-04 | 8.76E-01 | 2.70E-04 | 1.80E-01 |
| rs7521390 | A | C | -3.58E-02 | 2.84E-01 | 6.30E-03 | 1.40E-08 |  | -1.37E-04 | 7.01E-01 | 1.98E-04 | 4.90E-01 |
| rs7625643 | A | G | -3.46E-02 | 4.42E-01 | 5.80E-03 | 2.96E-09 |  | -1.28E-04 | 5.53E-01 | 1.82E-04 | 4.90E-01 |
| rs7712601 | T | C | 3.46E-02 | 4.18E-01 | 5.80E-03 | 3.28E-09 |  | 4.45E-04 | 3.90E-01 | 1.83E-04 | 1.50E-02 |
| rs80064395 | T | C | -7.26E-02 | 5.78E-02 | 1.07E-02 | 1.32E-11 |  | 1.87E-04 | 8.23E-02 | 3.27E-04 | 5.70E-01 |
| rs8030821 | A | T | -3.37E-02 | 3.90E-01 | 6.00E-03 | 1.54E-08 |  | -1.16E-04 | 3.90E-01 | 1.84E-04 | 5.30E-01 |
| rs848 | A | C | 6.01E-02 | 2.36E-01 | 7.30E-03 | 1.62E-16 |  | 8.04E-05 | 1.93E-01 | 2.27E-04 | 7.30E-01 |
| rs9372120 | T | G | -4.06E-02 | 1.75E-01 | 7.10E-03 | 1.02E-08 |  | 6.91E-05 | 7.97E-01 | 2.22E-04 | 7.60E-01 |
| rs9877752 | A | G | 3.98E-02 | 4.12E-01 | 5.80E-03 | 4.98E-12 |  | -2.12E-04 | 4.43E-01 | 1.80E-04 | 2.40E-01 |
| Abbreviation: SNP, single nucleotide polymorphism; EAF, effect allele frequency; SE, standard error.  **1.2 Mendelian randomization analysis of allergic diseases and other renal or kidney problem.** | | | | | | | | | | | |
| **Outcome:ebi-a-GCST90038666** | | | exposure | | | |  | outcome | | | |
| SNP | effect_allele | other_allele | beta | eaf | se | pval |  | beta | eaf | se | pval |
| rs10033073 | A | G | -4.37E-02 | 3.50E-01 | 7.20E-03 | 1.36E-09 |  | 2.32E-04 | 6.35E-01 | 1.56E-04 | 1.40E-01 |
| rs10174949 | A | G | -6.56E-02 | 3.16E-01 | 6.30E-03 | 9.92E-26 |  | -1.83E-04 | 2.87E-01 | 1.66E-04 | 2.70E-01 |
| rs10414065 | T | C | -9.17E-02 | 7.82E-02 | 1.20E-02 | 2.29E-14 |  | 1.54E-04 | 6.67E-02 | 3.06E-04 | 6.20E-01 |
| rs10519067 | A | G | -5.18E-02 | 1.40E-01 | 8.50E-03 | 1.10E-09 |  | -2.87E-04 | 1.36E-01 | 2.19E-04 | 1.90E-01 |
| rs1059513 | T | C | 8.28E-02 | 1.11E-01 | 9.40E-03 | 1.15E-18 |  | 2.80E-05 | 8.95E-01 | 2.44E-04 | 9.10E-01 |
| rs10789841 | T | C | 4.56E-02 | 3.10E-01 | 6.40E-03 | 1.48E-12 |  | 2.13E-04 | 7.36E-01 | 1.70E-04 | 2.10E-01 |
| rs10865050 | A | G | -1.25E-01 | 1.53E-01 | 8.40E-03 | 6.37E-50 |  | -8.19E-05 | 1.40E-01 | 2.16E-04 | 7.00E-01 |
| rs11033545 | T | G | -3.34E-02 | 4.42E-01 | 6.00E-03 | 2.41E-08 |  | 1.65E-04 | 5.91E-01 | 1.55E-04 | 2.90E-01 |
| rs11168245 | C | G | 4.72E-02 | 1.99E-01 | 7.00E-03 | 1.41E-11 |  | 8.99E-05 | 2.31E-01 | 1.78E-04 | 6.10E-01 |
| rs11236814 | A | T | 6.53E-02 | 9.18E-02 | 9.80E-03 | 3.25E-11 |  | 1.96E-04 | 1.04E-01 | 2.46E-04 | 4.30E-01 |
| rs12123821 | T | C | 1.16E-01 | 4.76E-02 | 1.40E-02 | 1.04E-16 |  | -1.51E-05 | 4.54E-02 | 3.59E-04 | 9.70E-01 |
| rs1214598 | A | G | -4.01E-02 | 3.32E-01 | 6.00E-03 | 2.14E-11 |  | 1.19E-04 | 3.74E-01 | 1.56E-04 | 4.50E-01 |
| rs12365699 | A | G | -5.92E-02 | 1.53E-01 | 7.80E-03 | 3.54E-14 |  | -8.55E-05 | 1.63E-01 | 2.04E-04 | 6.70E-01 |
| rs12413578 | T | C | -9.34E-02 | 9.86E-02 | 9.40E-03 | 3.31E-23 |  | 1.76E-04 | 1.03E-01 | 2.46E-04 | 4.80E-01 |
| rs12440045 | A | C | -4.03E-02 | 4.52E-01 | 5.90E-03 | 7.68E-12 |  | -9.80E-05 | 4.58E-01 | 1.53E-04 | 5.20E-01 |
| rs12551834 | A | G | -6.15E-02 | 7.99E-02 | 1.03E-02 | 2.72E-09 |  | -2.28E-04 | 9.93E-02 | 2.51E-04 | 3.60E-01 |
| rs12625547 | T | G | 4.32E-02 | 2.14E-01 | 7.70E-03 | 1.98E-08 |  | -2.66E-04 | 8.23E-01 | 1.97E-04 | 1.80E-01 |
| rs1289273 | A | G | -3.50E-02 | 4.27E-01 | 5.70E-03 | 1.09E-09 |  | -3.32E-04 | 4.83E-01 | 1.50E-04 | 2.70E-02 |
| rs12941864 | T | C | -3.35E-02 | 4.66E-01 | 6.10E-03 | 4.98E-08 |  | -3.68E-04 | 5.22E-01 | 1.51E-04 | 1.50E-02 |
| rs1419675 | T | G | -3.85E-02 | 2.42E-01 | 7.00E-03 | 4.72E-08 |  | 3.49E-04 | 7.41E-01 | 1.70E-04 | 4.00E-02 |
| rs144829310 | T | G | 8.28E-02 | 1.57E-01 | 7.80E-03 | 2.60E-26 |  | -3.11E-04 | 1.60E-01 | 2.04E-04 | 1.30E-01 |
| rs1689510 | C | G | 5.10E-02 | 3.16E-01 | 6.10E-03 | 3.39E-17 |  | -1.24E-04 | 3.31E-01 | 1.59E-04 | 4.30E-01 |
| rs16903574 | C | G | -7.00E-02 | 8.16E-02 | 1.10E-02 | 1.68E-10 |  | -1.99E-04 | 7.32E-02 | 2.94E-04 | 5.00E-01 |
| rs17664743 | A | G | 4.10E-02 | 2.04E-01 | 7.10E-03 | 8.27E-09 |  | -5.60E-05 | 2.11E-01 | 1.84E-04 | 7.60E-01 |
| rs1885013 | A | G | -3.82E-02 | 3.59E-01 | 6.30E-03 | 1.33E-09 |  | -9.55E-05 | 7.08E-01 | 1.67E-04 | 5.70E-01 |
| rs2134814 | C | G | 4.27E-02 | 3.54E-01 | 6.00E-03 | 1.03E-12 |  | -3.12E-05 | 3.45E-01 | 1.57E-04 | 8.40E-01 |
| rs2212434 | T | C | 8.69E-02 | 4.29E-01 | 5.70E-03 | 8.93E-52 |  | 1.79E-04 | 4.45E-01 | 1.51E-04 | 2.40E-01 |
| rs2221641 | T | C | -4.19E-02 | 3.88E-01 | 5.90E-03 | 1.03E-12 |  | -4.81E-05 | 6.08E-01 | 1.54E-04 | 7.50E-01 |
| rs2241099 | C | G | 6.97E-02 | 2.62E-01 | 6.60E-03 | 4.62E-26 |  | -8.18E-05 | 2.47E-01 | 1.73E-04 | 6.40E-01 |
| rs228619 | A | G | -3.62E-02 | 4.69E-01 | 5.70E-03 | 2.41E-10 |  | 4.44E-05 | 5.00E-01 | 1.49E-04 | 7.70E-01 |
| rs2477923 | T | C | 3.32E-02 | 4.98E-01 | 5.70E-03 | 7.23E-09 |  | -5.82E-05 | 5.30E-01 | 1.50E-04 | 7.00E-01 |
| rs249677 | A | C | 3.66E-02 | 3.61E-01 | 5.90E-03 | 7.54E-10 |  | -2.12E-04 | 6.35E-01 | 1.56E-04 | 1.70E-01 |
| rs2766678 | A | G | -5.59E-02 | 2.13E-01 | 7.40E-03 | 2.95E-14 |  | -2.99E-06 | 7.94E-01 | 1.90E-04 | 9.90E-01 |
| rs2854001 | A | G | 5.37E-02 | 1.84E-01 | 7.10E-03 | 3.31E-14 |  | 5.29E-05 | 2.24E-01 | 1.79E-04 | 7.70E-01 |
| rs2910162 | A | G | -3.44E-02 | 3.16E-01 | 6.10E-03 | 1.54E-08 |  | -6.14E-05 | 3.40E-01 | 1.61E-04 | 7.00E-01 |
| rs3024665 | T | C | 6.65E-02 | 7.65E-02 | 1.19E-02 | 2.18E-08 |  | 2.39E-04 | 9.29E-01 | 3.05E-04 | 4.30E-01 |
| rs3128959 | A | G | -6.01E-02 | 1.24E-01 | 9.50E-03 | 2.25E-10 |  | -3.05E-04 | 1.19E-01 | 2.34E-04 | 1.90E-01 |
| rs34004019 | A | G | 9.13E-02 | 3.04E-01 | 7.10E-03 | 2.52E-38 |  | -1.18E-04 | 7.88E-01 | 1.96E-04 | 5.50E-01 |
| rs34290285 | A | G | -7.66E-02 | 2.65E-01 | 7.50E-03 | 1.16E-24 |  | -3.60E-04 | 2.57E-01 | 1.71E-04 | 3.50E-02 |
| rs3540 | A | G | -3.46E-02 | 3.45E-01 | 6.10E-03 | 1.28E-08 |  | 9.01E-05 | 3.31E-01 | 1.60E-04 | 5.70E-01 |
| rs4296977 | T | C | -5.34E-02 | 1.58E-01 | 8.20E-03 | 6.92E-11 |  | -2.14E-04 | 8.62E-01 | 2.18E-04 | 3.30E-01 |
| rs479844 | A | G | -4.12E-02 | 4.29E-01 | 5.80E-03 | 1.15E-12 |  | -9.87E-05 | 4.53E-01 | 1.51E-04 | 5.10E-01 |
| rs4943794 | C | G | 3.96E-02 | 2.26E-01 | 7.00E-03 | 1.41E-08 |  | -3.69E-05 | 2.09E-01 | 1.84E-04 | 8.40E-01 |
| rs4973380 | A | T | 3.97E-02 | 2.43E-01 | 6.70E-03 | 2.59E-09 |  | 3.42E-05 | 2.46E-01 | 1.74E-04 | 8.40E-01 |
| rs519973 | A | G | 3.58E-02 | 3.54E-01 | 6.00E-03 | 3.23E-09 |  | 5.03E-05 | 3.39E-01 | 1.58E-04 | 7.50E-01 |
| rs56375023 | A | G | 7.31E-02 | 2.13E-01 | 6.80E-03 | 3.11E-27 |  | 3.36E-04 | 2.30E-01 | 1.78E-04 | 5.90E-02 |
| rs5743618 | A | C | -9.15E-02 | 2.99E-01 | 6.70E-03 | 2.73E-42 |  | -1.76E-04 | 2.63E-01 | 1.82E-04 | 3.30E-01 |
| rs5758343 | A | T | 4.74E-02 | 2.18E-01 | 7.10E-03 | 2.17E-11 |  | -5.57E-05 | 2.02E-01 | 1.87E-04 | 7.70E-01 |
| rs58939053 | T | C | 5.44E-02 | 3.44E-01 | 6.10E-03 | 3.85E-19 |  | -1.95E-04 | 3.24E-01 | 1.60E-04 | 2.20E-01 |
| rs6011033 | A | G | -4.37E-02 | 2.25E-01 | 6.90E-03 | 2.66E-10 |  | -2.94E-04 | 2.34E-01 | 1.78E-04 | 9.90E-02 |
| rs61192126 | T | C | 3.84E-02 | 3.10E-01 | 6.40E-03 | 1.56E-09 |  | 8.73E-05 | 7.06E-01 | 1.65E-04 | 6.00E-01 |
| rs61816766 | T | C | -1.42E-01 | 2.04E-02 | 1.76E-02 | 6.76E-16 |  | 3.45E-04 | 9.69E-01 | 4.43E-04 | 4.30E-01 |
| rs62626322 | T | G | -6.74E-02 | 9.69E-02 | 9.10E-03 | 1.35E-13 |  | 8.74E-06 | 8.87E-01 | 2.37E-04 | 9.70E-01 |
| rs6461503 | T | C | 4.10E-02 | 4.73E-01 | 5.70E-03 | 8.81E-13 |  | 3.64E-04 | 4.83E-01 | 1.50E-04 | 1.50E-02 |
| rs6489785 | T | C | 4.28E-02 | 3.69E-01 | 5.90E-03 | 3.57E-13 |  | 9.04E-05 | 3.80E-01 | 1.55E-04 | 5.60E-01 |
| rs6594499 | A | C | -7.23E-02 | 4.81E-01 | 5.70E-03 | 1.10E-36 |  | 1.45E-04 | 4.95E-01 | 1.51E-04 | 3.30E-01 |
| rs6800001 | A | G | 3.30E-02 | 3.42E-01 | 5.90E-03 | 2.28E-08 |  | 2.57E-05 | 3.71E-01 | 1.56E-04 | 8.70E-01 |
| rs6881706 | T | G | -7.07E-02 | 2.93E-01 | 6.40E-03 | 1.73E-28 |  | 1.17E-04 | 2.71E-01 | 1.68E-04 | 4.90E-01 |
| rs6990534 | A | G | 4.00E-02 | 3.57E-01 | 6.30E-03 | 1.74E-10 |  | -8.71E-05 | 2.95E-01 | 1.64E-04 | 5.90E-01 |
| rs7224129 | A | G | 5.35E-02 | 4.73E-01 | 5.70E-03 | 8.11E-21 |  | -5.74E-05 | 4.86E-01 | 1.50E-04 | 7.00E-01 |
| rs72774901 | A | T | 1.12E-01 | 8.16E-02 | 1.08E-02 | 3.92E-25 |  | 1.71E-04 | 7.23E-02 | 2.90E-04 | 5.50E-01 |
| rs7406234 | T | C | 3.37E-02 | 4.35E-01 | 5.70E-03 | 4.30E-09 |  | -5.46E-05 | 4.43E-01 | 1.51E-04 | 7.20E-01 |
| rs74847330 | A | G | 4.97E-02 | 1.22E-01 | 8.80E-03 | 1.67E-08 |  | -1.28E-04 | 8.76E-01 | 2.27E-04 | 5.70E-01 |
| rs7521390 | A | C | -3.58E-02 | 2.84E-01 | 6.30E-03 | 1.40E-08 |  | 7.41E-05 | 7.01E-01 | 1.66E-04 | 6.60E-01 |
| rs7625643 | A | G | -3.46E-02 | 4.42E-01 | 5.80E-03 | 2.96E-09 |  | 2.41E-04 | 5.53E-01 | 1.53E-04 | 1.20E-01 |
| rs7712601 | T | C | 3.46E-02 | 4.18E-01 | 5.80E-03 | 3.28E-09 |  | -3.32E-04 | 3.90E-01 | 1.54E-04 | 3.10E-02 |
| rs80064395 | T | C | -7.26E-02 | 5.78E-02 | 1.07E-02 | 1.32E-11 |  | 2.21E-04 | 8.23E-02 | 2.74E-04 | 4.20E-01 |
| rs8030821 | A | T | -3.37E-02 | 3.90E-01 | 6.00E-03 | 1.54E-08 |  | -2.57E-04 | 3.90E-01 | 1.55E-04 | 9.70E-02 |
| rs848 | A | C | 6.01E-02 | 2.36E-01 | 7.30E-03 | 1.62E-16 |  | -2.14E-04 | 1.93E-01 | 1.91E-04 | 2.60E-01 |
| rs9372120 | T | G | -4.06E-02 | 1.75E-01 | 7.10E-03 | 1.02E-08 |  | -1.24E-04 | 7.97E-01 | 1.86E-04 | 5.10E-01 |
| rs9877752 | A | G | 3.98E-02 | 4.12E-01 | 5.80E-03 | 4.98E-12 |  | 1.85E-04 | 4.43E-01 | 1.51E-04 | 2.20E-01 |
| Abbreviation: SNP, single nucleotide polymorphism; EAF, effect allele frequency; SE, standard error.  **1.3 Mendelian randomization analysis of allergic diseases and urinary tract or kidney infection.** | | | | | | | | | | | |
| **Outcome:ebi-a-GCST90038630** | | | exposure | | | |  | outcome | | | |
| SNP | effect_allele | other_allele | beta | eaf | se | pval |  | beta | eaf | se | pval |
| rs10033073 | A | G | -4.37E-02 | 3.50E-01 | 7.20E-03 | 1.36E-09 |  | 1.77E-04 | 6.35E-01 | 1.58E-04 | 2.60E-01 |
| rs10174949 | A | G | -6.56E-02 | 3.16E-01 | 6.30E-03 | 9.92E-26 |  | 1.10E-05 | 2.87E-01 | 1.68E-04 | 9.50E-01 |
| rs10414065 | T | C | -9.17E-02 | 7.82E-02 | 1.20E-02 | 2.29E-14 |  | 1.27E-04 | 6.67E-02 | 3.11E-04 | 6.80E-01 |
| rs10519067 | A | G | -5.18E-02 | 1.40E-01 | 8.50E-03 | 1.10E-09 |  | -9.95E-05 | 1.36E-01 | 2.22E-04 | 6.50E-01 |
| rs1059513 | T | C | 8.28E-02 | 1.11E-01 | 9.40E-03 | 1.15E-18 |  | -1.07E-04 | 8.95E-01 | 2.47E-04 | 6.60E-01 |
| rs10789841 | T | C | 4.56E-02 | 3.10E-01 | 6.40E-03 | 1.48E-12 |  | 1.44E-04 | 7.36E-01 | 1.72E-04 | 4.00E-01 |
| rs10865050 | A | G | -1.25E-01 | 1.53E-01 | 8.40E-03 | 6.37E-50 |  | -1.07E-05 | 1.40E-01 | 2.19E-04 | 9.60E-01 |
| rs11033545 | T | G | -3.34E-02 | 4.42E-01 | 6.00E-03 | 2.41E-08 |  | 1.91E-04 | 5.91E-01 | 1.57E-04 | 2.20E-01 |
| rs11168245 | C | G | 4.72E-02 | 1.99E-01 | 7.00E-03 | 1.41E-11 |  | -9.24E-05 | 2.31E-01 | 1.81E-04 | 6.10E-01 |
| rs11236814 | A | T | 6.53E-02 | 9.18E-02 | 9.80E-03 | 3.25E-11 |  | 2.00E-04 | 1.04E-01 | 2.50E-04 | 4.20E-01 |
| rs12123821 | T | C | 1.16E-01 | 4.76E-02 | 1.40E-02 | 1.04E-16 |  | -1.58E-04 | 4.54E-02 | 3.64E-04 | 6.60E-01 |
| rs1214598 | A | G | -4.01E-02 | 3.32E-01 | 6.00E-03 | 2.14E-11 |  | 1.49E-04 | 3.74E-01 | 1.58E-04 | 3.40E-01 |
| rs12365699 | A | G | -5.92E-02 | 1.53E-01 | 7.80E-03 | 3.54E-14 |  | 4.85E-05 | 1.63E-01 | 2.07E-04 | 8.10E-01 |
| rs12413578 | T | C | -9.34E-02 | 9.86E-02 | 9.40E-03 | 3.31E-23 |  | -1.35E-04 | 1.03E-01 | 2.50E-04 | 5.90E-01 |
| rs12440045 | A | C | -4.03E-02 | 4.52E-01 | 5.90E-03 | 7.68E-12 |  | -2.62E-04 | 4.58E-01 | 1.55E-04 | 9.10E-02 |
| rs12551834 | A | G | -6.15E-02 | 7.99E-02 | 1.03E-02 | 2.72E-09 |  | -2.09E-04 | 9.93E-02 | 2.54E-04 | 4.10E-01 |
| rs12625547 | T | G | 4.32E-02 | 2.14E-01 | 7.70E-03 | 1.98E-08 |  | 3.49E-04 | 8.23E-01 | 2.00E-04 | 8.10E-02 |
| rs1289273 | A | G | -3.50E-02 | 4.27E-01 | 5.70E-03 | 1.09E-09 |  | -1.04E-04 | 4.83E-01 | 1.53E-04 | 5.00E-01 |
| rs12941864 | T | C | -3.35E-02 | 4.66E-01 | 6.10E-03 | 4.98E-08 |  | -2.80E-05 | 5.22E-01 | 1.54E-04 | 8.60E-01 |
| rs1419675 | T | G | -3.85E-02 | 2.42E-01 | 7.00E-03 | 4.72E-08 |  | -1.04E-04 | 7.41E-01 | 1.73E-04 | 5.50E-01 |
| rs144829310 | T | G | 8.28E-02 | 1.57E-01 | 7.80E-03 | 2.60E-26 |  | -3.47E-04 | 1.60E-01 | 2.08E-04 | 9.40E-02 |
| rs1689510 | C | G | 5.10E-02 | 3.16E-01 | 6.10E-03 | 3.39E-17 |  | -2.42E-04 | 3.31E-01 | 1.62E-04 | 1.40E-01 |
| rs16903574 | C | G | -7.00E-02 | 8.16E-02 | 1.10E-02 | 1.68E-10 |  | -2.77E-04 | 7.32E-02 | 2.98E-04 | 3.50E-01 |
| rs17664743 | A | G | 4.10E-02 | 2.04E-01 | 7.10E-03 | 8.27E-09 |  | -5.22E-05 | 2.11E-01 | 1.87E-04 | 7.80E-01 |
| rs1885013 | A | G | -3.82E-02 | 3.59E-01 | 6.30E-03 | 1.33E-09 |  | -5.57E-05 | 7.08E-01 | 1.69E-04 | 7.40E-01 |
| rs2134814 | C | G | 4.27E-02 | 3.54E-01 | 6.00E-03 | 1.03E-12 |  | -6.93E-05 | 3.45E-01 | 1.60E-04 | 6.60E-01 |
| rs2212434 | T | C | 8.69E-02 | 4.29E-01 | 5.70E-03 | 8.93E-52 |  | -2.56E-04 | 4.45E-01 | 1.53E-04 | 9.40E-02 |
| rs2221641 | T | C | -4.19E-02 | 3.88E-01 | 5.90E-03 | 1.03E-12 |  | -2.95E-04 | 6.08E-01 | 1.56E-04 | 5.90E-02 |
| rs2241099 | C | G | 6.97E-02 | 2.62E-01 | 6.60E-03 | 4.62E-26 |  | 1.56E-04 | 2.47E-01 | 1.76E-04 | 3.80E-01 |
| rs228619 | A | G | -3.62E-02 | 4.69E-01 | 5.70E-03 | 2.41E-10 |  | 3.81E-05 | 5.00E-01 | 1.52E-04 | 8.00E-01 |
| rs2477923 | T | C | 3.32E-02 | 4.98E-01 | 5.70E-03 | 7.23E-09 |  | 5.56E-06 | 5.30E-01 | 1.53E-04 | 9.70E-01 |
| rs249677 | A | C | 3.66E-02 | 3.61E-01 | 5.90E-03 | 7.54E-10 |  | 1.01E-05 | 6.35E-01 | 1.58E-04 | 9.50E-01 |
| rs2766678 | A | G | -5.59E-02 | 2.13E-01 | 7.40E-03 | 2.95E-14 |  | 3.66E-05 | 7.94E-01 | 1.93E-04 | 8.50E-01 |
| rs2854001 | A | G | 5.37E-02 | 1.84E-01 | 7.10E-03 | 3.31E-14 |  | 1.09E-04 | 2.24E-01 | 1.82E-04 | 5.50E-01 |
| rs2910162 | A | G | -3.44E-02 | 3.16E-01 | 6.10E-03 | 1.54E-08 |  | 6.92E-05 | 3.40E-01 | 1.63E-04 | 6.70E-01 |
| rs3024665 | T | C | 6.65E-02 | 7.65E-02 | 1.19E-02 | 2.18E-08 |  | 7.75E-05 | 9.29E-01 | 3.10E-04 | 8.00E-01 |
| rs3128959 | A | G | -6.01E-02 | 1.24E-01 | 9.50E-03 | 2.25E-10 |  | -3.02E-05 | 1.19E-01 | 2.37E-04 | 9.00E-01 |
| rs34004019 | A | G | 9.13E-02 | 3.04E-01 | 7.10E-03 | 2.52E-38 |  | 5.93E-05 | 7.88E-01 | 1.99E-04 | 7.70E-01 |
| rs34290285 | A | G | -7.66E-02 | 2.65E-01 | 7.50E-03 | 1.16E-24 |  | 8.41E-05 | 2.57E-01 | 1.74E-04 | 6.30E-01 |
| rs3540 | A | G | -3.46E-02 | 3.45E-01 | 6.10E-03 | 1.28E-08 |  | 1.18E-04 | 3.31E-01 | 1.63E-04 | 4.70E-01 |
| rs4296977 | T | C | -5.34E-02 | 1.58E-01 | 8.20E-03 | 6.92E-11 |  | 6.89E-05 | 8.62E-01 | 2.21E-04 | 7.60E-01 |
| rs479844 | A | G | -4.12E-02 | 4.29E-01 | 5.80E-03 | 1.15E-12 |  | 6.95E-05 | 4.53E-01 | 1.53E-04 | 6.50E-01 |
| rs4943794 | C | G | 3.96E-02 | 2.26E-01 | 7.00E-03 | 1.41E-08 |  | 2.45E-04 | 2.09E-01 | 1.87E-04 | 1.90E-01 |
| rs4973380 | A | T | 3.97E-02 | 2.43E-01 | 6.70E-03 | 2.59E-09 |  | -1.11E-04 | 2.46E-01 | 1.76E-04 | 5.30E-01 |
| rs519973 | A | G | 3.58E-02 | 3.54E-01 | 6.00E-03 | 3.23E-09 |  | 2.70E-04 | 3.39E-01 | 1.60E-04 | 9.10E-02 |
| rs56375023 | A | G | 7.31E-02 | 2.13E-01 | 6.80E-03 | 3.11E-27 |  | -7.55E-06 | 2.30E-01 | 1.81E-04 | 9.70E-01 |
| rs5743618 | A | C | -9.15E-02 | 2.99E-01 | 6.70E-03 | 2.73E-42 |  | 7.82E-05 | 2.63E-01 | 1.84E-04 | 6.70E-01 |
| rs58939053 | T | C | 5.44E-02 | 3.44E-01 | 6.10E-03 | 3.85E-19 |  | -3.81E-05 | 3.24E-01 | 1.63E-04 | 8.10E-01 |
| rs6011033 | A | G | -4.37E-02 | 2.25E-01 | 6.90E-03 | 2.66E-10 |  | -3.17E-04 | 2.34E-01 | 1.81E-04 | 8.00E-02 |
| rs61192126 | T | C | 3.84E-02 | 3.10E-01 | 6.40E-03 | 1.56E-09 |  | -2.08E-04 | 7.06E-01 | 1.68E-04 | 2.10E-01 |
| rs61816766 | T | C | -1.42E-01 | 2.04E-02 | 1.76E-02 | 6.76E-16 |  | 5.79E-04 | 9.69E-01 | 4.50E-04 | 2.00E-01 |
| rs62626322 | T | G | -6.74E-02 | 9.69E-02 | 9.10E-03 | 1.35E-13 |  | 2.98E-05 | 8.87E-01 | 2.40E-04 | 9.00E-01 |
| rs6461503 | T | C | 4.10E-02 | 4.73E-01 | 5.70E-03 | 8.81E-13 |  | -2.31E-04 | 4.83E-01 | 1.52E-04 | 1.30E-01 |
| rs6489785 | T | C | 4.28E-02 | 3.69E-01 | 5.90E-03 | 3.57E-13 |  | -5.94E-05 | 3.80E-01 | 1.57E-04 | 7.10E-01 |
| rs6800001 | A | G | 3.30E-02 | 3.42E-01 | 5.90E-03 | 2.28E-08 |  | -4.47E-05 | 3.71E-01 | 1.58E-04 | 7.80E-01 |
| rs6881706 | T | G | -7.07E-02 | 2.93E-01 | 6.40E-03 | 1.73E-28 |  | -2.72E-05 | 2.71E-01 | 1.71E-04 | 8.70E-01 |
| rs6990534 | A | G | 4.00E-02 | 3.57E-01 | 6.30E-03 | 1.74E-10 |  | -2.05E-04 | 2.95E-01 | 1.66E-04 | 2.20E-01 |
| rs7224129 | A | G | 5.35E-02 | 4.73E-01 | 5.70E-03 | 8.11E-21 |  | -4.89E-07 | 4.86E-01 | 1.52E-04 | 1.00E+00 |
| rs72774901 | A | T | 1.12E-01 | 8.16E-02 | 1.08E-02 | 3.92E-25 |  | -4.22E-05 | 7.23E-02 | 2.95E-04 | 8.90E-01 |
| rs7406234 | T | C | 3.37E-02 | 4.35E-01 | 5.70E-03 | 4.30E-09 |  | 1.06E-05 | 4.43E-01 | 1.54E-04 | 9.50E-01 |
| rs74847330 | A | G | 4.97E-02 | 1.22E-01 | 8.80E-03 | 1.67E-08 |  | 1.09E-04 | 8.76E-01 | 2.30E-04 | 6.30E-01 |
| rs7521390 | A | C | -3.58E-02 | 2.84E-01 | 6.30E-03 | 1.40E-08 |  | 1.82E-05 | 7.01E-01 | 1.69E-04 | 9.10E-01 |
| rs7625643 | A | G | -3.46E-02 | 4.42E-01 | 5.80E-03 | 2.96E-09 |  | -2.28E-04 | 5.53E-01 | 1.55E-04 | 1.40E-01 |
| rs7712601 | T | C | 3.46E-02 | 4.18E-01 | 5.80E-03 | 3.28E-09 |  | -1.73E-05 | 3.90E-01 | 1.56E-04 | 9.10E-01 |
| rs80064395 | T | C | -7.26E-02 | 5.78E-02 | 1.07E-02 | 1.32E-11 |  | -5.12E-05 | 8.23E-02 | 2.78E-04 | 8.50E-01 |
| rs8030821 | A | T | -3.37E-02 | 3.90E-01 | 6.00E-03 | 1.54E-08 |  | -1.69E-04 | 3.90E-01 | 1.57E-04 | 2.80E-01 |
| rs848 | A | C | 6.01E-02 | 2.36E-01 | 7.30E-03 | 1.62E-16 |  | -1.88E-04 | 1.93E-01 | 1.94E-04 | 3.30E-01 |
| rs9372120 | T | G | -4.06E-02 | 1.75E-01 | 7.10E-03 | 1.02E-08 |  | -2.43E-05 | 7.97E-01 | 1.89E-04 | 9.00E-01 |
| rs9877752 | A | G | 3.98E-02 | 4.12E-01 | 5.80E-03 | 4.98E-12 |  | 3.72E-05 | 4.43E-01 | 1.53E-04 | 8.10E-01 |
| Abbreviation: SNP, single nucleotide polymorphism; EAF, effect allele frequency; SE, standard error.  **1.4 Mendelian randomization analysis of allergic diseases and kidney volume.** | | | | | | | | | | | |
| **Outcome:ebi-a-GCST90016670** | | | exposure | | | |  | outcome | | | |
| SNP | effect_allele | other_allele | beta | eaf | se | pval |  | beta | eaf | se | pval |
| rs10033073 | A | G | -4.37E-02 | 3.50E-01 | 7.20E-03 | 1.36E-09 |  | -2.98E-03 | 6.28E-01 | 6.40E-01 | 6.47E-03 |
| rs10414065 | T | C | -9.17E-02 | 7.82E-02 | 1.20E-02 | 2.29E-14 |  | 1.07E-02 | 6.71E-02 | 4.10E-01 | 1.28E-02 |
| rs10519067 | A | G | -5.18E-02 | 1.40E-01 | 8.50E-03 | 1.10E-09 |  | 9.98E-03 | 1.26E-01 | 3.00E-01 | 9.54E-03 |
| rs1059513 | T | C | 8.28E-02 | 1.11E-01 | 9.40E-03 | 1.15E-18 |  | -6.19E-03 | 8.92E-01 | 5.40E-01 | 1.02E-02 |
| rs10789841 | T | C | 4.56E-02 | 3.10E-01 | 6.40E-03 | 1.48E-12 |  | 6.40E-04 | 7.32E-01 | 9.30E-01 | 7.10E-03 |
| rs10865050 | A | G | -1.25E-01 | 1.53E-01 | 8.40E-03 | 6.37E-50 |  | 1.95E-03 | 1.40E-01 | 8.30E-01 | 9.02E-03 |
| rs11033545 | T | G | -3.34E-02 | 4.42E-01 | 6.00E-03 | 2.41E-08 |  | -3.55E-03 | 5.91E-01 | 5.80E-01 | 6.50E-03 |
| rs11168245 | C | G | 4.72E-02 | 1.99E-01 | 7.00E-03 | 1.41E-11 |  | 1.88E-03 | 2.42E-01 | 8.00E-01 | 7.36E-03 |
| rs11236814 | A | T | 6.53E-02 | 9.18E-02 | 9.80E-03 | 3.25E-11 |  | -1.43E-02 | 9.83E-02 | 1.80E-01 | 1.06E-02 |
| rs12123821 | T | C | 1.16E-01 | 4.76E-02 | 1.40E-02 | 1.04E-16 |  | -1.45E-02 | 4.79E-02 | 3.20E-01 | 1.47E-02 |
| rs1214598 | A | G | -4.01E-02 | 3.32E-01 | 6.00E-03 | 2.14E-11 |  | 1.40E-04 | 3.87E-01 | 9.80E-01 | 6.47E-03 |
| rs12365699 | A | G | -5.92E-02 | 1.53E-01 | 7.80E-03 | 3.54E-14 |  | -1.02E-02 | 1.66E-01 | 2.30E-01 | 8.48E-03 |
| rs12413578 | T | C | -9.34E-02 | 9.86E-02 | 9.40E-03 | 3.31E-23 |  | -3.91E-03 | 1.04E-01 | 7.00E-01 | 1.03E-02 |
| rs12440045 | A | C | -4.03E-02 | 4.52E-01 | 5.90E-03 | 7.68E-12 |  | 1.00E-02 | 4.74E-01 | 1.20E-01 | 6.39E-03 |
| rs12551834 | A | G | -6.15E-02 | 7.99E-02 | 1.03E-02 | 2.72E-09 |  | 1.54E-03 | 9.93E-02 | 8.80E-01 | 1.06E-02 |
| rs12625547 | T | G | 4.32E-02 | 2.14E-01 | 7.70E-03 | 1.98E-08 |  | 8.69E-03 | 8.21E-01 | 2.90E-01 | 8.25E-03 |
| rs1289273 | A | G | -3.50E-02 | 4.27E-01 | 5.70E-03 | 1.09E-09 |  | 6.29E-03 | 4.95E-01 | 3.20E-01 | 6.29E-03 |
| rs12941864 | T | C | -3.35E-02 | 4.66E-01 | 6.10E-03 | 4.98E-08 |  | -5.47E-03 | 5.20E-01 | 3.90E-01 | 6.39E-03 |
| rs1419675 | T | G | -3.85E-02 | 2.42E-01 | 7.00E-03 | 4.72E-08 |  | -3.69E-03 | 7.43E-01 | 6.10E-01 | 7.19E-03 |
| rs144829310 | T | G | 8.28E-02 | 1.57E-01 | 7.80E-03 | 2.60E-26 |  | -6.18E-03 | 1.61E-01 | 4.70E-01 | 8.57E-03 |
| rs16903574 | C | G | -7.00E-02 | 8.16E-02 | 1.10E-02 | 1.68E-10 |  | -1.62E-02 | 7.90E-02 | 1.70E-01 | 1.19E-02 |
| rs17664743 | A | G | 4.10E-02 | 2.04E-01 | 7.10E-03 | 8.27E-09 |  | 1.89E-03 | 2.04E-01 | 8.10E-01 | 7.83E-03 |
| rs1885013 | A | G | -3.82E-02 | 3.59E-01 | 6.30E-03 | 1.33E-09 |  | 9.84E-04 | 7.17E-01 | 8.90E-01 | 6.98E-03 |
| rs2134814 | C | G | 4.27E-02 | 3.54E-01 | 6.00E-03 | 1.03E-12 |  | 4.31E-03 | 3.54E-01 | 5.10E-01 | 6.57E-03 |
| rs2221641 | T | C | -4.19E-02 | 3.88E-01 | 5.90E-03 | 1.03E-12 |  | -2.23E-04 | 6.23E-01 | 9.70E-01 | 6.47E-03 |
| rs2241099 | C | G | 6.97E-02 | 2.62E-01 | 6.60E-03 | 4.62E-26 |  | -8.23E-03 | 2.47E-01 | 2.60E-01 | 7.27E-03 |
| rs228619 | A | G | -3.62E-02 | 4.69E-01 | 5.70E-03 | 2.41E-10 |  | 7.64E-03 | 5.03E-01 | 2.20E-01 | 6.26E-03 |
| rs2477923 | T | C | 3.32E-02 | 4.98E-01 | 5.70E-03 | 7.23E-09 |  | -9.44E-03 | 5.38E-01 | 1.40E-01 | 6.33E-03 |
| rs249677 | A | C | 3.66E-02 | 3.61E-01 | 5.90E-03 | 7.54E-10 |  | -1.23E-02 | 6.37E-01 | 6.10E-02 | 6.56E-03 |
| rs2766678 | A | G | -5.59E-02 | 2.13E-01 | 7.40E-03 | 2.95E-14 |  | -8.05E-03 | 7.86E-01 | 3.10E-01 | 7.87E-03 |
| rs2854001 | A | G | 5.37E-02 | 1.84E-01 | 7.10E-03 | 3.31E-14 |  | -6.04E-03 | 2.30E-01 | 4.20E-01 | 7.52E-03 |
| rs2910162 | A | G | -3.44E-02 | 3.16E-01 | 6.10E-03 | 1.54E-08 |  | 3.79E-03 | 3.22E-01 | 5.80E-01 | 6.77E-03 |
| rs3024665 | T | C | 6.65E-02 | 7.65E-02 | 1.19E-02 | 2.18E-08 |  | 2.12E-02 | 9.39E-01 | 1.10E-01 | 1.32E-02 |
| rs3128959 | A | G | -6.01E-02 | 1.24E-01 | 9.50E-03 | 2.25E-10 |  | -5.20E-06 | 1.22E-01 | 1.00E+00 | 9.70E-03 |
| rs34290285 | A | G | -7.66E-02 | 2.65E-01 | 7.50E-03 | 1.16E-24 |  | 5.08E-03 | 2.56E-01 | 4.80E-01 | 7.20E-03 |
| rs3540 | A | G | -3.46E-02 | 3.45E-01 | 6.10E-03 | 1.28E-08 |  | 8.75E-03 | 3.19E-01 | 2.00E-01 | 6.76E-03 |
| rs4296977 | T | C | -5.34E-02 | 1.58E-01 | 8.20E-03 | 6.92E-11 |  | -5.95E-04 | 8.59E-01 | 9.50E-01 | 9.11E-03 |
| rs4943794 | C | G | 3.96E-02 | 2.26E-01 | 7.00E-03 | 1.41E-08 |  | -1.12E-02 | 2.11E-01 | 1.50E-01 | 7.71E-03 |
| rs4973380 | A | T | 3.97E-02 | 2.43E-01 | 6.70E-03 | 2.59E-09 |  | 2.10E-03 | 2.43E-01 | 7.70E-01 | 7.31E-03 |
| rs519973 | A | G | 3.58E-02 | 3.54E-01 | 6.00E-03 | 3.23E-09 |  | -3.11E-03 | 3.39E-01 | 6.40E-01 | 6.62E-03 |
| rs56375023 | A | G | 7.31E-02 | 2.13E-01 | 6.80E-03 | 3.11E-27 |  | -6.95E-03 | 2.36E-01 | 3.50E-01 | 7.44E-03 |
| rs5743618 | A | C | -9.15E-02 | 2.99E-01 | 6.70E-03 | 2.73E-42 |  | 6.43E-03 | 2.21E-01 | 3.90E-01 | 7.55E-03 |
| rs5758343 | A | T | 4.74E-02 | 2.18E-01 | 7.10E-03 | 2.17E-11 |  | 2.13E-03 | 2.01E-01 | 7.90E-01 | 7.89E-03 |
| rs58939053 | T | C | 5.44E-02 | 3.44E-01 | 6.10E-03 | 3.85E-19 |  | 4.91E-03 | 3.35E-01 | 4.60E-01 | 6.65E-03 |
| rs6011033 | A | G | -4.37E-02 | 2.25E-01 | 6.90E-03 | 2.66E-10 |  | 1.45E-02 | 2.35E-01 | 5.20E-02 | 7.48E-03 |
| rs61192126 | T | C | 3.84E-02 | 3.10E-01 | 6.40E-03 | 1.56E-09 |  | 1.23E-02 | 7.07E-01 | 7.90E-02 | 7.02E-03 |
| rs61816766 | T | C | -1.42E-01 | 2.04E-02 | 1.76E-02 | 6.76E-16 |  | -1.99E-02 | 9.67E-01 | 2.70E-01 | 1.81E-02 |
| rs62626322 | T | G | -6.74E-02 | 9.69E-02 | 9.10E-03 | 1.35E-13 |  | -2.26E-02 | 8.87E-01 | 2.30E-02 | 9.96E-03 |
| rs6461503 | T | C | 4.10E-02 | 4.73E-01 | 5.70E-03 | 8.81E-13 |  | 1.12E-03 | 4.88E-01 | 8.60E-01 | 6.31E-03 |
| rs6489785 | T | C | 4.28E-02 | 3.69E-01 | 5.90E-03 | 3.57E-13 |  | 4.80E-03 | 3.90E-01 | 4.60E-01 | 6.50E-03 |
| rs6594499 | A | C | -7.23E-02 | 4.81E-01 | 5.70E-03 | 1.10E-36 |  | 1.89E-03 | 4.86E-01 | 7.60E-01 | 6.31E-03 |
| rs6800001 | A | G | 3.30E-02 | 3.42E-01 | 5.90E-03 | 2.28E-08 |  | -8.95E-03 | 3.75E-01 | 1.70E-01 | 6.52E-03 |
| rs6881706 | T | G | -7.07E-02 | 2.93E-01 | 6.40E-03 | 1.73E-28 |  | -8.31E-04 | 2.75E-01 | 9.10E-01 | 7.06E-03 |
| rs6990534 | A | G | 4.00E-02 | 3.57E-01 | 6.30E-03 | 1.74E-10 |  | 1.83E-02 | 2.93E-01 | 7.80E-03 | 6.89E-03 |
| rs7224129 | A | G | 5.35E-02 | 4.73E-01 | 5.70E-03 | 8.11E-21 |  | -1.88E-03 | 4.79E-01 | 7.70E-01 | 6.32E-03 |
| rs72774901 | A | T | 1.12E-01 | 8.16E-02 | 1.08E-02 | 3.92E-25 |  | 1.23E-02 | 7.40E-02 | 3.10E-01 | 1.20E-02 |
| rs7406234 | T | C | 3.37E-02 | 4.35E-01 | 5.70E-03 | 4.30E-09 |  | -1.30E-03 | 4.53E-01 | 8.40E-01 | 6.34E-03 |
| rs74847330 | A | G | 4.97E-02 | 1.22E-01 | 8.80E-03 | 1.67E-08 |  | -5.05E-03 | 8.77E-01 | 6.00E-01 | 9.54E-03 |
| rs7521390 | A | C | -3.58E-02 | 2.84E-01 | 6.30E-03 | 1.40E-08 |  | 6.17E-03 | 7.13E-01 | 3.80E-01 | 7.01E-03 |
| rs7625643 | A | G | -3.46E-02 | 4.42E-01 | 5.80E-03 | 2.96E-09 |  | -1.29E-02 | 5.53E-01 | 4.40E-02 | 6.40E-03 |
| rs7712601 | T | C | 3.46E-02 | 4.18E-01 | 5.80E-03 | 3.28E-09 |  | 1.36E-02 | 3.95E-01 | 3.40E-02 | 6.42E-03 |
| rs80064395 | T | C | -7.26E-02 | 5.78E-02 | 1.07E-02 | 1.32E-11 |  | 3.64E-03 | 8.48E-02 | 7.50E-01 | 1.13E-02 |
| rs8030821 | A | T | -3.37E-02 | 3.90E-01 | 6.00E-03 | 1.54E-08 |  | 7.59E-03 | 3.89E-01 | 2.40E-01 | 6.52E-03 |
| rs848 | A | C | 6.01E-02 | 2.36E-01 | 7.30E-03 | 1.62E-16 |  | 1.48E-02 | 1.83E-01 | 6.80E-02 | 8.12E-03 |
| rs9372120 | T | G | -4.06E-02 | 1.75E-01 | 7.10E-03 | 1.02E-08 |  | 7.07E-04 | 7.93E-01 | 9.30E-01 | 7.75E-03 |
| rs9877752 | A | G | 3.98E-02 | 4.12E-01 | 5.80E-03 | 4.98E-12 |  | 2.01E-03 | 4.52E-01 | 7.50E-01 | 6.27E-03 |
| Abbreviation: SNP, single nucleotide polymorphism; EAF, effect allele frequency; SE, standard error.  **1.5 Mendelian randomization analysis of allergic diseases and cyst of kidney.** | | | | | | | | | | | |
| **Outcome:finn-b-N14_CYSTKID** | | | exposure | | | |  | outcome | | | |
| SNP | effect_allele | other_allele | beta | eaf | se | pval |  | beta | eaf | se | pval |
| rs10033073 | A | G | -4.37E-02 | 3.50E-01 | 5.29E+03 | 7.20E-03 |  | 1.53E-02 | 6.10E-01 | 4.77E+06 | 6.65E-01 |
| rs10414065 | T | C | -9.17E-02 | 7.82E-02 | 2.73E+03 | 1.20E-02 |  | 5.33E-03 | 9.75E-02 | 3.32E+07 | 9.28E-01 |
| rs10519067 | A | G | -5.18E-02 | 1.40E-01 | 1.54E+03 | 8.50E-03 |  | -6.81E-02 | 1.49E-01 | 6.08E+07 | 1.58E-01 |
| rs1059513 | T | C | 8.28E-02 | 1.11E-01 | 1.42E+03 | 9.40E-03 |  | -1.02E-02 | 9.53E-01 | 5.71E+07 | 9.01E-01 |
| rs10789841 | T | C | 4.56E-02 | 3.10E-01 | 3.19E+02 | 6.40E-03 |  | 1.02E-02 | 7.67E-01 | 1.12E+08 | 8.03E-01 |
| rs11033545 | T | G | -3.34E-02 | 4.42E-01 | 7.16E+02 | 6.00E-03 |  | 3.78E-02 | 5.84E-01 | 3.63E+07 | 2.84E-01 |
| rs11168245 | C | G | 4.72E-02 | 1.99E-01 | 1.31E+03 | 7.00E-03 |  | -7.29E-03 | 2.51E-01 | 4.78E+07 | 8.55E-01 |
| rs11236814 | A | T | 6.53E-02 | 9.18E-02 | 1.04E+03 | 9.80E-03 |  | 1.54E-02 | 1.17E-01 | 7.66E+07 | 7.75E-01 |
| rs12123821 | T | C | 1.16E-01 | 4.76E-02 | 7.27E+02 | 1.40E-02 |  | 3.41E-02 | 3.89E-02 | 1.52E+08 | 7.02E-01 |
| rs1214598 | A | G | -4.01E-02 | 3.32E-01 | 8.09E+02 | 6.00E-03 |  | -6.61E-03 | 2.47E-01 | 1.67E+08 | 8.69E-01 |
| rs12365699 | A | G | -5.92E-02 | 1.53E-01 | 6.83E+02 | 7.80E-03 |  | -4.12E-02 | 1.58E-01 | 1.19E+08 | 3.83E-01 |
| rs12413578 | T | C | -9.34E-02 | 9.86E-02 | 1.30E+02 | 9.40E-03 |  | -5.20E-02 | 5.84E-02 | 9.01E+06 | 4.81E-01 |
| rs12440045 | A | C | -4.03E-02 | 4.52E-01 | 1.51E+03 | 5.90E-03 |  | 2.65E-02 | 4.84E-01 | 4.15E+07 | 4.46E-01 |
| rs12551834 | A | G | -6.15E-02 | 7.99E-02 | 8.70E+03 | 1.03E-02 |  | 1.04E-01 | 5.66E-02 | 1.29E+08 | 1.66E-01 |
| rs12625547 | T | G | 4.32E-02 | 2.14E-01 | 2.75E+03 | 7.70E-03 |  | 1.84E-02 | 8.32E-01 | 5.15E+07 | 6.92E-01 |
| rs1289273 | A | G | -3.50E-02 | 4.27E-01 | 1.29E+03 | 5.70E-03 |  | 3.87E-02 | 5.17E-01 | 2.27E+08 | 2.64E-01 |
| rs12941864 | T | C | -3.35E-02 | 4.66E-01 | 1.88E+03 | 6.10E-03 |  | -3.66E-02 | 5.52E-01 | 1.49E+06 | 2.94E-01 |
| rs1419675 | T | G | -3.85E-02 | 2.42E-01 | 6.52E+03 | 7.00E-03 |  | 3.94E-02 | 8.34E-01 | 3.01E+07 | 3.96E-01 |
| rs144829310 | T | G | 8.28E-02 | 1.57E-01 | 8.90E+03 | 7.80E-03 |  | -2.88E-02 | 1.48E-01 | 6.21E+06 | 5.52E-01 |
| rs1689510 | C | G | 5.10E-02 | 3.16E-01 | 1.36E+03 | 6.10E-03 |  | 2.19E-02 | 3.19E-01 | 5.60E+07 | 5.56E-01 |
| rs16903574 | C | G | -7.00E-02 | 8.16E-02 | 6.26E+03 | 1.10E-02 |  | -3.00E-02 | 1.28E-01 | 1.46E+07 | 5.68E-01 |
| rs17664743 | A | G | 4.10E-02 | 2.04E-01 | 8.56E+03 | 7.10E-03 |  | -6.93E-02 | 1.38E-01 | 5.02E+07 | 1.63E-01 |
| rs1885013 | A | G | -3.82E-02 | 3.59E-01 | 1.47E+03 | 6.30E-03 |  | -1.86E-02 | 6.95E-01 | 6.83E+07 | 6.21E-01 |
| rs2134814 | C | G | 4.27E-02 | 3.54E-01 | 8.36E+03 | 6.00E-03 |  | -2.69E-02 | 2.50E-01 | 9.03E+07 | 5.00E-01 |
| rs2212434 | T | C | 8.69E-02 | 4.29E-01 | 9.58E+02 | 5.70E-03 |  | 1.47E-02 | 4.05E-01 | 7.66E+07 | 6.76E-01 |
| rs2221641 | T | C | -4.19E-02 | 3.88E-01 | 8.67E+03 | 5.90E-03 |  | 1.27E-02 | 6.55E-01 | 8.04E+07 | 7.28E-01 |
| rs2241099 | C | G | 6.97E-02 | 2.62E-01 | 1.81E+03 | 6.60E-03 |  | 2.18E-03 | 2.11E-01 | 1.11E+07 | 9.59E-01 |
| rs228619 | A | G | -3.62E-02 | 4.69E-01 | 4.33E+03 | 5.70E-03 |  | -2.20E-03 | 4.68E-01 | 1.03E+08 | 9.49E-01 |
| rs2477923 | T | C | 3.32E-02 | 4.98E-01 | 6.10E+01 | 5.70E-03 |  | -8.45E-03 | 5.09E-01 | 8.52E+06 | 8.07E-01 |
| rs249677 | A | C | 3.66E-02 | 3.61E-01 | 6.25E+03 | 5.90E-03 |  | 8.47E-03 | 6.95E-01 | 1.42E+08 | 8.21E-01 |
| rs2766678 | A | G | -5.59E-02 | 2.13E-01 | 2.77E+03 | 7.40E-03 |  | 1.81E-02 | 8.18E-01 | 5.36E+07 | 6.87E-01 |
| rs2910162 | A | G | -3.44E-02 | 3.16E-01 | 6.27E+03 | 6.10E-03 |  | -5.00E-02 | 3.30E-01 | 1.60E+08 | 1.73E-01 |
| rs3024665 | T | C | 6.65E-02 | 7.65E-02 | 1.88E+03 | 1.19E-02 |  | 7.01E-02 | 9.15E-01 | 2.74E+07 | 2.60E-01 |
| rs3128959 | A | G | -6.01E-02 | 1.24E-01 | 8.21E+03 | 9.50E-03 |  | -3.55E-02 | 1.34E-01 | 3.31E+07 | 4.83E-01 |
| rs34004019 | A | G | 9.13E-02 | 3.04E-01 | 7.83E+03 | 7.10E-03 |  | -4.67E-02 | 6.62E-01 | 3.27E+07 | 2.01E-01 |
| rs34290285 | A | G | -7.66E-02 | 2.65E-01 | 3.87E+03 | 7.50E-03 |  | 5.12E-03 | 2.17E-01 | 2.42E+08 | 9.03E-01 |
| rs3540 | A | G | -3.46E-02 | 3.45E-01 | 1.59E+03 | 6.10E-03 |  | -6.02E-03 | 3.28E-01 | 9.05E+07 | 8.70E-01 |
| rs4296977 | T | C | -5.34E-02 | 1.58E-01 | 8.58E+03 | 8.20E-03 |  | 8.26E-02 | 8.03E-01 | 7.74E+07 | 5.73E-02 |
| rs479844 | A | G | -4.12E-02 | 4.29E-01 | 7.84E+02 | 5.80E-03 |  | -1.24E-02 | 4.12E-01 | 6.58E+07 | 7.25E-01 |
| rs4973380 | A | T | 3.97E-02 | 2.43E-01 | 3.38E+03 | 6.70E-03 |  | -4.99E-02 | 2.87E-01 | 2.28E+08 | 1.91E-01 |
| rs519973 | A | G | 3.58E-02 | 3.54E-01 | 4.11E+03 | 6.00E-03 |  | 3.31E-02 | 3.59E-01 | 1.88E+08 | 3.57E-01 |
| rs5743618 | A | C | -9.15E-02 | 2.99E-01 | 5.02E+03 | 6.70E-03 |  | 4.38E-02 | 1.54E-01 | 3.88E+07 | 3.60E-01 |
| rs58939053 | T | C | 5.44E-02 | 3.44E-01 | 4.66E+03 | 6.10E-03 |  | -4.33E-02 | 4.55E-01 | 1.22E+08 | 2.12E-01 |
| rs6011033 | A | G | -4.37E-02 | 2.25E-01 | 2.80E+03 | 6.90E-03 |  | 1.18E-02 | 1.91E-01 | 6.37E+07 | 7.88E-01 |
| rs61192126 | T | C | 3.84E-02 | 3.10E-01 | 4.25E+03 | 6.40E-03 |  | 2.49E-02 | 7.32E-01 | 7.23E+07 | 5.22E-01 |
| rs61816766 | T | C | -1.42E-01 | 2.04E-02 | 7.30E+02 | 1.76E-02 |  | 1.16E-01 | 9.92E-01 | 1.52E+08 | 5.61E-01 |
| rs62626322 | T | G | -6.74E-02 | 9.69E-02 | 3.10E+01 | 9.10E-03 |  | -4.51E-02 | 8.59E-01 | 6.07E+06 | 3.63E-01 |
| rs6461503 | T | C | 4.10E-02 | 4.73E-01 | 8.51E+03 | 5.70E-03 |  | 4.87E-02 | 4.79E-01 | 2.05E+07 | 1.59E-01 |
| rs6489785 | T | C | 4.28E-02 | 3.69E-01 | 1.27E+03 | 5.90E-03 |  | 2.01E-04 | 3.85E-01 | 1.21E+08 | 9.96E-01 |
| rs6594499 | A | C | -7.23E-02 | 4.81E-01 | 5.74E+03 | 5.70E-03 |  | 5.68E-02 | 5.53E-01 | 1.11E+08 | 1.02E-01 |
| rs6800001 | A | G | 3.30E-02 | 3.42E-01 | 4.25E+03 | 5.90E-03 |  | 3.00E-02 | 3.31E-01 | 3.30E+07 | 4.17E-01 |
| rs6881706 | T | G | -7.07E-02 | 2.93E-01 | 6.32E+03 | 6.40E-03 |  | -1.68E-03 | 3.31E-01 | 3.59E+07 | 9.64E-01 |
| rs6990534 | A | G | 4.00E-02 | 3.57E-01 | 8.59E+03 | 6.30E-03 |  | -2.32E-02 | 3.28E-01 | 1.28E+08 | 5.27E-01 |
| rs7224129 | A | G | 5.35E-02 | 4.73E-01 | 2.07E+03 | 5.70E-03 |  | -8.71E-03 | 4.49E-01 | 3.99E+07 | 8.02E-01 |
| rs72774901 | A | T | 1.12E-01 | 8.16E-02 | 5.57E+03 | 1.08E-02 |  | -2.79E-02 | 8.23E-02 | 1.11E+08 | 6.59E-01 |
| rs7406234 | T | C | 3.37E-02 | 4.35E-01 | 2.44E+03 | 5.70E-03 |  | 4.44E-02 | 4.91E-01 | 4.93E+07 | 1.99E-01 |
| rs74847330 | A | G | 4.97E-02 | 1.22E-01 | 3.35E+03 | 8.80E-03 |  | -6.33E-02 | 9.15E-01 | 1.43E+08 | 3.05E-01 |
| rs7521390 | A | C | -3.58E-02 | 2.84E-01 | 8.31E+02 | 6.30E-03 |  | 2.54E-02 | 7.00E-01 | 1.73E+08 | 5.02E-01 |
| rs7625643 | A | G | -3.46E-02 | 4.42E-01 | 4.03E+03 | 5.80E-03 |  | -2.13E-02 | 5.40E-01 | 1.41E+08 | 5.40E-01 |
| rs7712601 | T | C | 3.46E-02 | 4.18E-01 | 6.17E+03 | 5.80E-03 |  | 1.67E-02 | 3.85E-01 | 1.42E+08 | 6.38E-01 |
| rs80064395 | T | C | -7.26E-02 | 5.78E-02 | 4.23E+03 | 1.07E-02 |  | 3.17E-02 | 9.69E-02 | 1.97E+08 | 5.90E-01 |
| rs8030821 | A | T | -3.37E-02 | 3.90E-01 | 1.58E+03 | 6.00E-03 |  | -1.72E-02 | 3.43E-01 | 7.03E+07 | 6.37E-01 |
| rs848 | A | C | 6.01E-02 | 2.36E-01 | 6.09E+03 | 7.30E-03 |  | -1.86E-02 | 3.69E-01 | 1.33E+08 | 6.06E-01 |
| rs9372120 | T | G | -4.06E-02 | 1.75E-01 | 6.40E+03 | 7.10E-03 |  | -1.80E-02 | 7.65E-01 | 1.06E+08 | 6.59E-01 |
| rs9877752 | A | G | 3.98E-02 | 4.12E-01 | 4.13E+03 | 5.80E-03 |  | -6.01E-02 | 3.84E-01 | 1.88E+08 | 9.10E-02 |
| Abbreviation: SNP, single nucleotide polymorphism; EAF, effect allele frequency; SE, standard error.  **1.6 Mendelian randomization analysis of allergic diseases and chronic kidney disease.** | | | | | | | | | | | |
| **Outcome:finn-b-N14_CHRONKIDNEYDIS** | | | exposure | | | |  | outcome | | | |
| SNP | effect_allele | other_allele | beta | eaf | se | pval |  | beta | eaf | se | pval |
| rs10033073 | A | G | -4.37E-02 | 3.50E-01 | 7.20E-03 | 1.36E-09 |  | 1.42E-02 | 6.10E-01 | 1.57E-02 | 3.64E-01 |
| rs10174949 | A | G | -6.56E-02 | 3.16E-01 | 6.30E-03 | 9.92E-26 |  | -2.73E-02 | 3.46E-01 | 1.61E-02 | 9.08E-02 |
| rs10414065 | T | C | -9.17E-02 | 7.82E-02 | 1.20E-02 | 2.29E-14 |  | 3.30E-02 | 9.74E-02 | 2.60E-02 | 2.03E-01 |
| rs10519067 | A | G | -5.18E-02 | 1.40E-01 | 8.50E-03 | 1.10E-09 |  | 2.43E-02 | 1.49E-01 | 2.14E-02 | 2.56E-01 |
| rs1059513 | T | C | 8.28E-02 | 1.11E-01 | 9.40E-03 | 1.15E-18 |  | -3.06E-03 | 9.53E-01 | 3.59E-02 | 9.32E-01 |
| rs10865050 | A | G | -1.25E-01 | 1.53E-01 | 8.40E-03 | 6.37E-50 |  | -5.07E-03 | 1.62E-01 | 2.09E-02 | 8.08E-01 |
| rs11033545 | T | G | -3.34E-02 | 4.42E-01 | 6.00E-03 | 2.41E-08 |  | 2.22E-02 | 5.84E-01 | 1.56E-02 | 1.53E-01 |
| rs11168245 | C | G | 4.72E-02 | 1.99E-01 | 7.00E-03 | 1.41E-11 |  | 2.20E-03 | 2.51E-01 | 1.76E-02 | 9.00E-01 |
| rs11236814 | A | T | 6.53E-02 | 9.18E-02 | 9.80E-03 | 3.25E-11 |  | 6.74E-03 | 1.17E-01 | 2.40E-02 | 7.79E-01 |
| rs12123821 | T | C | 1.16E-01 | 4.76E-02 | 1.40E-02 | 1.04E-16 |  | -3.73E-04 | 3.90E-02 | 4.01E-02 | 9.93E-01 |
| rs1214598 | A | G | -4.01E-02 | 3.32E-01 | 6.00E-03 | 2.14E-11 |  | 5.32E-03 | 2.47E-01 | 1.76E-02 | 7.63E-01 |
| rs12365699 | A | G | -5.92E-02 | 1.53E-01 | 7.80E-03 | 3.54E-14 |  | -4.69E-02 | 1.57E-01 | 2.08E-02 | 2.44E-02 |
| rs12413578 | T | C | -9.34E-02 | 9.86E-02 | 9.40E-03 | 3.31E-23 |  | 4.65E-02 | 5.84E-02 | 3.26E-02 | 1.53E-01 |
| rs12551834 | A | G | -6.15E-02 | 7.99E-02 | 1.03E-02 | 2.72E-09 |  | -7.30E-03 | 5.66E-02 | 3.29E-02 | 8.25E-01 |
| rs12625547 | T | G | 4.32E-02 | 2.14E-01 | 7.70E-03 | 1.98E-08 |  | 5.60E-02 | 8.32E-01 | 2.07E-02 | 6.92E-03 |
| rs1289273 | A | G | -3.50E-02 | 4.27E-01 | 5.70E-03 | 1.09E-09 |  | 5.87E-04 | 5.17E-01 | 1.53E-02 | 9.69E-01 |
| rs12941864 | T | C | -3.35E-02 | 4.66E-01 | 6.10E-03 | 4.98E-08 |  | -2.10E-02 | 5.52E-01 | 1.54E-02 | 1.73E-01 |
| rs144829310 | T | G | 8.28E-02 | 1.57E-01 | 7.80E-03 | 2.60E-26 |  | 2.75E-02 | 1.48E-01 | 2.16E-02 | 2.04E-01 |
| rs1689510 | C | G | 5.10E-02 | 3.16E-01 | 6.10E-03 | 3.39E-17 |  | -1.27E-02 | 3.19E-01 | 1.63E-02 | 4.35E-01 |
| rs16903574 | C | G | -7.00E-02 | 8.16E-02 | 1.10E-02 | 1.68E-10 |  | 2.98E-02 | 1.28E-01 | 2.34E-02 | 2.03E-01 |
| rs17664743 | A | G | 4.10E-02 | 2.04E-01 | 7.10E-03 | 8.27E-09 |  | 8.32E-03 | 1.38E-01 | 2.20E-02 | 7.05E-01 |
| rs1885013 | A | G | -3.82E-02 | 3.59E-01 | 6.30E-03 | 1.33E-09 |  | -1.48E-02 | 6.94E-01 | 1.66E-02 | 3.73E-01 |
| rs2134814 | C | G | 4.27E-02 | 3.54E-01 | 6.00E-03 | 1.03E-12 |  | -9.69E-03 | 2.50E-01 | 1.76E-02 | 5.81E-01 |
| rs2212434 | T | C | 8.69E-02 | 4.29E-01 | 5.70E-03 | 8.93E-52 |  | 3.58E-02 | 4.05E-01 | 1.55E-02 | 2.12E-02 |
| rs2221641 | T | C | -4.19E-02 | 3.88E-01 | 5.90E-03 | 1.03E-12 |  | 8.10E-03 | 6.55E-01 | 1.61E-02 | 6.14E-01 |
| rs2241099 | C | G | 6.97E-02 | 2.62E-01 | 6.60E-03 | 4.62E-26 |  | -1.64E-02 | 2.11E-01 | 1.88E-02 | 3.81E-01 |
| rs2477923 | T | C | 3.32E-02 | 4.98E-01 | 5.70E-03 | 7.23E-09 |  | 2.40E-02 | 5.09E-01 | 1.53E-02 | 1.15E-01 |
| rs249677 | A | C | 3.66E-02 | 3.61E-01 | 5.90E-03 | 7.54E-10 |  | 1.31E-02 | 6.95E-01 | 1.65E-02 | 4.27E-01 |
| rs2766678 | A | G | -5.59E-02 | 2.13E-01 | 7.40E-03 | 2.95E-14 |  | 2.25E-02 | 8.18E-01 | 2.00E-02 | 2.62E-01 |
| rs2910162 | A | G | -3.44E-02 | 3.16E-01 | 6.10E-03 | 1.54E-08 |  | -1.58E-02 | 3.30E-01 | 1.62E-02 | 3.29E-01 |
| rs3024665 | T | C | 6.65E-02 | 7.65E-02 | 1.19E-02 | 2.18E-08 |  | -2.50E-02 | 9.15E-01 | 2.75E-02 | 3.62E-01 |
| rs3128959 | A | G | -6.01E-02 | 1.24E-01 | 9.50E-03 | 2.25E-10 |  | 4.83E-03 | 1.34E-01 | 2.24E-02 | 8.29E-01 |
| rs34004019 | A | G | 9.13E-02 | 3.04E-01 | 7.10E-03 | 2.52E-38 |  | 4.44E-02 | 6.62E-01 | 1.62E-02 | 6.23E-03 |
| rs34290285 | A | G | -7.66E-02 | 2.65E-01 | 7.50E-03 | 1.16E-24 |  | 1.17E-02 | 2.17E-01 | 1.85E-02 | 5.26E-01 |
| rs3540 | A | G | -3.46E-02 | 3.45E-01 | 6.10E-03 | 1.28E-08 |  | 5.15E-03 | 3.28E-01 | 1.62E-02 | 7.51E-01 |
| rs4296977 | T | C | -5.34E-02 | 1.58E-01 | 8.20E-03 | 6.92E-11 |  | -1.86E-02 | 8.03E-01 | 1.92E-02 | 3.35E-01 |
| rs479844 | A | G | -4.12E-02 | 4.29E-01 | 5.80E-03 | 1.15E-12 |  | -4.14E-02 | 4.12E-01 | 1.55E-02 | 7.50E-03 |
| rs4943794 | C | G | 3.96E-02 | 2.26E-01 | 7.00E-03 | 1.41E-08 |  | 2.59E-02 | 2.36E-01 | 1.79E-02 | 1.48E-01 |
| rs4973380 | A | T | 3.97E-02 | 2.43E-01 | 6.70E-03 | 2.59E-09 |  | 4.18E-03 | 2.87E-01 | 1.68E-02 | 8.04E-01 |
| rs519973 | A | G | 3.58E-02 | 3.54E-01 | 6.00E-03 | 3.23E-09 |  | -2.09E-02 | 3.59E-01 | 1.59E-02 | 1.89E-01 |
| rs5743618 | A | C | -9.15E-02 | 2.99E-01 | 6.70E-03 | 2.73E-42 |  | -3.18E-02 | 1.54E-01 | 2.11E-02 | 1.32E-01 |
| rs58939053 | T | C | 5.44E-02 | 3.44E-01 | 6.10E-03 | 3.85E-19 |  | 1.62E-02 | 4.55E-01 | 1.53E-02 | 2.90E-01 |
| rs6011033 | A | G | -4.37E-02 | 2.25E-01 | 6.90E-03 | 2.66E-10 |  | -3.53E-02 | 1.91E-01 | 1.94E-02 | 6.84E-02 |
| rs61192126 | T | C | 3.84E-02 | 3.10E-01 | 6.40E-03 | 1.56E-09 |  | -2.61E-02 | 7.33E-01 | 1.72E-02 | 1.29E-01 |
| rs61816766 | T | C | -1.42E-01 | 2.04E-02 | 1.76E-02 | 6.76E-16 |  | 2.16E-02 | 9.92E-01 | 8.48E-02 | 7.99E-01 |
| rs62626322 | T | G | -6.74E-02 | 9.69E-02 | 9.10E-03 | 1.35E-13 |  | -2.36E-02 | 8.59E-01 | 2.22E-02 | 2.87E-01 |
| rs6461503 | T | C | 4.10E-02 | 4.73E-01 | 5.70E-03 | 8.81E-13 |  | 6.17E-03 | 4.79E-01 | 1.53E-02 | 6.86E-01 |
| rs6489785 | T | C | 4.28E-02 | 3.69E-01 | 5.90E-03 | 3.57E-13 |  | -1.58E-02 | 3.85E-01 | 1.57E-02 | 3.14E-01 |
| rs6594499 | A | C | -7.23E-02 | 4.81E-01 | 5.70E-03 | 1.10E-36 |  | 1.14E-02 | 5.53E-01 | 1.53E-02 | 4.56E-01 |
| rs6800001 | A | G | 3.30E-02 | 3.42E-01 | 5.90E-03 | 2.28E-08 |  | -3.11E-03 | 3.31E-01 | 1.63E-02 | 8.49E-01 |
| rs6881706 | T | G | -7.07E-02 | 2.93E-01 | 6.40E-03 | 1.73E-28 |  | -1.41E-02 | 3.31E-01 | 1.62E-02 | 3.84E-01 |
| rs6990534 | A | G | 4.00E-02 | 3.57E-01 | 6.30E-03 | 1.74E-10 |  | -1.28E-03 | 3.28E-01 | 1.62E-02 | 9.37E-01 |
| rs7224129 | A | G | 5.35E-02 | 4.73E-01 | 5.70E-03 | 8.11E-21 |  | 4.16E-02 | 4.49E-01 | 1.53E-02 | 6.61E-03 |
| rs72774901 | A | T | 1.12E-01 | 8.16E-02 | 1.08E-02 | 3.92E-25 |  | 1.41E-02 | 8.22E-02 | 2.76E-02 | 6.11E-01 |
| rs74847330 | A | G | 4.97E-02 | 1.22E-01 | 8.80E-03 | 1.67E-08 |  | -1.89E-03 | 9.15E-01 | 2.72E-02 | 9.45E-01 |
| rs7521390 | A | C | -3.58E-02 | 2.84E-01 | 6.30E-03 | 1.40E-08 |  | -2.51E-02 | 7.00E-01 | 1.67E-02 | 1.34E-01 |
| rs7625643 | A | G | -3.46E-02 | 4.42E-01 | 5.80E-03 | 2.96E-09 |  | -1.43E-03 | 5.40E-01 | 1.53E-02 | 9.26E-01 |
| rs7712601 | T | C | 3.46E-02 | 4.18E-01 | 5.80E-03 | 3.28E-09 |  | 4.44E-03 | 3.85E-01 | 1.57E-02 | 7.77E-01 |
| rs80064395 | T | C | -7.26E-02 | 5.78E-02 | 1.07E-02 | 1.32E-11 |  | -4.95E-02 | 9.69E-02 | 2.58E-02 | 5.49E-02 |
| rs8030821 | A | T | -3.37E-02 | 3.90E-01 | 6.00E-03 | 1.54E-08 |  | 3.51E-03 | 3.43E-01 | 1.61E-02 | 8.27E-01 |
| rs848 | A | C | 6.01E-02 | 2.36E-01 | 7.30E-03 | 1.62E-16 |  | -2.21E-04 | 3.69E-01 | 1.59E-02 | 9.89E-01 |
| rs9372120 | T | G | -4.06E-02 | 1.75E-01 | 7.10E-03 | 1.02E-08 |  | -1.31E-02 | 7.64E-01 | 1.79E-02 | 4.66E-01 |
| rs9877752 | A | G | 3.98E-02 | 4.12E-01 | 5.80E-03 | 4.98E-12 |  | -3.79E-02 | 3.84E-01 | 1.57E-02 | 1.57E-02 |
| Abbreviation: SNP, single nucleotide polymorphism; EAF, effect allele frequency; SE, standard error.  **1.7 Mendelian randomization analysis of allergic diseases and unspecified kidney failure.** | | | | | | | | | | | |
| **Outcome:finn-b-N14_RENFAILNAS** | | | exposure | | | |  | outcome | | | |
| SNP | effect_allele | other_allele | beta | eaf | se | pval |  | beta | eaf | se | pval |
| rs10033073 | A | G | -4.37E-02 | 3.50E-01 | 7.20E-03 | 1.36E-09 |  | -2.64E-02 | 6.10E-01 | 2.89E-02 | 3.61E-01 |
| rs10174949 | A | G | -6.56E-02 | 3.16E-01 | 6.30E-03 | 9.92E-26 |  | -4.94E-02 | 3.46E-01 | 2.97E-02 | 9.70E-02 |
| rs10414065 | T | C | -9.17E-02 | 7.82E-02 | 1.20E-02 | 2.29E-14 |  | 2.64E-02 | 9.73E-02 | 4.79E-02 | 5.82E-01 |
| rs10519067 | A | G | -5.18E-02 | 1.40E-01 | 8.50E-03 | 1.10E-09 |  | -3.53E-02 | 1.49E-01 | 3.95E-02 | 3.71E-01 |
| rs1059513 | T | C | 8.28E-02 | 1.11E-01 | 9.40E-03 | 1.15E-18 |  | -2.64E-02 | 9.53E-01 | 6.66E-02 | 6.92E-01 |
| rs10865050 | A | G | -1.25E-01 | 1.53E-01 | 8.40E-03 | 6.37E-50 |  | -2.98E-02 | 1.62E-01 | 3.85E-02 | 4.38E-01 |
| rs11033545 | T | G | -3.34E-02 | 4.42E-01 | 6.00E-03 | 2.41E-08 |  | -2.18E-04 | 5.84E-01 | 2.87E-02 | 9.94E-01 |
| rs11168245 | C | G | 4.72E-02 | 1.99E-01 | 7.00E-03 | 1.41E-11 |  | 5.87E-02 | 2.51E-01 | 3.24E-02 | 7.00E-02 |
| rs11236814 | A | T | 6.53E-02 | 9.18E-02 | 9.80E-03 | 3.25E-11 |  | 1.98E-02 | 1.17E-01 | 4.43E-02 | 6.55E-01 |
| rs12123821 | T | C | 1.16E-01 | 4.76E-02 | 1.40E-02 | 1.04E-16 |  | 3.30E-02 | 3.90E-02 | 7.38E-02 | 6.55E-01 |
| rs1214598 | A | G | -4.01E-02 | 3.32E-01 | 6.00E-03 | 2.14E-11 |  | 3.13E-03 | 2.47E-01 | 3.25E-02 | 9.23E-01 |
| rs12365699 | A | G | -5.92E-02 | 1.53E-01 | 7.80E-03 | 3.54E-14 |  | -6.41E-02 | 1.57E-01 | 3.85E-02 | 9.64E-02 |
| rs12413578 | T | C | -9.34E-02 | 9.86E-02 | 9.40E-03 | 3.31E-23 |  | 2.36E-02 | 5.83E-02 | 5.99E-02 | 6.94E-01 |
| rs12551834 | A | G | -6.15E-02 | 7.99E-02 | 1.03E-02 | 2.72E-09 |  | 5.48E-04 | 5.66E-02 | 6.09E-02 | 9.93E-01 |
| rs12625547 | T | G | 4.32E-02 | 2.14E-01 | 7.70E-03 | 1.98E-08 |  | 1.89E-02 | 8.32E-01 | 3.78E-02 | 6.18E-01 |
| rs1289273 | A | G | -3.50E-02 | 4.27E-01 | 5.70E-03 | 1.09E-09 |  | 6.45E-03 | 5.17E-01 | 2.82E-02 | 8.19E-01 |
| rs12941864 | T | C | -3.35E-02 | 4.66E-01 | 6.10E-03 | 4.98E-08 |  | 5.39E-02 | 5.52E-01 | 2.84E-02 | 5.78E-02 |
| rs144829310 | T | G | 8.28E-02 | 1.57E-01 | 7.80E-03 | 2.60E-26 |  | 3.13E-03 | 1.48E-01 | 3.99E-02 | 9.37E-01 |
| rs1689510 | C | G | 5.10E-02 | 3.16E-01 | 6.10E-03 | 3.39E-17 |  | 1.28E-02 | 3.19E-01 | 3.01E-02 | 6.70E-01 |
| rs16903574 | C | G | -7.00E-02 | 8.16E-02 | 1.10E-02 | 1.68E-10 |  | 5.98E-02 | 1.28E-01 | 4.31E-02 | 1.66E-01 |
| rs17664743 | A | G | 4.10E-02 | 2.04E-01 | 7.10E-03 | 8.27E-09 |  | -3.35E-02 | 1.38E-01 | 4.06E-02 | 4.10E-01 |
| rs1885013 | A | G | -3.82E-02 | 3.59E-01 | 6.30E-03 | 1.33E-09 |  | 9.80E-03 | 6.94E-01 | 3.07E-02 | 7.49E-01 |
| rs2134814 | C | G | 4.27E-02 | 3.54E-01 | 6.00E-03 | 1.03E-12 |  | 6.41E-02 | 2.50E-01 | 3.24E-02 | 4.81E-02 |
| rs2212434 | T | C | 8.69E-02 | 4.29E-01 | 5.70E-03 | 8.93E-52 |  | 1.78E-02 | 4.05E-01 | 2.87E-02 | 5.35E-01 |
| rs2221641 | T | C | -4.19E-02 | 3.88E-01 | 5.90E-03 | 1.03E-12 |  | 1.24E-02 | 6.55E-01 | 2.97E-02 | 6.77E-01 |
| rs2241099 | C | G | 6.97E-02 | 2.62E-01 | 6.60E-03 | 4.62E-26 |  | 4.01E-02 | 2.11E-01 | 3.46E-02 | 2.46E-01 |
| rs2477923 | T | C | 3.32E-02 | 4.98E-01 | 5.70E-03 | 7.23E-09 |  | -7.56E-03 | 5.08E-01 | 2.81E-02 | 7.88E-01 |
| rs249677 | A | C | 3.66E-02 | 3.61E-01 | 5.90E-03 | 7.54E-10 |  | 3.51E-02 | 6.95E-01 | 3.05E-02 | 2.50E-01 |
| rs2766678 | A | G | -5.59E-02 | 2.13E-01 | 7.40E-03 | 2.95E-14 |  | -1.29E-02 | 8.18E-01 | 3.70E-02 | 7.27E-01 |
| rs2910162 | A | G | -3.44E-02 | 3.16E-01 | 6.10E-03 | 1.54E-08 |  | -3.57E-03 | 3.30E-01 | 2.99E-02 | 9.05E-01 |
| rs3024665 | T | C | 6.65E-02 | 7.65E-02 | 1.19E-02 | 2.18E-08 |  | -2.89E-02 | 9.15E-01 | 5.06E-02 | 5.68E-01 |
| rs3128959 | A | G | -6.01E-02 | 1.24E-01 | 9.50E-03 | 2.25E-10 |  | 1.62E-02 | 1.34E-01 | 4.14E-02 | 6.96E-01 |
| rs34004019 | A | G | 9.13E-02 | 3.04E-01 | 7.10E-03 | 2.52E-38 |  | 7.69E-02 | 6.62E-01 | 2.99E-02 | 1.00E-02 |
| rs34290285 | A | G | -7.66E-02 | 2.65E-01 | 7.50E-03 | 1.16E-24 |  | 2.14E-02 | 2.17E-01 | 3.42E-02 | 5.31E-01 |
| rs3540 | A | G | -3.46E-02 | 3.45E-01 | 6.10E-03 | 1.28E-08 |  | -2.67E-03 | 3.28E-01 | 2.99E-02 | 9.29E-01 |
| rs4296977 | T | C | -5.34E-02 | 1.58E-01 | 8.20E-03 | 6.92E-11 |  | 1.79E-02 | 8.03E-01 | 3.54E-02 | 6.14E-01 |
| rs479844 | A | G | -4.12E-02 | 4.29E-01 | 5.80E-03 | 1.15E-12 |  | -2.40E-03 | 4.12E-01 | 2.86E-02 | 9.33E-01 |
| rs4943794 | C | G | 3.96E-02 | 2.26E-01 | 7.00E-03 | 1.41E-08 |  | 1.02E-04 | 2.36E-01 | 3.31E-02 | 9.98E-01 |
| rs4973380 | A | T | 3.97E-02 | 2.43E-01 | 6.70E-03 | 2.59E-09 |  | 2.89E-02 | 2.87E-01 | 3.10E-02 | 3.51E-01 |
| rs519973 | A | G | 3.58E-02 | 3.54E-01 | 6.00E-03 | 3.23E-09 |  | -4.26E-02 | 3.59E-01 | 2.93E-02 | 1.46E-01 |
| rs5743618 | A | C | -9.15E-02 | 2.99E-01 | 6.70E-03 | 2.73E-42 |  | -1.42E-02 | 1.54E-01 | 3.90E-02 | 7.16E-01 |
| rs58939053 | T | C | 5.44E-02 | 3.44E-01 | 6.10E-03 | 3.85E-19 |  | 1.25E-02 | 4.55E-01 | 2.83E-02 | 6.57E-01 |
| rs6011033 | A | G | -4.37E-02 | 2.25E-01 | 6.90E-03 | 2.66E-10 |  | -4.65E-02 | 1.91E-01 | 3.58E-02 | 1.93E-01 |
| rs61192126 | T | C | 3.84E-02 | 3.10E-01 | 6.40E-03 | 1.56E-09 |  | -5.89E-02 | 7.33E-01 | 3.18E-02 | 6.36E-02 |
| rs61816766 | T | C | -1.42E-01 | 2.04E-02 | 1.76E-02 | 6.76E-16 |  | 3.15E-02 | 9.92E-01 | 1.60E-01 | 8.44E-01 |
| rs62626322 | T | G | -6.74E-02 | 9.69E-02 | 9.10E-03 | 1.35E-13 |  | -8.43E-03 | 8.59E-01 | 4.08E-02 | 8.36E-01 |
| rs6461503 | T | C | 4.10E-02 | 4.73E-01 | 5.70E-03 | 8.81E-13 |  | 1.53E-02 | 4.79E-01 | 2.82E-02 | 5.88E-01 |
| rs6489785 | T | C | 4.28E-02 | 3.69E-01 | 5.90E-03 | 3.57E-13 |  | -6.08E-02 | 3.85E-01 | 2.90E-02 | 3.60E-02 |
| rs6594499 | A | C | -7.23E-02 | 4.81E-01 | 5.70E-03 | 1.10E-36 |  | 1.89E-02 | 5.53E-01 | 2.83E-02 | 5.05E-01 |
| rs6800001 | A | G | 3.30E-02 | 3.42E-01 | 5.90E-03 | 2.28E-08 |  | 1.64E-02 | 3.31E-01 | 3.01E-02 | 5.86E-01 |
| rs6881706 | T | G | -7.07E-02 | 2.93E-01 | 6.40E-03 | 1.73E-28 |  | 3.36E-02 | 3.31E-01 | 2.99E-02 | 2.62E-01 |
| rs6990534 | A | G | 4.00E-02 | 3.57E-01 | 6.30E-03 | 1.74E-10 |  | 6.27E-02 | 3.28E-01 | 2.99E-02 | 3.62E-02 |
| rs7224129 | A | G | 5.35E-02 | 4.73E-01 | 5.70E-03 | 8.11E-21 |  | 4.22E-02 | 4.48E-01 | 2.83E-02 | 1.37E-01 |
| rs72774901 | A | T | 1.12E-01 | 8.16E-02 | 1.08E-02 | 3.92E-25 |  | 4.03E-02 | 8.22E-02 | 5.11E-02 | 4.31E-01 |
| rs74847330 | A | G | 4.97E-02 | 1.22E-01 | 8.80E-03 | 1.67E-08 |  | 2.55E-02 | 9.15E-01 | 5.04E-02 | 6.12E-01 |
| rs7521390 | A | C | -3.58E-02 | 2.84E-01 | 6.30E-03 | 1.40E-08 |  | -3.93E-02 | 7.00E-01 | 3.09E-02 | 2.02E-01 |
| rs7625643 | A | G | -3.46E-02 | 4.42E-01 | 5.80E-03 | 2.96E-09 |  | 1.33E-02 | 5.40E-01 | 2.83E-02 | 6.37E-01 |
| rs7712601 | T | C | 3.46E-02 | 4.18E-01 | 5.80E-03 | 3.28E-09 |  | 6.42E-02 | 3.85E-01 | 2.90E-02 | 2.66E-02 |
| rs80064395 | T | C | -7.26E-02 | 5.78E-02 | 1.07E-02 | 1.32E-11 |  | -3.76E-02 | 9.69E-02 | 4.77E-02 | 4.30E-01 |
| rs8030821 | A | T | -3.37E-02 | 3.90E-01 | 6.00E-03 | 1.54E-08 |  | 4.74E-02 | 3.43E-01 | 2.97E-02 | 1.11E-01 |
| rs848 | A | C | 6.01E-02 | 2.36E-01 | 7.30E-03 | 1.62E-16 |  | -4.04E-03 | 3.69E-01 | 2.93E-02 | 8.90E-01 |
| rs9372120 | T | G | -4.06E-02 | 1.75E-01 | 7.10E-03 | 1.02E-08 |  | -3.15E-02 | 7.64E-01 | 3.31E-02 | 3.41E-01 |
| rs9877752 | A | G | 3.98E-02 | 4.12E-01 | 5.80E-03 | 4.98E-12 |  | -1.82E-02 | 3.84E-01 | 2.90E-02 | 5.31E-01 |

Abbreviation: SNP, single nucleotide polymorphism; EAF, effect allele frequency; SE, standard error.

**Supplementary Table 2. The *F* value of the selected IVs.**

**2.1 ebi-a-GCST005038**

| SNP | effect_allele | other_allele | beta | se | eaf | pval | *F* |
| --- | --- | --- | --- | --- | --- | --- | --- |
| rs62626322 | T | G | -0.067 | 0.009 | 0.097 | 1.35E-13 | 54.858 |
| rs2477923 | T | C | 0.033 | 0.006 | 0.498 | 7.23E-09 | 33.926 |
| rs12413578 | T | C | -0.093 | 0.009 | 0.099 | 3.31E-23 | 98.727 |
| rs10789841 | T | C | 0.046 | 0.006 | 0.31 | 1.48E-12 | 50.766 |
| rs12365699 | A | G | -0.059 | 0.008 | 0.153 | 3.54E-14 | 57.604 |
| rs11033545 | T | G | -0.033 | 0.006 | 0.442 | 2.41E-08 | 30.988 |
| rs12123821 | T | C | 0.116 | 0.014 | 0.048 | 1.04E-16 | 68.535 |
| rs61816766 | T | C | -0.142 | 0.018 | 0.02 | 6.76E-16 | 65.096 |
| rs479844 | A | G | -0.041 | 0.006 | 0.429 | 1.15E-12 | 50.459 |
| rs1214598 | A | G | -0.04 | 0.006 | 0.332 | 2.14E-11 | 44.667 |
| rs7521390 | A | C | -0.036 | 0.006 | 0.284 | 1.40E-08 | 32.291 |
| rs2212434 | T | C | 0.087 | 0.006 | 0.429 | 8.93E-52 | 232.429 |
| rs11236814 | A | T | 0.065 | 0.01 | 0.092 | 3.25E-11 | 44.399 |
| rs6489785 | T | C | 0.043 | 0.006 | 0.369 | 3.57E-13 | 52.624 |
| rs1289273 | A | G | -0.035 | 0.006 | 0.427 | 1.09E-09 | 37.704 |
| rs11168245 | C | G | 0.047 | 0.007 | 0.199 | 1.41E-11 | 45.466 |
| rs760805 | A | T | -0.037 | 0.006 | 0.422 | 3.79E-10 | 38.854 |
| rs1689510 | C | G | 0.051 | 0.006 | 0.316 | 3.39E-17 | 69.901 |
| rs1059513 | T | C | 0.083 | 0.009 | 0.111 | 1.15E-18 | 77.59 |
| rs4943794 | C | G | 0.04 | 0.007 | 0.226 | 1.41E-08 | 32.003 |
| rs1885013 | A | G | -0.038 | 0.006 | 0.359 | 1.33E-09 | 36.766 |
| rs12440045 | A | C | -0.04 | 0.006 | 0.452 | 7.68E-12 | 46.656 |
| rs10519067 | A | G | -0.052 | 0.009 | 0.14 | 1.10E-09 | 37.138 |
| rs56375023 | A | G | 0.073 | 0.007 | 0.213 | 3.11E-27 | 115.563 |
| rs8030821 | A | T | -0.034 | 0.006 | 0.39 | 1.54E-08 | 31.547 |
| rs3540 | A | G | -0.035 | 0.006 | 0.345 | 1.28E-08 | 32.173 |
| rs2241099 | C | G | 0.07 | 0.007 | 0.262 | 4.62E-26 | 111.526 |
| rs3024665 | T | C | 0.067 | 0.012 | 0.077 | 2.18E-08 | 31.228 |
| rs12941864 | T | C | -0.034 | 0.006 | 0.466 | 4.98E-08 | 30.16 |
| rs7224129 | A | G | 0.054 | 0.006 | 0.473 | 8.11E-21 | 88.096 |
| rs11658582 | C | G | -0.056 | 0.006 | 0.434 | 2.07E-21 | 91.057 |
| rs7406234 | T | C | 0.034 | 0.006 | 0.435 | 4.30E-09 | 34.955 |
| rs301802 | A | T | 0.048 | 0.006 | 0.459 | 1.33E-16 | 68.49 |
| rs10414065 | T | C | -0.092 | 0.012 | 0.078 | 2.29E-14 | 58.395 |
| rs12625547 | T | G | 0.043 | 0.008 | 0.214 | 1.98E-08 | 31.476 |
| rs2766678 | A | G | -0.056 | 0.007 | 0.213 | 2.95E-14 | 57.064 |
| rs6011033 | A | G | -0.044 | 0.007 | 0.225 | 2.66E-10 | 40.111 |
| rs10865050 | A | G | -0.125 | 0.008 | 0.153 | 6.37E-50 | 221.089 |
| rs74847330 | A | G | 0.05 | 0.009 | 0.122 | 1.67E-08 | 31.897 |
| rs4973380 | A | T | 0.04 | 0.007 | 0.243 | 2.59E-09 | 35.11 |
| rs5758343 | A | T | 0.047 | 0.007 | 0.218 | 2.17E-11 | 44.57 |
| rs34290285 | A | G | -0.077 | 0.008 | 0.265 | 1.16E-24 | 104.312 |
| rs10174949 | A | G | -0.066 | 0.006 | 0.316 | 9.92E-26 | 108.424 |
| rs7625643 | A | G | -0.035 | 0.006 | 0.442 | 2.96E-09 | 35.587 |
| rs519973 | A | G | 0.036 | 0.006 | 0.354 | 3.23E-09 | 35.601 |
| rs9877752 | A | G | 0.04 | 0.006 | 0.412 | 4.98E-12 | 47.088 |
| rs80064395 | T | C | -0.073 | 0.011 | 0.058 | 1.32E-11 | 46.037 |
| rs6800001 | A | G | 0.033 | 0.006 | 0.342 | 2.28E-08 | 31.284 |
| rs61192126 | T | C | 0.038 | 0.006 | 0.31 | 1.56E-09 | 36 |
| rs228619 | A | G | -0.036 | 0.006 | 0.469 | 2.41E-10 | 40.334 |
| rs58939053 | T | C | 0.054 | 0.006 | 0.344 | 3.85E-19 | 79.531 |
| rs5743618 | A | C | -0.092 | 0.007 | 0.299 | 2.73E-42 | 186.506 |
| rs10033073 | A | G | -0.044 | 0.007 | 0.35 | 1.36E-09 | 36.838 |
| rs72774901 | A | T | 0.112 | 0.011 | 0.082 | 3.92E-25 | 107.929 |
| rs6594499 | A | C | -0.072 | 0.006 | 0.481 | 1.10E-36 | 160.889 |
| rs848 | A | C | 0.06 | 0.007 | 0.236 | 1.62E-16 | 67.78 |
| rs7712601 | T | C | 0.035 | 0.006 | 0.418 | 3.28E-09 | 35.587 |
| rs249677 | A | C | 0.037 | 0.006 | 0.361 | 7.54E-10 | 38.482 |
| rs16903574 | C | G | -0.07 | 0.011 | 0.082 | 1.68E-10 | 40.496 |
| rs2910162 | A | G | -0.034 | 0.006 | 0.316 | 1.54E-08 | 31.802 |
| rs6881706 | T | G | -0.071 | 0.006 | 0.293 | 1.73E-28 | 122.033 |
| rs9372120 | T | G | -0.041 | 0.007 | 0.175 | 1.02E-08 | 32.699 |
| rs1419675 | T | G | -0.039 | 0.007 | 0.242 | 4.72E-08 | 30.25 |
| rs2854001 | A | G | 0.054 | 0.007 | 0.184 | 3.31E-14 | 57.205 |
| rs34004019 | A | G | 0.091 | 0.007 | 0.304 | 2.52E-38 | 165.358 |
| rs3128959 | A | G | -0.06 | 0.01 | 0.124 | 2.25E-10 | 40.022 |
| rs2134814 | C | G | 0.043 | 0.006 | 0.354 | 1.03E-12 | 50.647 |
| rs6461503 | T | C | 0.041 | 0.006 | 0.473 | 8.81E-13 | 51.739 |
| rs17664743 | A | G | 0.041 | 0.007 | 0.204 | 8.27E-09 | 33.347 |
| rs4296977 | T | C | -0.053 | 0.008 | 0.158 | 6.92E-11 | 42.409 |
| rs6990534 | A | G | 0.04 | 0.006 | 0.357 | 1.74E-10 | 40.312 |
| rs2221641 | T | C | -0.042 | 0.006 | 0.388 | 1.03E-12 | 50.434 |
| rs12551834 | A | G | -0.062 | 0.01 | 0.08 | 2.72E-09 | 35.651 |
| rs144829310 | T | G | 0.083 | 0.008 | 0.157 | 2.60E-26 | 112.686 |

Abbreviation: SNP, single nucleotide polymorphism; EAF, effect allele frequency; SE, standard error.

**2.2 ebi-a-GCST90038631**

| SNP | effect_allele | other_allele | beta | se | eaf | pval | F |
| --- | --- | --- | --- | --- | --- | --- | --- |
| rs10020631 | G | A | -0.001 | 0.000 | 0.757 | 1.10E-06 | 23.604 |
| rs115686032 | C | T | -0.004 | 0.001 | 0.988 | 2.20E-06 | 22.336 |
| rs11712086 | T | C | 0.001 | 0.000 | 0.887 | 3.50E-06 | 21.344 |
| rs117181902 | G | A | -0.003 | 0.001 | 0.975 | 3.70E-06 | 21.359 |
| rs13373881 | T | C | -0.001 | 0.000 | 0.896 | 1.10E-06 | 23.652 |
| rs1360768 | C | A | -0.001 | 0.000 | 0.515 | 7.60E-07 | 24.457 |
| rs2924814 | G | C | -0.001 | 0.000 | 0.530 | 1.20E-06 | 23.641 |
| rs3788338 | G | A | -0.001 | 0.000 | 0.740 | 4.90E-06 | 20.877 |
| rs416699 | C | T | -0.001 | 0.000 | 0.268 | 4.20E-06 | 21.185 |
| rs6127099 | A | T | 0.001 | 0.000 | 0.720 | 2.20E-10 | 40.343 |
| rs6794839 | A | G | 0.001 | 0.000 | 0.725 | 1.50E-06 | 23.151 |
| rs71606723 | A | T | -0.001 | 0.000 | 0.779 | 3.70E-07 | 25.841 |
| rs73187207 | G | T | -0.001 | 0.000 | 0.789 | 6.20E-07 | 24.836 |
| rs760900 | A | G | -0.001 | 0.000 | 0.454 | 1.40E-06 | 23.315 |
| rs7740107 | T | A | -0.001 | 0.000 | 0.260 | 6.20E-07 | 24.868 |
| rs77826930 | G | C | -0.004 | 0.001 | 0.986 | 1.90E-06 | 22.671 |
| rs79670018 | C | T | -0.001 | 0.000 | 0.750 | 3.30E-07 | 26.051 |

Abbreviation: SNP, single nucleotide polymorphism; EAF, effect allele frequency; SE, standard error.

**2.3 ebi-a-GCST90038666**

| SNP | effect_allele | other_allele | beta | se | eaf | pval | F |
| --- | --- | --- | --- | --- | --- | --- | --- |
| rs1012791 | C | T | -0.001 | 0.000 | 0.415 | 4.70E-06 | 20.943 |
| rs10849835 | C | T | -0.001 | 0.000 | 0.869 | 7.10E-07 | 24.603 |
| rs115512475 | C | T | -0.002 | 0.000 | 0.971 | 1.70E-06 | 22.957 |
| rs12857519 | G | A | -0.001 | 0.000 | 0.943 | 4.40E-06 | 21.101 |
| rs143600374 | G | A | -0.003 | 0.001 | 0.986 | 1.60E-06 | 23.091 |
| rs4528330 | A | T | 0.001 | 0.000 | 0.622 | 3.90E-06 | 21.297 |
| rs4667636 | G | A | -0.001 | 0.000 | 0.378 | 1.00E-06 | 23.895 |
| rs4896367 | T | C | 0.001 | 0.000 | 0.721 | 4.90E-06 | 20.863 |
| rs66941226 | G | A | -0.001 | 0.000 | 0.910 | 3.90E-06 | 21.317 |
| rs7039739 | C | T | -0.001 | 0.000 | 0.694 | 1.90E-06 | 22.733 |
| rs7192567 | A | T | 0.001 | 0.000 | 0.376 | 9.20E-07 | 24.090 |
| rs73357345 | G | T | -0.003 | 0.001 | 0.981 | 6.00E-07 | 24.917 |
| rs7610701 | T | G | -0.001 | 0.000 | 0.425 | 3.70E-07 | 25.852 |
| rs77841369 | G | A | -0.002 | 0.000 | 0.947 | 3.00E-06 | 21.844 |

Abbreviation: SNP, single nucleotide polymorphism; EAF, effect allele frequency; SE, standard error.

**2.4 ebi-a-GCST90038630**

| SNP | effect_allele | other_allele | beta | se | eaf | pval | F |
| --- | --- | --- | --- | --- | --- | --- | --- |
| rs113279813 | A | T | -0.003 | 0.001 | 0.982 | 4.30E-06 | 21.146 |
| rs113795171 | T | C | -0.002 | 0.000 | 0.973 | 4.00E-06 | 21.288 |
| rs140310769 | T | C | -0.001 | 0.000 | 0.932 | 4.90E-06 | 20.875 |
| rs185944728 | C | T | -0.004 | 0.001 | 0.989 | 3.90E-06 | 21.315 |
| rs192115591 | A | G | -0.003 | 0.001 | 0.981 | 8.50E-07 | 24.233 |
| rs4796835 | T | C | 0.001 | 0.000 | 0.095 | 4.20E-06 | 21.184 |
| rs56249115 | C | T | -0.003 | 0.001 | 0.983 | 4.40E-06 | 21.065 |
| rs62520593 | C | T | -0.001 | 0.000 | 0.918 | 3.40E-07 | 25.997 |
| rs74200897 | T | C | -0.002 | 0.000 | 0.968 | 1.90E-06 | 22.744 |
| rs74475451 | G | A | -0.002 | 0.000 | 0.971 | 2.80E-06 | 21.930 |
| rs76918830 | C | T | -0.002 | 0.000 | 0.972 | 6.30E-07 | 24.805 |
| rs77935812 | G | A | -0.002 | 0.000 | 0.954 | 1.00E-06 | 23.888 |
| rs907433 | G | A | 0.001 | 0.000 | 0.124 | 4.80E-06 | 20.933 |

Abbreviation: SNP, single nucleotide polymorphism; EAF, effect allele frequency; SE, standard error.

**2.5 finn-b-N14_CYSTKID**

| SNP | effect_allele | other_allele | beta | se | eaf | pval | F |
| --- | --- | --- | --- | --- | --- | --- | --- |
| rs112905364 | A | G | 0.401 | 0.087 | 0.032 | 3.53E-06 | 21.504 |
| rs7752324 | A | G | 0.194 | 0.042 | 0.200 | 3.06E-06 | 21.781 |
| rs78999450 | G | C | 0.350 | 0.076 | 0.043 | 4.44E-06 | 21.064 |

Abbreviation: SNP, single nucleotide polymorphism; EAF, effect allele frequency; SE, standard error.

**2.6 finn-b-N14_RENFAILNAS**

| SNP | effect_allele | other_allele | beta | se | eaf | pval | F |
| --- | --- | --- | --- | --- | --- | --- | --- |
| rs252566 | T | C | -0.160 | 0.033 | 0.780 | 1.10E-06 | 23.747 |
| rs2928963 | T | C | 0.173 | 0.036 | 0.792 | 1.28E-06 | 23.448 |
| rs56359522 | G | T | -0.191 | 0.041 | 0.152 | 3.57E-06 | 21.483 |
| rs62481228 | T | C | -0.141 | 0.031 | 0.311 | 4.60E-06 | 20.998 |
| rs73522232 | C | G | 0.302 | 0.065 | 0.042 | 3.52E-06 | 21.509 |
| rs74859638 | A | C | -0.190 | 0.040 | 0.159 | 2.24E-06 | 22.382 |
| rs7796654 | A | G | 0.153 | 0.033 | 0.753 | 4.69E-06 | 20.962 |
| rs852587 | T | A | -0.137 | 0.028 | 0.542 | 1.42E-06 | 23.247 |

Abbreviation: SNP, single nucleotide polymorphism; EAF, effect allele frequency; SE, standard error.

**Supplementary Table 3. Mendelian randomization analysis of kidney diseases and allergic diseases.**

**3.1 Mendelian randomization analysis of kidney stone, ureter stone or bladder stone and allergic diseases.(p<5e-6)**

| **Exposure:ebi-a-GCST90038631** | | | exposure | | | |  | outcome | | | |
| --- | --- | --- | --- | --- | --- | --- | --- | --- | --- | --- | --- |
| SNP | effect_allele | other_allele | beta | eaf | se | pval |  | beta | eaf | se | pval |
| rs10020631 | G | A | -1.02E-03 | 7.57E-01 | 2.09E-04 | 1.10E-06 |  | -5.80E-03 | 8.11E-01 | 7.30E-03 | 4.31E-01 |
| rs115686032 | C | T | -3.90E-03 | 9.88E-01 | 8.26E-04 | 2.20E-06 |  | 2.74E-02 | 9.68E-01 | 2.68E-02 | 3.07E-01 |
| rs11712086 | T | C | 1.32E-03 | 8.87E-01 | 2.86E-04 | 3.50E-06 |  | 3.90E-03 | 1.05E-01 | 9.30E-03 | 6.72E-01 |
| rs117181902 | G | A | -2.72E-03 | 9.75E-01 | 5.88E-04 | 3.70E-06 |  | -3.30E-03 | 9.57E-01 | 1.83E-02 | 8.59E-01 |
| rs13373881 | T | C | -1.42E-03 | 8.96E-01 | 2.92E-04 | 1.10E-06 |  | -8.50E-03 | 6.63E-02 | 9.60E-03 | 3.78E-01 |
| rs1360768 | C | A | -8.84E-04 | 5.15E-01 | 1.79E-04 | 7.60E-07 |  | 7.00E-04 | 5.03E-01 | 5.70E-03 | 9.01E-01 |
| rs2924814 | G | C | -8.70E-04 | 5.30E-01 | 1.79E-04 | 1.20E-06 |  | -1.30E-03 | 5.58E-01 | 5.80E-03 | 8.21E-01 |
| rs3788338 | G | A | -9.27E-04 | 7.40E-01 | 2.03E-04 | 4.90E-06 |  | 1.37E-02 | 7.38E-01 | 6.50E-03 | 3.51E-02 |
| rs416699 | C | T | -9.36E-04 | 2.68E-01 | 2.03E-04 | 4.20E-06 |  | 8.60E-03 | 7.30E-01 | 6.60E-03 | 1.93E-01 |
| rs6127099 | A | T | 1.29E-03 | 7.20E-01 | 2.03E-04 | 2.20E-10 |  | 4.10E-03 | 6.65E-01 | 6.60E-03 | 5.36E-01 |
| rs71606723 | A | T | -1.09E-03 | 7.79E-01 | 2.15E-04 | 3.70E-07 |  | 1.10E-03 | 7.18E-01 | 6.90E-03 | 8.75E-01 |
| rs73187207 | G | T | -1.09E-03 | 7.89E-01 | 2.19E-04 | 6.20E-07 |  | 1.12E-02 | 8.04E-01 | 7.00E-03 | 1.12E-01 |
| rs760900 | A | G | -8.69E-04 | 4.54E-01 | 1.80E-04 | 1.40E-06 |  | 1.60E-03 | 4.52E-01 | 5.70E-03 | 7.83E-01 |
| rs7740107 | T | A | -1.01E-03 | 2.60E-01 | 2.03E-04 | 6.20E-07 |  | -6.00E-03 | 2.79E-01 | 6.50E-03 | 3.53E-01 |
| rs77826930 | G | C | -3.62E-03 | 9.86E-01 | 7.60E-04 | 1.90E-06 |  | -2.33E-02 | 9.88E-01 | 3.19E-02 | 4.64E-01 |
| rs79670018 | C | T | -1.10E-03 | 7.50E-01 | 2.16E-04 | 3.30E-07 |  | 7.10E-03 | 7.82E-01 | 7.30E-03 | 3.33E-01 |
| Abbreviation: SNP, single nucleotide polymorphism; EAF, effect allele frequency; SE, standard error.  **3.2 Mendelian randomization analysis of other renal or kidney problem and allergic diseases.(p<5e-6)** | | | | | | | | | | | |
| **Exposure:ebi-a-GCST90038666** | | | exposure | | | |  | outcome | | | |
| SNP | effect_allele | other_allele | beta | eaf | se | pval |  | beta | eaf | se | pval |
| rs1012791 | C | T | -6.98E-04 | 4.15E-01 | 1.53E-04 | 4.70E-06 |  | 5.30E-03 | 5.37E-01 | 5.80E-03 | 3.65E-01 |
| rs10849835 | C | T | -1.10E-03 | 8.69E-01 | 2.21E-04 | 7.10E-07 |  | -1.83E-02 | 8.16E-01 | 8.30E-03 | 2.79E-02 |
| rs115512475 | C | T | -2.19E-03 | 9.71E-01 | 4.57E-04 | 1.70E-06 |  | -1.18E-02 | 9.71E-01 | 1.71E-02 | 4.90E-01 |
| rs12857519 | G | A | -1.49E-03 | 9.43E-01 | 3.24E-04 | 4.40E-06 |  | 1.27E-02 | 9.57E-01 | 1.28E-02 | 3.20E-01 |
| rs143600374 | G | A | -3.08E-03 | 9.86E-01 | 6.40E-04 | 1.60E-06 |  | -2.90E-03 | 9.90E-01 | 2.56E-02 | 9.09E-01 |
| rs4528330 | A | T | 7.18E-04 | 6.22E-01 | 1.56E-04 | 3.90E-06 |  | 8.40E-03 | 6.94E-01 | 6.00E-03 | 1.57E-01 |
| rs4667636 | G | A | -7.59E-04 | 3.78E-01 | 1.55E-04 | 1.00E-06 |  | -5.90E-03 | 6.21E-01 | 5.90E-03 | 3.17E-01 |
| rs4896367 | T | C | 7.94E-04 | 7.21E-01 | 1.74E-04 | 4.90E-06 |  | 4.90E-03 | 2.62E-01 | 6.80E-03 | 4.68E-01 |
| rs66941226 | G | A | -1.21E-03 | 9.10E-01 | 2.63E-04 | 3.90E-06 |  | -1.74E-02 | 8.98E-01 | 1.00E-02 | 8.25E-02 |
| rs7039739 | C | T | -7.86E-04 | 6.94E-01 | 1.65E-04 | 1.90E-06 |  | 7.30E-03 | 6.57E-01 | 6.30E-03 | 2.50E-01 |
| rs7192567 | A | T | 7.64E-04 | 3.76E-01 | 1.56E-04 | 9.20E-07 |  | 9.50E-03 | 3.74E-01 | 6.00E-03 | 1.14E-01 |
| rs73357345 | G | T | -2.81E-03 | 9.81E-01 | 5.63E-04 | 6.00E-07 |  | 4.14E-02 | 9.83E-01 | 2.73E-02 | 1.28E-01 |
| rs7610701 | T | G | -8.35E-04 | 4.25E-01 | 1.64E-04 | 3.70E-07 |  | 3.60E-03 | 3.95E-01 | 6.60E-03 | 5.83E-01 |
| rs77841369 | G | A | -1.57E-03 | 9.47E-01 | 3.35E-04 | 3.00E-06 |  | 5.90E-03 | 9.52E-01 | 1.31E-02 | 6.51E-01 |
| Abbreviation: SNP, single nucleotide polymorphism; EAF, effect allele frequency; SE, standard error.  **3.3 Mendelian randomization analysis of urinary tract or kidney infection and allergic diseases.(p<5e-6)** | | | | | | | | | | | |
| **Exposure:ebi-a-GCST90038630** | | | exposure | | | |  | outcome | | | |
| SNP | effect_allele | other_allele | beta | eaf | se | pval |  | beta | eaf | se | pval |
| rs113279813 | A | T | -2.62E-03 | 9.82E-01 | 5.71E-04 | 4.30E-06 |  | 9.00E-03 | 9.85E-01 | 2.11E-02 | 6.71E-01 |
| rs113795171 | T | C | -2.21E-03 | 9.73E-01 | 4.78E-04 | 4.00E-06 |  | -2.10E-02 | 2.89E-02 | 1.85E-02 | 2.56E-01 |
| rs140310769 | T | C | -1.38E-03 | 9.32E-01 | 3.01E-04 | 4.90E-06 |  | -8.50E-03 | 5.78E-02 | 1.15E-02 | 4.61E-01 |
| rs185944728 | C | T | -3.57E-03 | 9.89E-01 | 7.73E-04 | 3.90E-06 |  | 3.50E-02 | 9.81E-01 | 2.86E-02 | 2.21E-01 |
| rs192115591 | A | G | -2.97E-03 | 9.81E-01 | 6.04E-04 | 8.50E-07 |  | 2.16E-02 | 3.23E-02 | 2.41E-02 | 3.70E-01 |
| rs4796835 | T | C | 1.20E-03 | 9.51E-02 | 2.60E-04 | 4.20E-06 |  | 6.30E-03 | 6.97E-02 | 1.03E-02 | 5.44E-01 |
| rs56249115 | C | T | -2.68E-03 | 9.83E-01 | 5.84E-04 | 4.40E-06 |  | -3.80E-02 | 9.86E-01 | 2.37E-02 | 1.09E-01 |
| rs62520593 | C | T | -1.41E-03 | 9.18E-01 | 2.77E-04 | 3.40E-07 |  | -4.30E-03 | 9.20E-01 | 1.04E-02 | 6.77E-01 |
| rs74200897 | T | C | -2.30E-03 | 9.68E-01 | 4.83E-04 | 1.90E-06 |  | 2.50E-03 | 5.44E-02 | 1.62E-02 | 8.79E-01 |
| rs74475451 | G | A | -2.11E-03 | 9.71E-01 | 4.50E-04 | 2.80E-06 |  | -1.87E-02 | 9.68E-01 | 1.69E-02 | 2.68E-01 |
| rs76918830 | C | T | -2.35E-03 | 9.72E-01 | 4.71E-04 | 6.30E-07 |  | 1.68E-02 | 9.59E-01 | 1.81E-02 | 3.54E-01 |
| rs77935812 | G | A | -1.76E-03 | 9.54E-01 | 3.61E-04 | 1.00E-06 |  | -2.06E-02 | 9.44E-01 | 1.39E-02 | 1.38E-01 |
| rs907433 | G | A | 1.06E-03 | 1.24E-01 | 2.31E-04 | 4.80E-06 |  | -2.90E-03 | 9.27E-01 | 8.70E-03 | 7.42E-01 |
| Abbreviation: SNP, single nucleotide polymorphism; EAF, effect allele frequency; SE, standard error.  **3.4 Mendelian randomization analysis of cyst of kidney and allergic diseases.(p<5e-6)** | | | | | | | | | | | |
| **Exposure:finn-b-N14_CYSTKID** | | | exposure | | | |  | outcome | | | |
| SNP | effect_allele | other_allele | beta | eaf | se | pval |  | beta | eaf | se | pval |
| rs112905364 | A | G | 4.01E-01 | 3.17E-02 | 8.66E-02 | 3.53E-06 |  | 4.50E-03 | 3.57E-02 | 1.52E-02 | 7.66E-01 |
| rs7752324 | A | G | 1.94E-01 | 2.00E-01 | 4.15E-02 | 3.06E-06 |  | -2.18E-02 | 2.04E-01 | 7.00E-03 | 1.88E-03 |
| rs78999450 | G | C | 3.50E-01 | 4.29E-02 | 7.63E-02 | 4.44E-06 |  | -2.15E-02 | 3.40E-02 | 1.27E-02 | 9.01E-02 |
| Abbreviation: SNP, single nucleotide polymorphism; EAF, effect allele frequency; SE, standard error.  **3.5 Mendelian randomization analysis of unspecified kidney failure and allergic diseases.(p<5e-6)** | | | | | | | | | | | |
| **Exposure:finn-b-N14_RENFAILNAS** | | | exposure | | | |  | outcome | | | |
| SNP | effect_allele | other_allele | beta | eaf | se | pval |  | beta | eaf | se | pval |
| rs252566 | T | C | -1.60E-01 | 7.80E-01 | 3.27E-02 | 1.10E-06 |  | -1.04E-02 | 2.36E-01 | 7.00E-03 | 1.36E-01 |
| rs2928963 | T | C | 1.73E-01 | 7.92E-01 | 3.58E-02 | 1.28E-06 |  | -6.00E-04 | 2.25E-01 | 7.00E-03 | 9.35E-01 |
| rs56359522 | G | T | -1.91E-01 | 1.52E-01 | 4.12E-02 | 3.57E-06 |  | 1.21E-02 | 8.21E-01 | 8.30E-03 | 1.43E-01 |
| rs62481228 | T | C | -1.41E-01 | 3.11E-01 | 3.07E-02 | 4.60E-06 |  | -7.40E-03 | 3.55E-01 | 5.90E-03 | 2.14E-01 |
| rs73522232 | C | G | 3.02E-01 | 4.24E-02 | 6.51E-02 | 3.52E-06 |  | -1.68E-02 | 4.42E-02 | 1.57E-02 | 2.83E-01 |
| rs74859638 | A | C | -1.90E-01 | 1.59E-01 | 4.01E-02 | 2.24E-06 |  | 2.00E-04 | 1.16E-01 | 1.08E-02 | 9.87E-01 |
| rs7796654 | A | G | 1.53E-01 | 7.53E-01 | 3.33E-02 | 4.69E-06 |  | -4.60E-03 | 2.47E-01 | 6.80E-03 | 5.02E-01 |

Abbreviation: SNP, single nucleotide polymorphism; EAF, effect allele frequency; SE, standard error.

**3.6 Mendelian randomization analysis of kidney stone, ureter stone or bladder stone and allergic diseases.(p<5e-8)**

| **Exposure:ebi-a-GCST90038631** | | | exposure | | | |  | outcome | | | |
| --- | --- | --- | --- | --- | --- | --- | --- | --- | --- | --- | --- |
| SNP | effect_allele | other_allele | beta | eaf | se | pval |  | beta | eaf | se | pval |
| rs116245586 | A | G | -3.05E-03 | 9.65E-01 | 4.93E-04 | 6.30E-10 |  | -4.07E-02 | 3.74E-02 | 1.59E-02 | 1.04E-02 |
| rs6127099 | A | T | 1.29E-03 | 7.20E-01 | 2.03E-04 | 2.20E-10 |  | 4.10E-03 | 6.65E-01 | 6.60E-03 | 5.36E-01 |
| Abbreviation: SNP, single nucleotide polymorphism; EAF, effect allele frequency; SE, standard error. | | | | | | | | | | | |

**Supplementary Table 4.**

**4.1 Causality of 7 kidney diseases (p<5e-6) and allergic diseases.**

| **exposure** | **Methods** | **nSNP** | **OR(95%CI)** | **P_value** | **Q_pval** | | **P-Egger_intercept** |
| --- | --- | --- | --- | --- | --- | --- | --- |
| **ebi-a-GCST90038631** | **Case/Control:3,725/480,873** |  |  |  |  |  | |
|  | MR Egger | 16 | 37.702(7.12E-04 - 2.00E+06) | 0.524 | 0.479 | 0.330 | |
|  | Weighted median | 16 | 0.429(4.53E-03 - 4.06E+01) | 0.716 |  |  | |
|  | Inverse variance weighted | 16 | 0.180(6.93E-03 - 4.67E+00) | 0.302 |  |  | |
|  | Simple mode | 16 | 21.353(3.85E-03 - 1.18E+05) | 0.497 |  |  | |
|  | Weighted mode | 16 | 11.979(3.44E-03 - 4.17E+04) | 0.560 |  |  | |
| **ebi-a-GCST90038666** | **Case/Control:2,609/481,989** |  |  |  |  |  | |
|  | MR Egger | 14 | 0.132(6.91E-08 - 2.54E+05) | 0.789 | 0.125 | 0.516 | |
|  | Weighted median | 14 | 42.425(7.45E-02 - 2.42E+04) | 0.247 |  |  | |
|  | Inverse variance weighted | 14 | 13.207(7.39E-02 - 2.36E+03) | 0.329 |  |  | |
|  | Simple mode | 14 | 10264.487(9.04E-03 - 1.17E+10) | 0.217 |  |  | |
|  | Weighted mode | 14 | 6245.481(1.03E-02 - 3.78E+09) | 0.221 |  |  | |
| **ebi-a-GCST90038630** | **Case/Control:2,691/481,907** |  |  |  |  |  | |
|  | MR Egger | 13 | 0.034(1.09E-07 - 1.06E+04) | 0.611 | 0.511 | 0.414 | |
|  | Weighted median | 13 | 4.588(1.21E-02 - 1.74E+03) | 0.615 |  |  | |
|  | Inverse variance weighted | 13 | 5.875(7.58E-02 - 4.55E+02) | 0.425 |  |  | |
|  | Simple mode | 13 | 1435.311(1.44E-02 - 1.44E+08) | 0.240 |  |  | |
|  | Weighted mode | 13 | 0.05(8.77E-07 - 2.86E+03) | 0.602 |  |  | |
| **finn-b-N14-CYSTKID** | **Case/Control:739/217,185** |  |  |  |  |  | |
|  | MR Egger | 3 | 1.078(9.30E-01 - 1.25E+00) | 0.502 | 0.060 | 0.318 | |
|  | Weighted median | 3 | 0.949(8.91E-01 - 1.01E+00) | 0.099 |  |  | |
|  | Inverse variance weighted | 3 | 0.945(8.82E-01 - 1.01E+00) | 0.113 |  |  | |
|  | Simple mode | 3 | 0.928(8.52E-01 - 1.01E+00) | 0.228 |  |  | |
|  | Weighted mode | 3 | 0.939(8.57E-01 - 1.03E+00) | 0.304 |  |  | |
| **finn-b-N14-RENFAILNAS** | **Case/Control:963/212,841** |  |  |  |  |  | |
|  | MR Egger | 7 | 0.843(7.01E-01 - 1.01E+00) | 0.130 | 0.277 | 0.129 | |
|  | Weighted median | 7 | 0.996(9.51E-01 - 1.04E+00) | 0.874 |  |  | |
|  | Inverse variance weighted | 7 | 0.998(9.61E-01 - 1.04E+00) | 0.903 |  |  | |
|  | Simple mode | 7 | 0.974(8.98E-01 - 1.06E+00) | 0.538 |  |  | |
|  | Weighted mode | 7 | 0.977(9.04E-01 - 1.05E+00) | 0.566 |  |  | |

**4.2 Causality of 7 kidney diseases (p<5e-8) and allergic diseases.**

| **exposure** | **Methods** | **nSNP** | **OR(95%CI)** | **P_value** | **Q_pval** |
| --- | --- | --- | --- | --- | --- |
| **ebi-a-GCST90038631** | **Case/Control:3,725/480,873** |  |  |  |  |
|  | Inverse variance weighted | 2 | 3477.409(1.64E-01 - 7.35E+07) | 0.109 | 0.164 |

## Supplementary Figures

1.
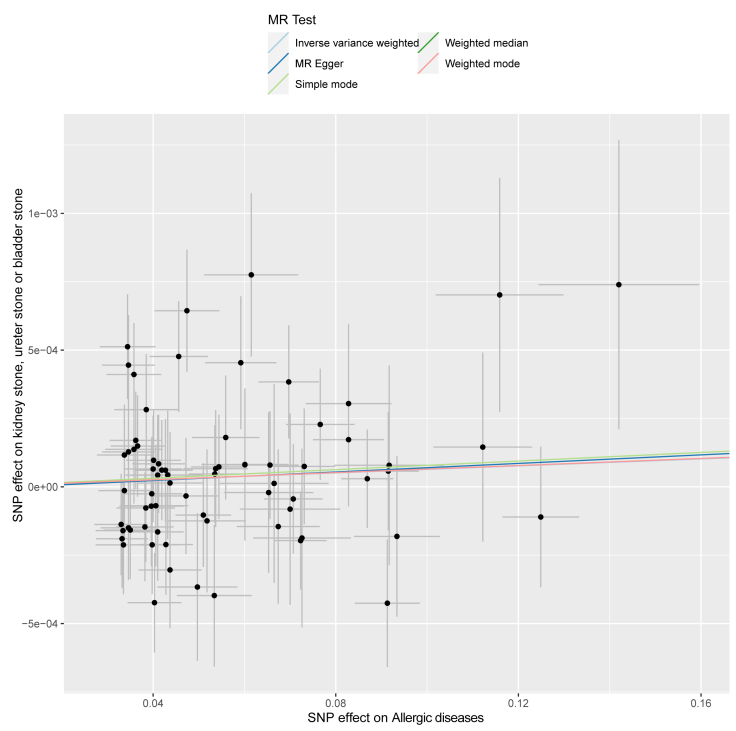
(b)
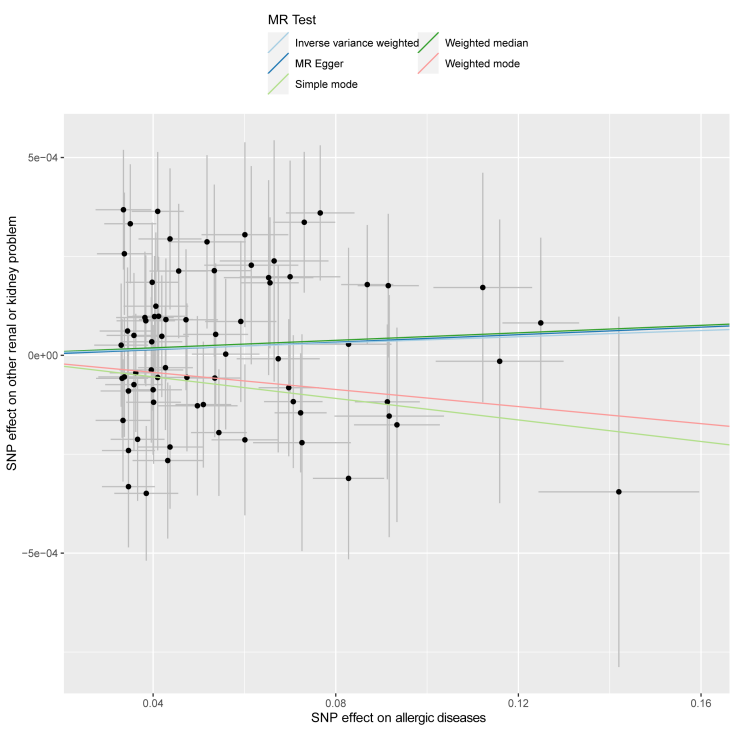


(c)
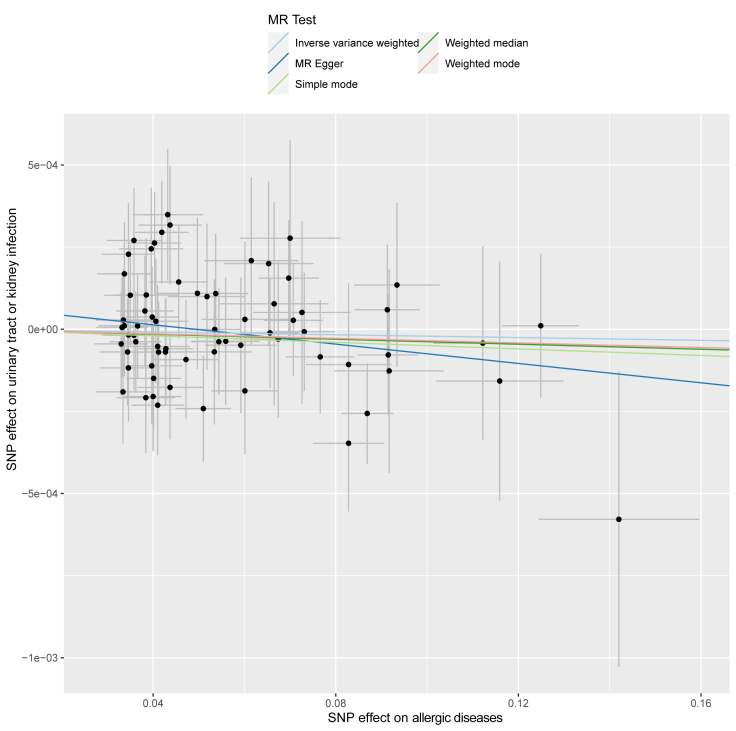
(d)
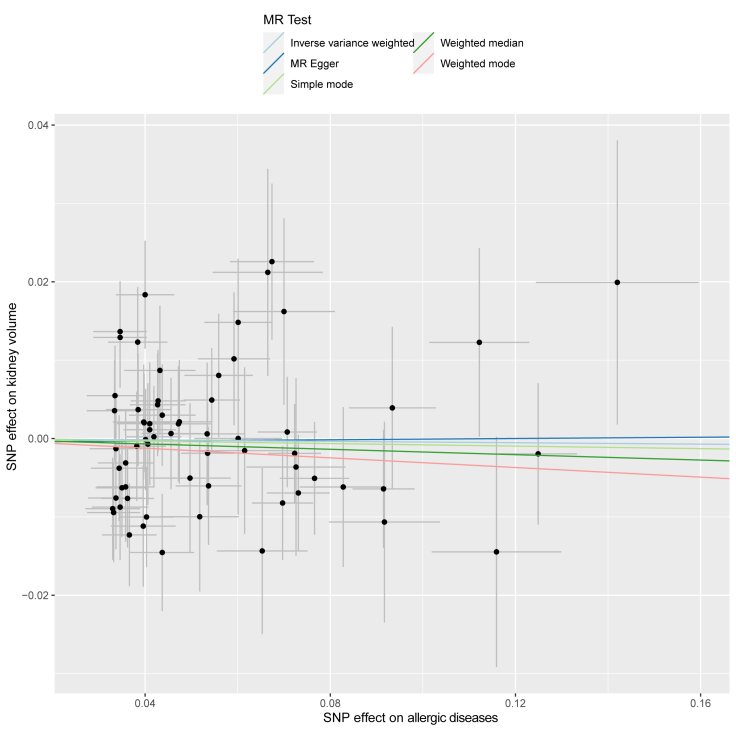


(e)
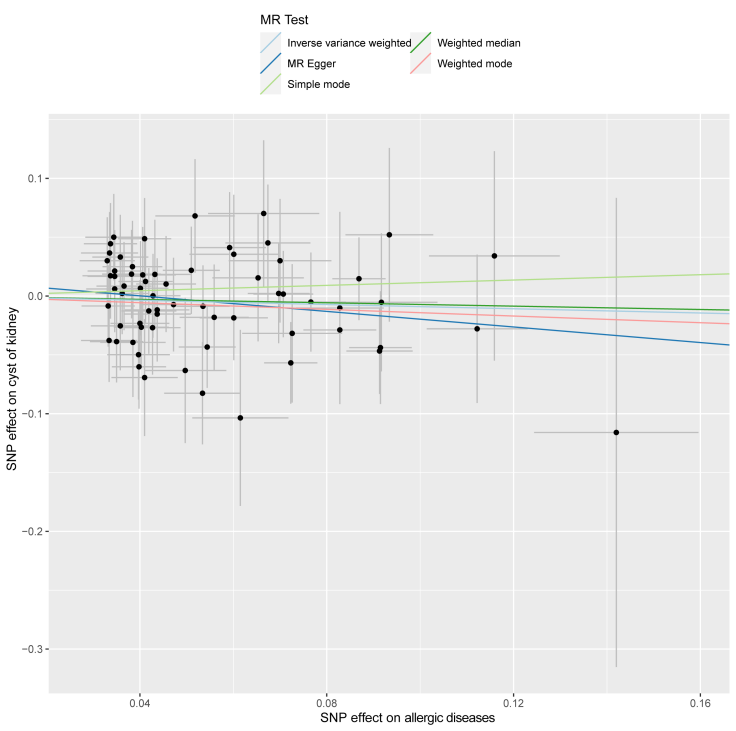
(f)
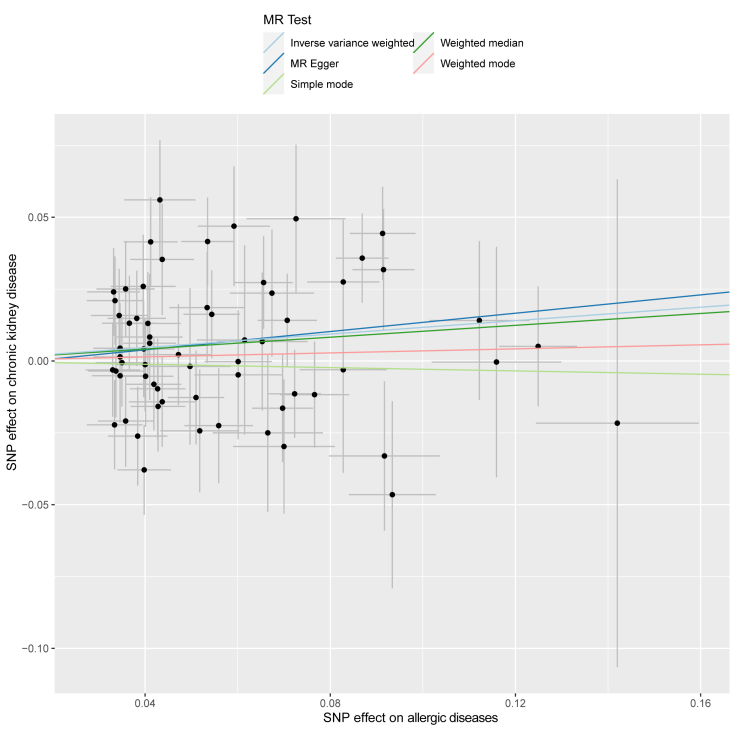


(g)
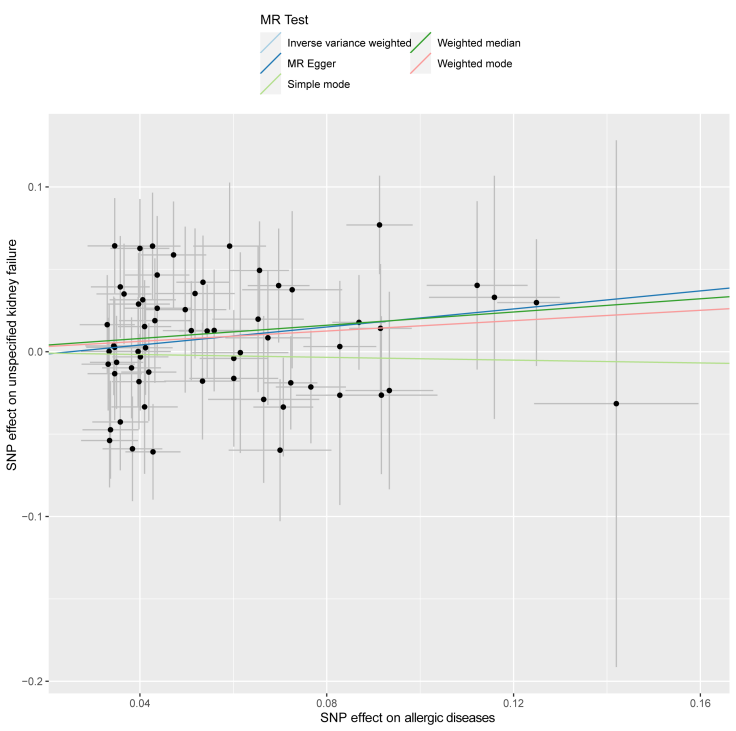


**Supplementary Figure 1** scatter plot: (a) Allergic diseases and kidney stone, ureter stone or bladder stone; (b) Allergic diseases and other renal or kidney problem; (c) Allergic diseases and urinary tract or kidney infection; (d) Allergic diseases and kidney volume; (e) Allergic diseases and cyst of kidney; (f) Allergic diseases and chronic kidney disease; (g) Allergic diseases and unspecified kidney failure.

1. **
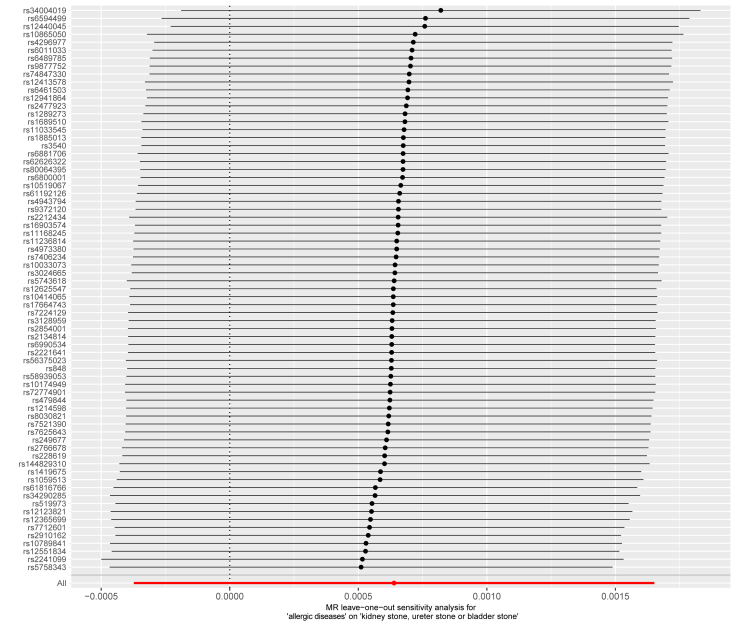
(b)
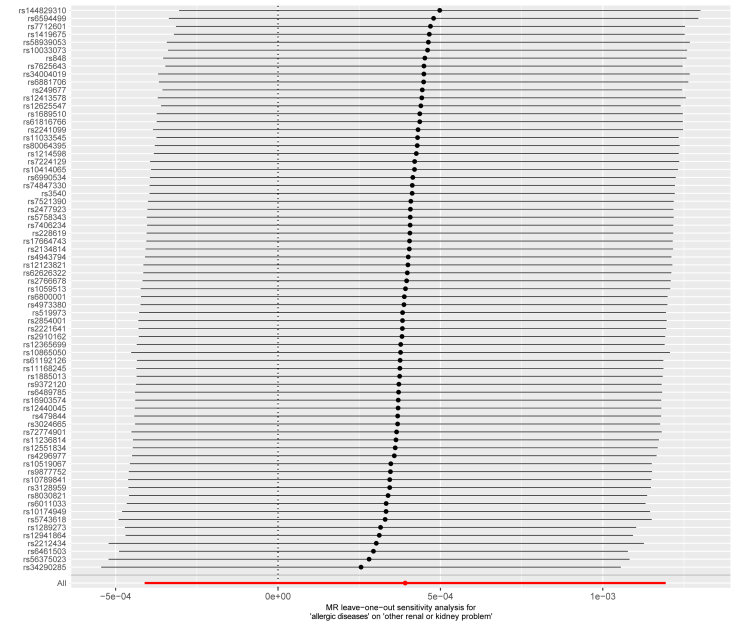
**

**(c)
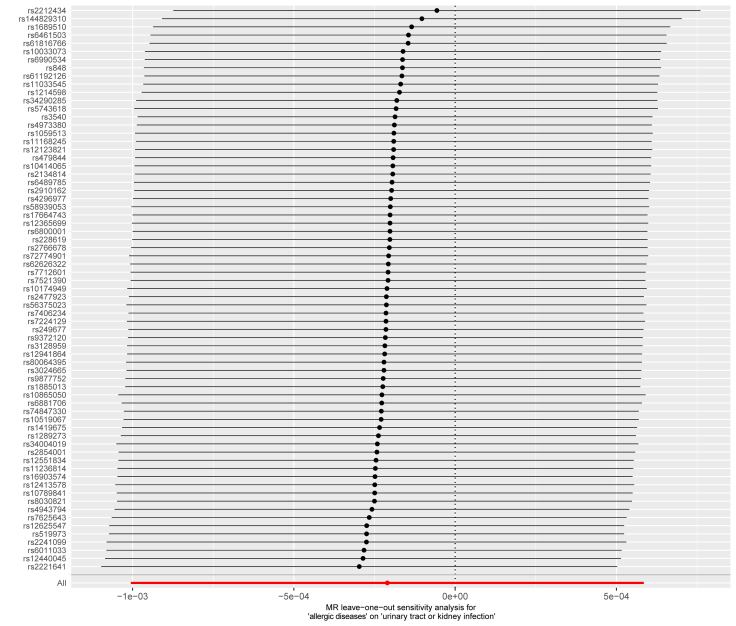
(d)
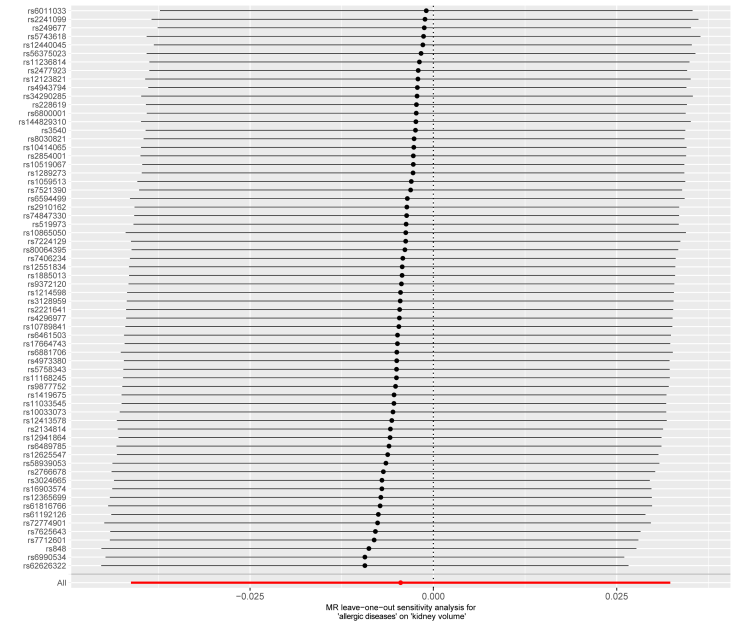
**

**(e)
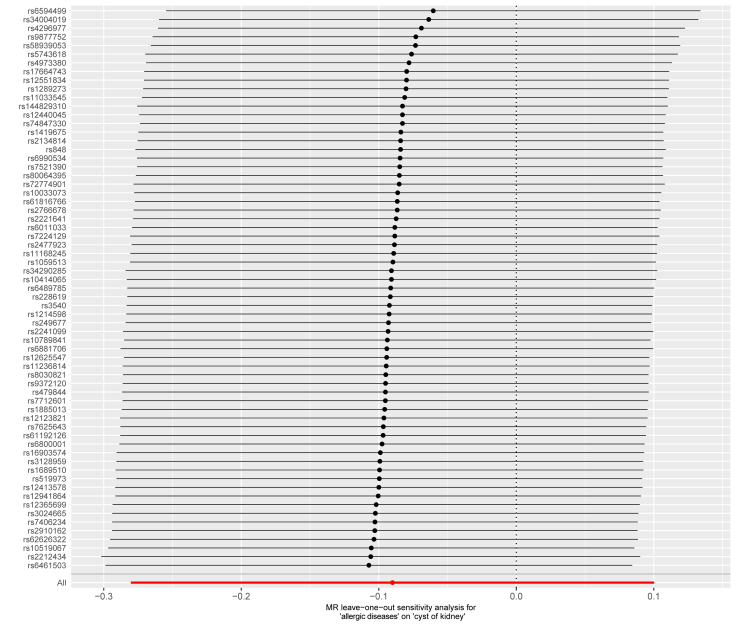
(f)
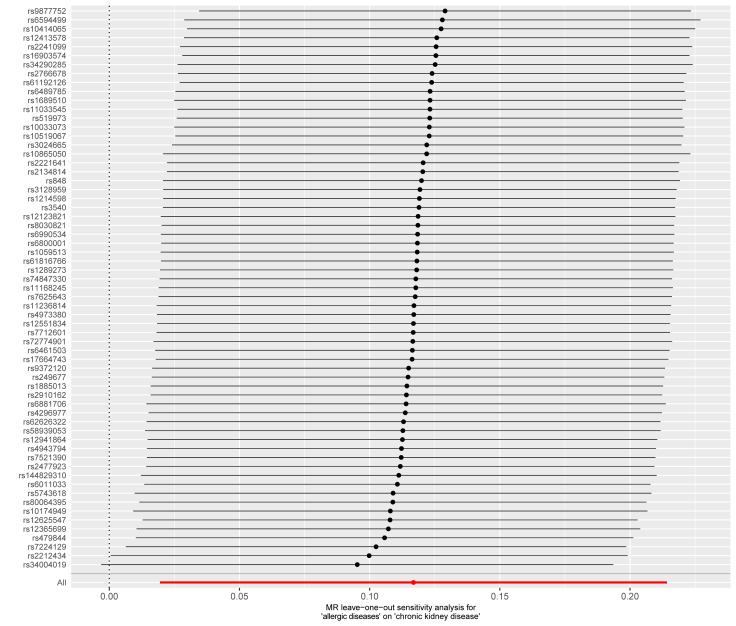
**

**(g)
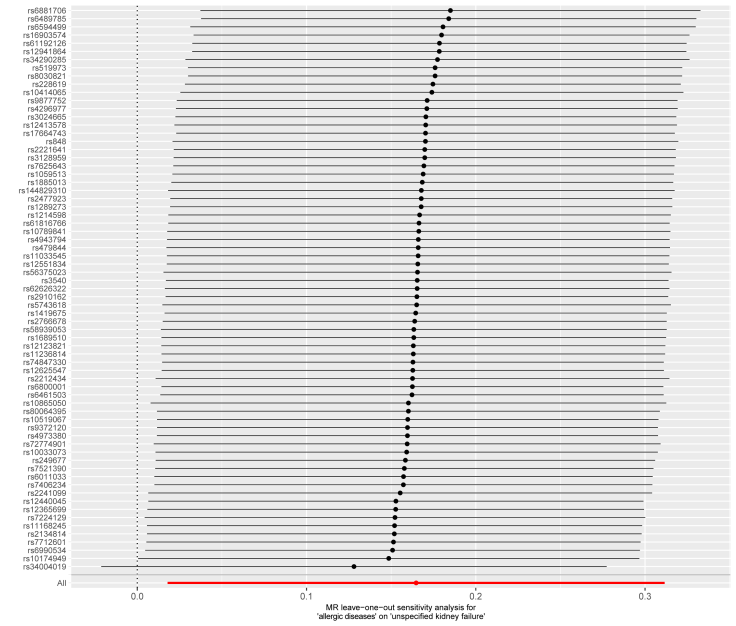
**

**Supplementary Figure 2** Leave-one-out sensitivity analysis: (a) Allergic diseases and kidney stone, ureter stone or bladder stone; (b) Allergic diseases and other renal or kidney problem; (c) Allergic diseases and urinary tract or kidney infection; (d) Allergic diseases and kidney volume; (e) Allergic diseases and cyst of kidney; (f) Allergic diseases and chronic kidney disease; (g) Allergic diseases and unspecified kidney failure.

1. **
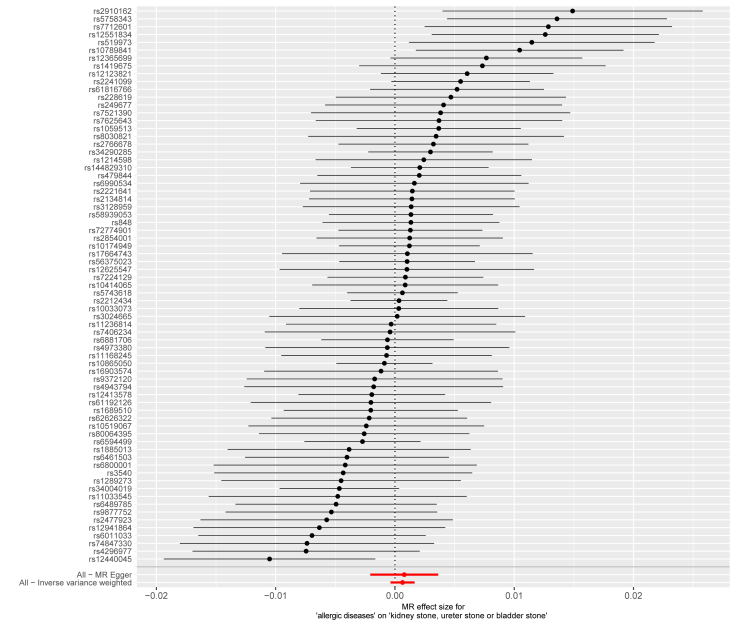
(b)
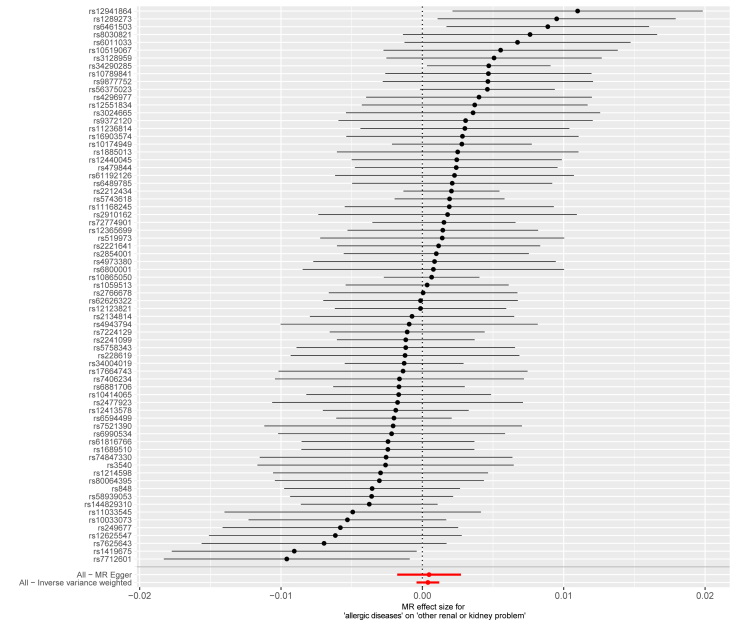
**

**(c)
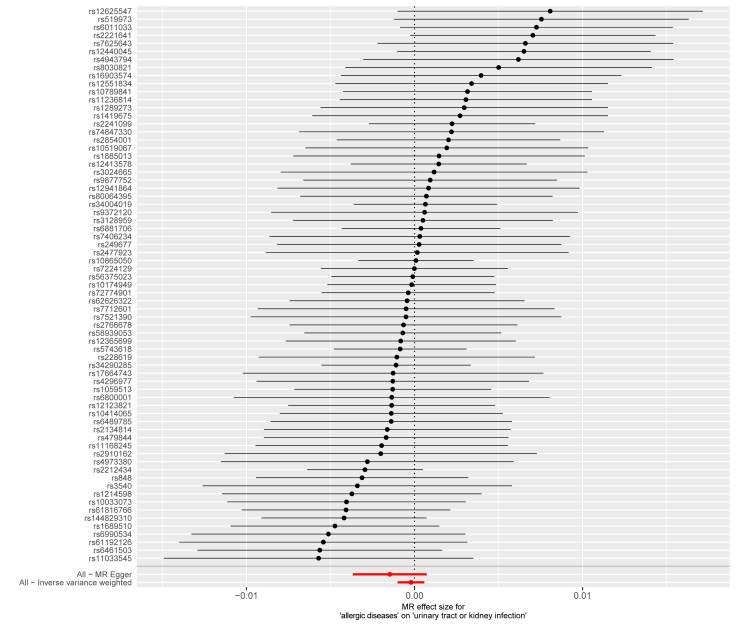
(d)
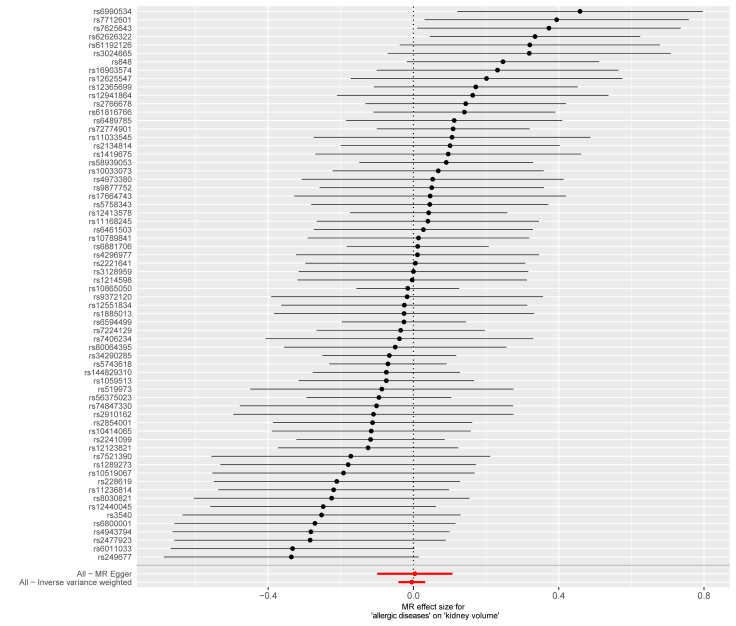
**

**(e)
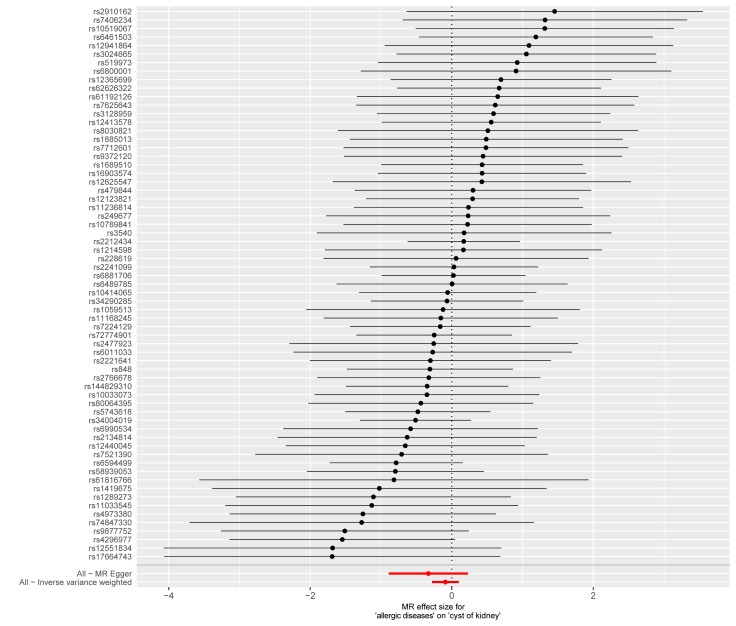
(f)
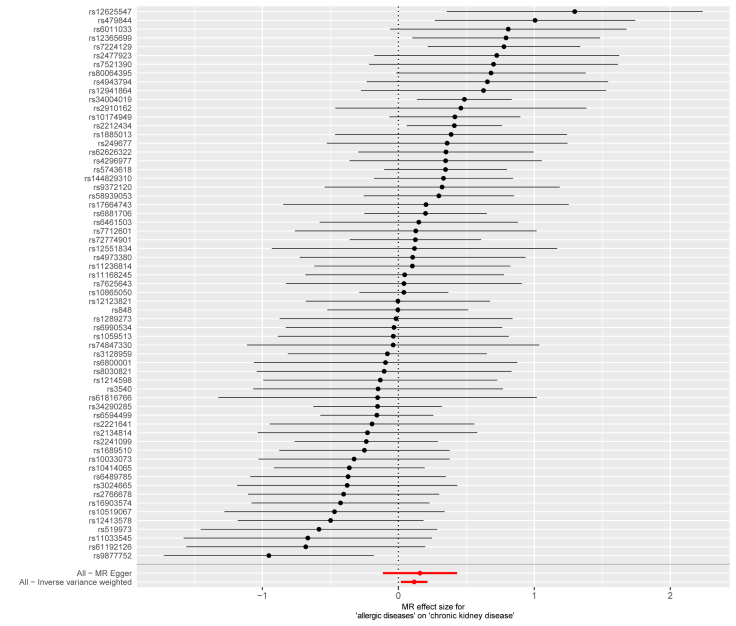
**

**(g)
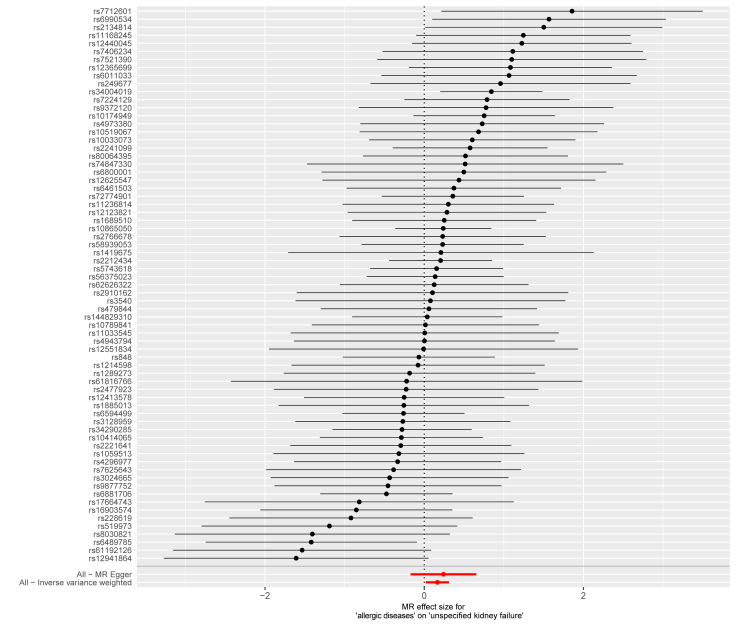
**

**Supplementary Figure 3** Forest plot: (a) Allergic diseases and kidney stone, ureter stone or bladder stone; (b) Allergic diseases and other renal or kidney problem; (c) Allergic diseases and urinary tract or kidney infection; (d) Allergic diseases and kidney volume; (e) Allergic diseases and cyst of kidney; (f) Allergic diseases and chronic kidney disease; (g) Allergic diseases and unspecified kidney failure.

1. **
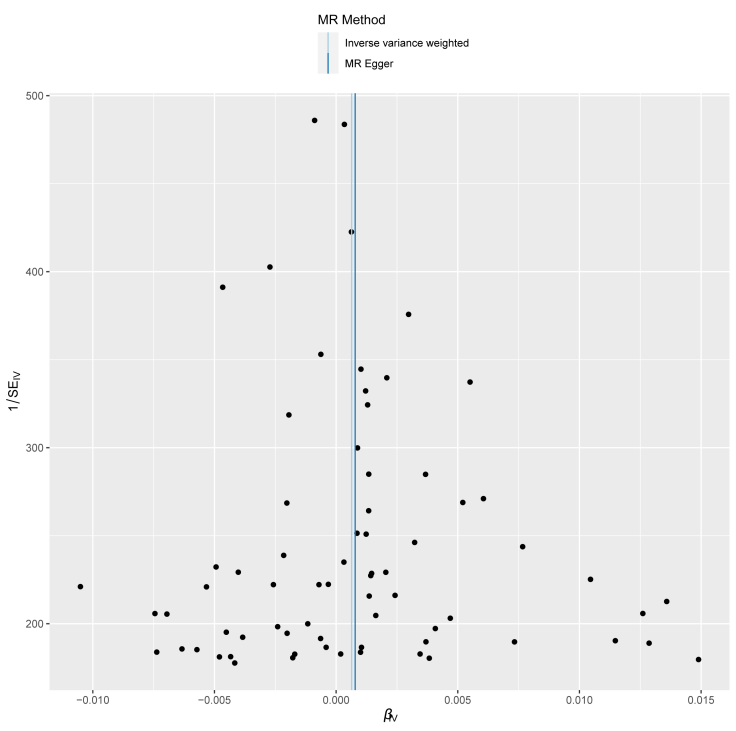
(b)
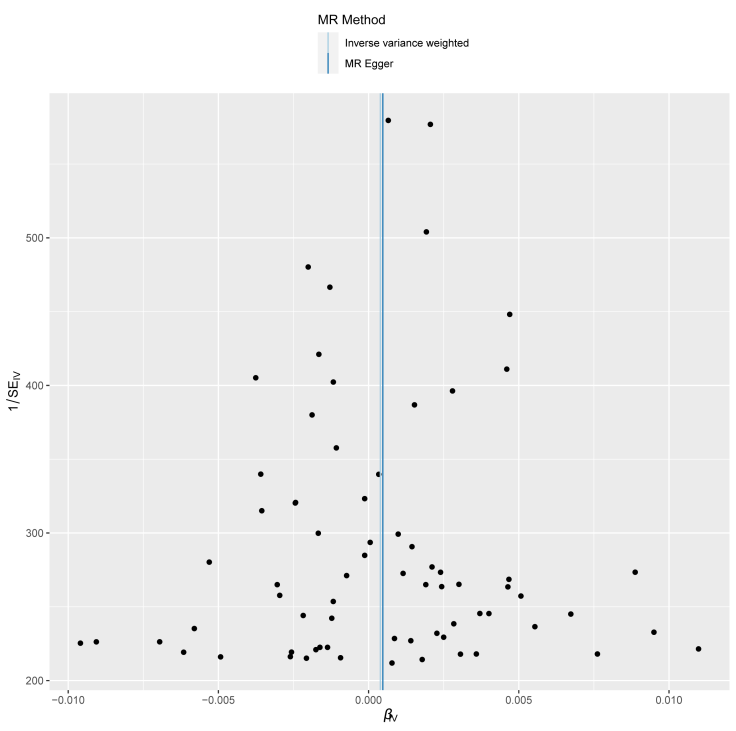
**

**(c)
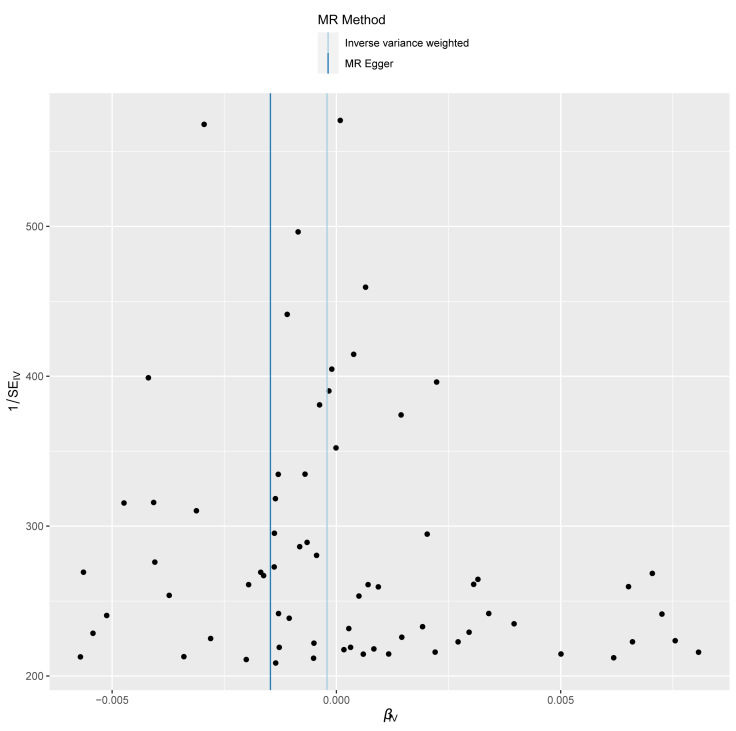
(d)
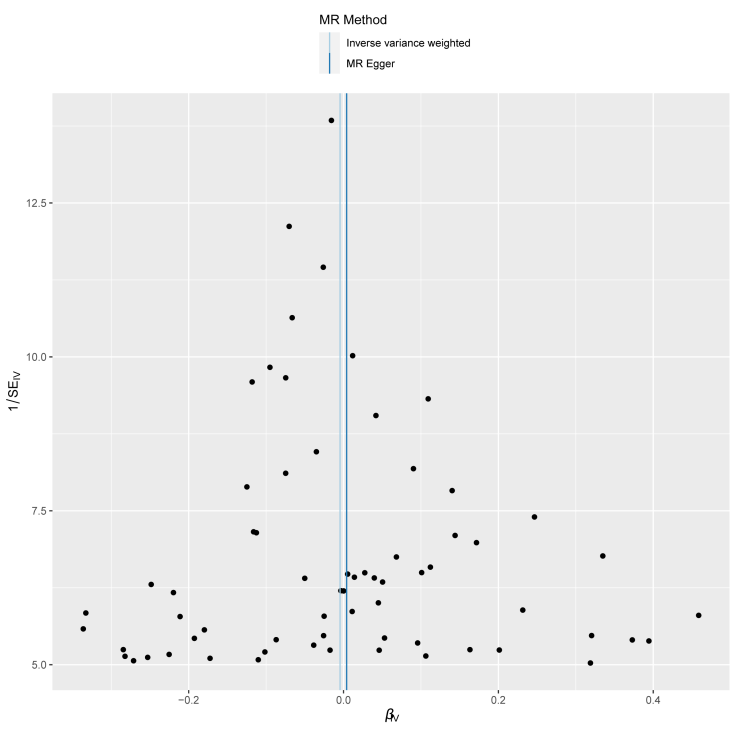
**

**(e)
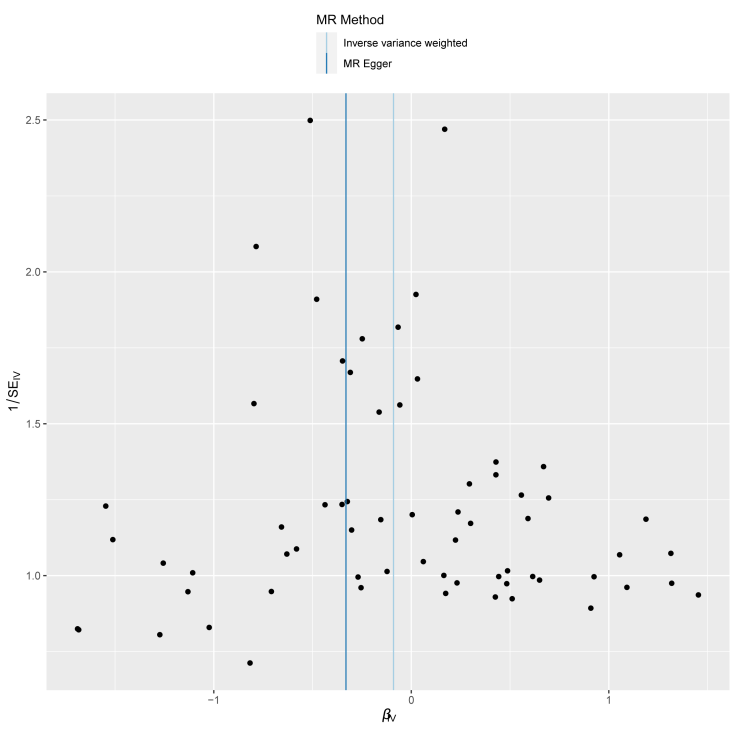
(f)
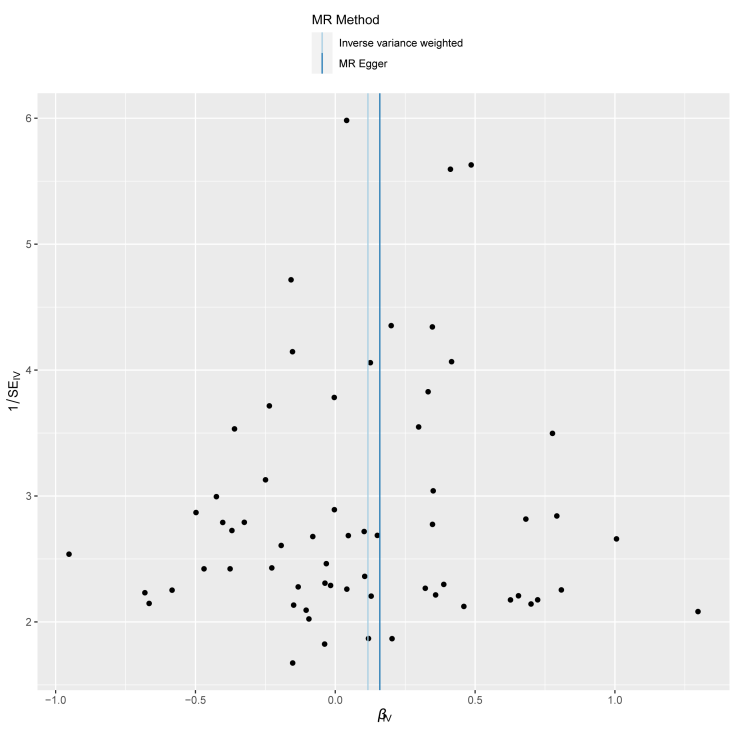
**

**(g)
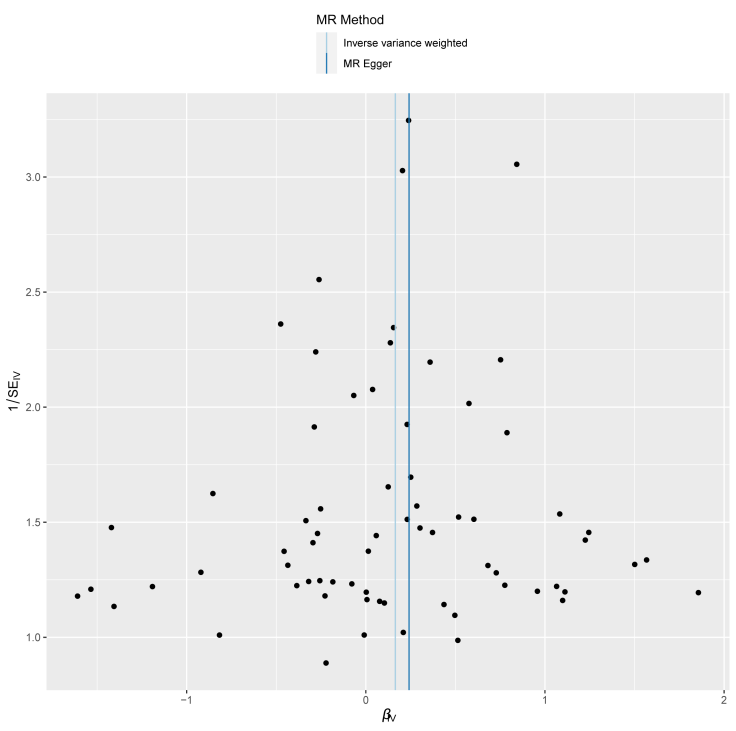
**

**Supplementary Figure 4** Funnel plot: (a) Allergic diseases and kidney stone, ureter stone or bladder stone; (b) Allergic diseases and other renal or kidney problem; (c) Allergic diseases and urinary tract or kidney infection; (d) Allergic diseases and kidney volume; (e) Allergic diseases and cyst of kidney; (f) Allergic diseases and chronic kidney disease; (g) Allergic diseases and unspecified kidney failure.
